# The pivotal role of immunometabolism in diabetic neuropathy and its potential therapeutic strategies

**DOI:** 10.3389/fendo.2026.1791057

**Published:** 2026-06-12

**Authors:** Run Lin, Xiangyuan Zhang, Xi Yang, Rumeng Tang, Tingting Bao, Linhua Zhao

**Affiliations:** 1 Institute of Metabolic Diseases, Guang’anmen Hospital, China Academy of Chinese Medical Sciences, Beijing, China; 2 Graduate College, Beijing University of Chinese Medicine, Beijing, China; 3 Department of Nephrology, the First Affiliated Hospital of Zhejiang Chinese Medical University (Zhejiang Provincial Hospital of Chinese Medicine), Hangzhou, Zhejiang, China; 4 Key Laboratory of Intelligent Chinese Medicine in the Prevention and Treatment of Metabolic Diseases, Beijing, China

**Keywords:** diabetic neuropathy, immune factor, immunocyte, immunometabolism, therapeutic strategies

## Abstract

Diabetic peripheral neuropathy (DPN) is a prevalent and severely disabling complication of diabetes mellitus characterized by complex pathophysiological mechanisms. Beyond the metabolic disorder induced by glucolipotoxicity, DPN represents an immunometabolic dysregulation arising from the interaction between metabolic abnormalities and immune imbalance. This review comprehensively encapsulates recent advances in the understanding of DPN through the lens of immunometabolism. Initially, classical pathophysiological mechanisms are discussed, demonstrating that persistent hyperglycemia and lipotoxicity activate the polyol pathway, promote advanced glycation end products formation, and lead to mitochondrial dysfunction, which collectively inflict structural and functional damage to neurons, Schwann cells, and neurovascular units. Furthermore, neuroinflammation in DPN transcends the peripheral nerve-dorsal root ganglion-spinal cord axis, with immune cell activation and inflammatory microenvironment formation directly perpetuating clinical symptoms such as hyperalgesia and hypoesthesia. This review further delves into the molecular basis of immunometabolic dysregulation, exploring oxidative stress from excessive reactive oxygen species, nitrative stress from nitric oxide signaling imbalance, and cytokine-mediated inflammatory amplification involving TNF-α, IL-1β, and IL-6. The role of intestinal dysbiosis in shaping systemic immune responses through metabolite anomalies also receives attention, contributing to the neuropathic pathology. These interconnected pathways foster a pathological positive feedback loop. In addition, the spatiotemporal dynamics of immune cells like monocytes/macrophages, T cells, B cells, microglia, and mast cells in the context of DPN are scrutinized, highlighting metabolic reprogramming and pro-inflammatory phenotypic shifts under hyperglycemic conditions. The review elucidates the complex crosstalk network between immune cells and non-immune cells, such as Schwann cells and vascular endothelial cells, which centralizes neuroinflammation regulation in DPN. Finally, potential therapeutic strategies focusing on immunometabolism are summarized, offering prospects for clinical translation. This immunometabolic perspective proves crucial in refining intervention regimens for DPN. In conclusion, immunometabolic dysregulation underpins the pathological progression of DPN, providing a comprehensive theoretical foundation for understanding its complex pathology and developing targeted therapeutic strategies.

## Graphical Abstract

**Figure:**
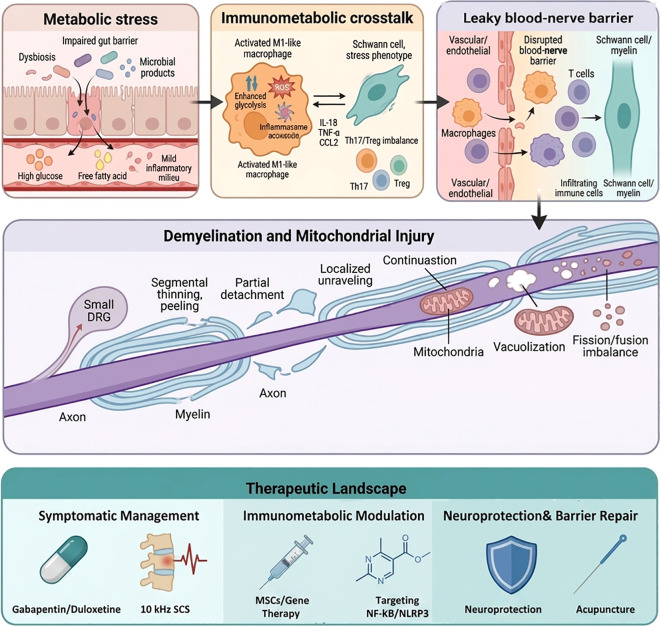

## 1 Introduction

Diabetic neuropathy, particularly diabetic peripheral neuropathy (DPN), is among the most common and disabling complications of diabetes. Clinically, DPN is characterized by abnormal sensation, neuropathic pain, sensory and/or motor dysfunction, and an increased risk of foot ulceration and amputation, all of which substantially impair quality of life and impose a considerable public health burden. Affecting a large proportion of the diabetic population, DPN continues to rise worldwide, placing sustained pressure on healthcare systems and socioeconomic resources (1).

Traditionally, DPN has been primarily attributed to metabolic disturbances caused by hyperglycemia and lipotoxicity, including oxidative stress, mitochondrial dysfunction, accumulation of advanced glycation end products, microvascular ischemia and hypoxia, and axon–myelin structural injury. However, growing evidence suggests that “glucotoxicity” and “lipotoxicity” alone cannot fully explain the marked heterogeneity of DPN, including differences in pain phenotype, disease progression, and regenerative failure. Persistent metabolic stress induces chronic low-grade inflammation and reshapes immune responses, thereby driving DPN into a pathological state in which metabolic abnormalities and immune dysregulation mutually reinforce one another, namely immunometabolic dysregulation (2). Immunometabolism concerns the reciprocal interplay between metabolic processes and immune responses. On the one hand, metabolic abnormalities such as hyperglycemia, hyperlipidemia, hypoxia, and mitochondrial stress can induce metabolic reprogramming in immune cells, including monocytes/macrophages, T cells, and microglia, thereby altering their activation, migration, polarization, and cytokine secretion. On the other hand, activated immune cells release inflammatory mediators, such as tumor necrosis factor-α (TNF-α), interleukin-1β (IL-1β), and interleukin-6 (IL-6), which further aggravate oxidative stress, endothelial dysfunction, blood–nerve barrier disruption, and axonal degeneration, ultimately establishing a self-amplifying neuroinflammatory cascade (3, 4). Available evidence from both patients with DPN and animal models shows increased infiltration of immune cells, including macrophages and T cells, in peripheral nerves, along with enhanced microglial activation in the spinal cord and dorsal root ganglia, elevated TNF-α levels, activation of poly(ADP-ribose) polymerase 1 (PARP-1), and dysregulation of multiple inflammatory signaling pathways (5, 6). Collectively, these findings indicate that DPN is not merely a metabolically driven nerve injury, but rather a chronic neurodegenerative disorder with substantial immune involvement. Notably, interventions targeting these immune cells and immunoregulatory mediators have been shown to alleviate DPN-associated injury to some extent (7).

It is further worth emphasizing that immunometabolic dysregulation in DPN does not exist in isolation, but is closely related to redox imbalance and host–microbiome interactions. Hyperglycemia and lipotoxicity can lead to excessive generation of reactive oxygen species (ROS) and disordered nitric oxide (NO) signaling, thereby inducing mitochondrial injury, protein nitration, lipid peroxidation, and DNA damage, and amplifying inflammatory responses through pathways such as nuclear factor kappa-B (NF-κB), mitogen-activated protein kinase (MAPK), and the NOD-like receptor family pyrin domain-containing 3 (NLRP3) inflammasome. Meanwhile, gut microbiota dysbiosis and abnormal microbial metabolites, such as increased lipopolysaccharide (LPS) and reduced short-chain fatty acids (SCFAs), may also participate in the development and progression of DPN by damaging the intestinal barrier, activating Toll-like receptor (TLR) signaling, regulating immune-cell polarization, and affecting systemic oxidative stress status. In other words, there is a highly intertwined pathological relationship among microbiota-derived signals, redox pathways, and immunoinflammatory networks, and this integrative framework may be key to explaining the heterogeneity, chronicity, and differential treatment responses of DPN. In addition, the specific roles of different immune-cell subsets in DPN, the causal relationships between their metabolic phenotypes and nerve injury, and the translational value of these mechanisms remain to be further clarified. Therefore, it is necessary to re-examine the pathological mechanisms and therapeutic strategies of DPN within a more comprehensive theoretical framework.

Against this background, the present review aims to comprehensively summarize the main recent advances in DPN research from an immunometabolic perspective. Specifically, it focuses on the roles of immune cells and their metabolic reprogramming in disease initiation and progression, integrates the mechanistic links among redox biology, cytokine networks, and gut microbiota-derived signals, and further discusses current and emerging intervention strategies together with their translational potential. By establishing a more integrative analytical framework, this review seeks to provide a theoretical basis for understanding the complex pathophysiology of DPN and for identifying more precise therapeutic strategies.

## 2 Pathophysiological basis of DPN: an immunometabolic perspective

### 2.1 Classical pathophysiological mechanisms of DPN

DPN arises from the interplay of multiple pathogenic factors, with persistent hyperglycemia and associated lipotoxicity serving as the central metabolic drivers. Hyperglycemia triggers several interrelated pathogenic pathways, including increased flux through the polyol pathway, activation of protein kinase C (PKC), accumulation of advanced glycation end products (AGEs), mitochondrial dysfunction, and enhanced oxidative stress, ultimately leading to structural and functional injury to neurons, Schwann cells, and the neurovascular unit. Meanwhile, diabetes-associated microangiopathy results in reduced perfusion of neural tissue, local ischemia and hypoxia, and impaired nutrient delivery, thereby further exacerbating axonal degeneration, demyelination, and abnormalities in nerve conduction.

Among these classical mechanisms, the AGE–RAGE axis is considered a key link between metabolic stress and chronic tissue injury. Nerve biopsy studies in patients with DPN have shown that AGEs are predominantly deposited in axons and myelin within neural tissue (3, 8). AGEs are a heterogeneous group of molecules generated through nonenzymatic reactions between glucose or other sugar derivatives and proteins or lipids, and they can bind to the receptor for advanced glycation end products (RAGE). AGE–RAGE signaling not only enhances oxidative stress and endothelial dysfunction but also activates inflammatory pathways such as NF-κB, promotes the expression of chemokines and pro-inflammatory cytokines, and thereby amplifies local neuroinflammation (9). In addition, chronic hyperglycemia may compromise the integrity of the blood–nerve barrier (BNB), facilitate the infiltration of circulating inflammatory cells into neural tissue, and alter the glycation status and antigenicity of myelin proteins, potentially rendering them more susceptible to immune recognition and attack (10, 11). Therefore, DPN is not simply a direct consequence of glucotoxicity alone, but rather the integrated outcome of metabolic abnormalities, microangiopathy, oxidative stress, and inflammatory responses.

### 2.2 Neuroinflammation in peripheral nerves, dorsal root ganglia, and the spinal cord

A growing body of evidence from both human and animal studies indicates that DPN-related pathology is not confined to distal peripheral nerves, but involves neuroimmune abnormalities at multiple levels, including peripheral nerves, the dorsal root ganglia (DRG), and the spinal cord (12, 13). In peripheral nerves, chronic hyperglycemia and lipotoxicity promote immune-cell infiltration and Schwann-cell stress, thereby establishing a persistent local inflammatory microenvironment. Studies in patients with DPN and in experimental models have demonstrated increased recruitment of myeloid cells, particularly macrophages, together with elevated levels of pro-inflammatory mediators such as TNF-α, IL-1β, and inducible nitric oxide synthase (iNOS), as well as reduced expression of anti-inflammatory and repair-associated factors (14). These findings suggest that the peripheral nerve microenvironment in DPN shifts from homeostasis toward a pro-inflammatory and maladaptive state.

In the early stage of DPN, the DRG may exhibit increased infiltration of inflammatory cells accompanied by sensory hypersensitivity; as the disease progresses, immune activation within the DRG and related sensory pathways becomes more pronounced and is associated with persistent pain and progressive sensory dysfunction (15). Previous studies have reported increased neutrophil infiltration and sensory hypersensitivity during the early phase of DPN, whereas disease progression is accompanied by enhanced recruitment of T cells and other inflammatory cells, together with more severe sensory loss and maintenance of chronic pain. These temporal changes indicate that the DRG is not merely a passive target of injury, but may serve as a neuroimmune hub that drives pain amplification and sensory imbalance. In addition to peripheral nerves and the DRG, activation of central glial cells also contributes importantly to DPN. Spinal microglia and astrocytes can be activated by hyperglycemia and peripheral inflammatory signals and, through pathways such as TLR4/MyD88/NF-κB, p38 MAPK, and the NLRP3 inflammasome, release TNF-α, IL-1β, IL-6, and chemokines, thereby promoting central sensitization and persistent neuropathic pain. Therefore, DPN should be regarded as a continuous neuroinflammatory process spanning the peripheral nerve–DRG–spinal cord axis, rather than merely a degenerative disorder of distal peripheral nerves.

### 2.3 Immunometabolic dysregulation in DPN and its molecular basis

Although traditional mechanisms have laid the foundation for understanding DPN, increasing evidence indicates that explaining DPN solely in terms of glucotoxicity and lipotoxicity is insufficient to fully encompass its marked heterogeneity in pain phenotype, rate of progression, tissue repair capacity, and treatment response (3, 16). It is now widely recognized that the onset and progression of DPN result not from a single metabolic disturbance, but from the integrated effects of persistent hyperglycemia, disordered lipid metabolism, impaired insulin signaling, mitochondrial dysfunction, redox imbalance, and chronic low-grade inflammation (17–20). Within this context, a complex network of interactions emerges among DRG sensory neurons, Schwann cells, endoneurial microvascular endothelial cells, and immune cells. Under chronic metabolic stress, functional dysregulation of these cellular components disrupts the local neural microenvironment, leading to redox imbalance, cytokine disequilibrium, neurovascular unit dysfunction, and impaired nerve-repair capacity, thereby promoting the transition from early functional abnormalities to overt structural injury in DPN (21–23).

A central feature of this process is immunometabolic dysregulation in both resident and infiltrating immune cells. Under pathological conditions such as hyperglycemia, hyperlipidemia, and hypoxia, monocytes/macrophages, T cells, and glial cells undergo marked metabolic reprogramming, characterized by enhanced glycolysis, disturbed mitochondrial oxidative phosphorylation, altered fatty-acid metabolism, and activation of inflammation-associated metabolic pathways (24, 25). These metabolic alterations drive immune cells toward a pro-inflammatory phenotype, upregulate mediators such as TNF-α, IL-1β, IL-6, and inducible nitric oxide synthase (iNOS), and concurrently attenuate anti-inflammatory and reparative signals, including interleukin-10 (IL-10) and transforming growth factor-β (TGF-β) (14). As a result, immunometabolic imbalance not only directly injures neurons and Schwann cells, but also accelerates lesion progression by impairing neural blood flow, disrupting the blood–nerve barrier, promoting myelin degradation, and inhibiting nerve regeneration.

In addition, this framework may also help explain the striking clinical heterogeneity of DPN. Some patients predominantly present with persistent neuropathic pain, whereas others are characterized mainly by sensory loss and progressive neural dysfunction (26). Such differences suggest that the dynamic interplay among immune-cell subsets, metabolic states, and the tissue microenvironment may shape distinct clinical phenotypes of DPN. Accordingly, re-examining DPN through an immunometabolic lens may not only integrate traditional metabolic theories with inflammatory mechanisms, but also provide a conceptual basis for identifying disease-stratification biomarkers and developing more precise therapeutic strategies.

#### 2.3.1 Oxidative stress signaling related to reactive oxygen species

Oxidative stress is widely recognized as one of the major pathological mechanisms underlying DPN and may serve as a critical link between metabolic abnormalities and immune activation (27). Under diabetic conditions, persistent hyperglycemia increases substrate flux through the mitochondrial electron transport chain, leading to enhanced electron leakage and superoxide generation (28); Meanwhile, activation of the polyol pathway converts glucose to sorbitol via aldose reductase and consumes reduced nicotinamide adenine dinucleotide phosphate (NADPH), thereby impairing glutathione regeneration and weakening cellular antioxidant defenses (29). In addition, enhanced AGEs–RAGE signaling, activation of NADPH oxidase, and oxidative bursts from inflammation-related cells may all contribute to the sustained generation and accumulation of reactive oxygen species (ROS) (30, 31). During type 2 diabetes and prediabetes, elevated free fatty acids and high-fat-diet-induced lipotoxicity can likewise promote oxidative injury in peripheral nerves, suggesting that ROS generation in DPN may be jointly driven by glucotoxicity and lipotoxicity (32).

The detrimental effects of excessive ROS production on peripheral nerves are not limited to direct cytotoxicity. Excessive ROS can induce lipid peroxidation, oxidative protein modification, DNA damage, and loss of mitochondrial membrane potential, thereby impairing axonal conduction, myelin homeostasis, and endothelium-dependent vasodilation (28, 33). In DRG sensory neurons, mitochondrial dysfunction and insufficient adenosine triphosphate (ATP) supply may compromise long-axon transport, peripheral sensory innervation, and post-injury regenerative processes (34). Oxidative injury in Schwann cells may weaken their metabolic support for axons and impair their capacity to maintain myelin, whereas endothelial-cell injury can reduce local perfusion and induce ischemia and hypoxia, thereby further increasing the vulnerability of neural tissue (33). Notably, ROS are not only a major consequence of metabolic injury, but may also amplify inflammatory responses. Excessive ROS can activate NF-κB, MAPK, and other inflammation-related signaling pathways, induce the expression of inflammatory mediators such as TNF-α, IL-1β, and IL-6, and thereby promote the recruitment and pro-inflammatory activation of multiple immune-cell types. In turn, activated immune cells can generate additional ROS through NADPH oxidase (NOX) and mitochondrial pathways (14, 35). Therefore, in DPN, oxidative stress should be regarded not only as an important downstream manifestation of metabolic dysregulation, but also as an amplifier of chronic neuroinflammation and disease progression.

#### 2.3.2 Imbalance of nitric oxide signaling and nitrosative stress

NO exerts complex biological effects in the peripheral nervous system. Under physiological conditions, NO is generated in appropriate amounts mainly by endothelial nitric oxide synthase (eNOS) and neuronal nitric oxide synthase (nNOS), where it participates in the regulation of local neural blood flow, maintenance of vasomotor balance, and modulation of nerve conduction (23, 36). Under diabetic conditions, however, NO signaling balance can be markedly disturbed: on the one hand, hyperglycemia, oxidative stress, and endothelial dysfunction can lead to reduced eNOS activity or uncoupling, thereby decreasing the bioavailability of protective NO; on the other hand, the inflammatory milieu can induce increased expression of inducible nitric oxide synthase (iNOS), thereby promoting pathological nitrosative stress (37). Relevant studies suggest that nitrosative stress may further activate poly(ADP-ribose) polymerase 1 (PARP-1), resulting in depletion of nicotinamide adenine dinucleotide (NAD+) and ATP and ultimately leading to transcriptional abnormalities, energy failure, and cellular injury (38, 39). In DPN, this process may aggravate Schwann-cell injury, axonal degeneration, and dysfunction of the neural microvascular unit. Importantly, NO/ROS imbalance is not an isolated redox event, but is closely connected to inflammatory networks. Pro-inflammatory cytokines such as TNF-α can promote iNOS expression, whereas peroxynitrite formation and PARP activation may further enhance inflammatory signaling and impair mitochondrial function, suggesting the presence of a mutually reinforcing pathogenic loop.

#### 2.3.3 Cytokine-mediated inflammatory amplification

In DPN, the cytokine network plays a pivotal role in the transition from metabolic stress to chronic inflammation. TNF-α, IL-1β, and IL-6 are among the key mediators most extensively studied to date. Available evidence suggests that TNF-α exerts multiple pathological effects in DPN. It not only promotes inflammation-related cellular and molecular responses, but also affects sensory-neuron excitability and contributes to pain sensitization (40). IL-1β is frequently associated with activation of inflammation-related signaling pathways and can amplify local neuroinflammation while enhancing neuronal responsiveness to injurious stimuli (35). IL-6 exhibits both pro-inflammatory and immunoregulatory properties and may exert sustained effects on the maintenance of chronic inflammation and pain sensitization. In addition, several chemotaxis-related molecules may participate in the recruitment of immune cells, including monocytes/macrophages and T cells, to peripheral nerves and the DRG, thereby promoting the persistence of local inflammation (41).

#### 2.3.4 Immunometabolic regulation involving the gut microbiota

Hyperglycemia-induced gut microbiota dysbiosis may influence immune responses through altered microbial metabolites and thereby contribute to neuropathy in DPN. A clinical randomized controlled trial showed that fecal microbiota transplantation from healthy donors significantly alleviated distal symmetric polyneuropathy (DSPN). Mechanistically, an increase in microbiota with greater butyrate-producing capacity, together with a reduction in microbiota enriched for endotoxin synthesis pathway genes, improved intestinal barrier integrity and reduced the levels of the pro-inflammatory cytokines TNF-α and IL-6 (42). Butyrate can exert anti-inflammatory effects by acting directly on intestinal epithelial cells and immune cells (43), and the reduced capacity of the diabetic gut microbiota to produce short-chain fatty acids may therefore be associated with inflammation-related neuropathy. Another study showed that serum lipopolysaccharide (LPS) levels were significantly higher in patients with DPN than in patients with diabetes mellitus (DM) and healthy controls. Gut microbiota dysbiosis may induce systemic endotoxemia and impair intestinal barrier function, which in turn stimulates pattern-recognition receptors such as TLR4, activates macrophages, and triggers a pro-inflammatory cascade involving cytokines such as TNF-α and IL-6, leading to peripheral axonal injury, glial activation, and mitochondrial dysfunction (44, 45). Experimental evidence further suggests that the dietary fiber inulin alleviates peripheral neuropathy in db/db mice by regulating the gut microbiota and its metabolites, thereby inhibiting inflammation.

## 3 Roles of immune cells in the pathogenesis of DPN

Immune-cell activation and metabolic dysregulation are important drivers of neuroinflammation in DPN. Clinically, neutrophil and monocyte counts in patients with painful DPN are positively correlated with chronic neuropathic pain (46). Compared with healthy controls, sciatic nerve tissue from patients with DPN shows significant alterations in immune-cell infiltration, including increased infiltration of M1 macrophages and resting CD4+ memory T cells, together with decreased infiltration of M2 macrophages, resting mast cells, monocytes, and follicular helper T cells. In addition, differentially expressed genes are enriched in immune-related pathways such as leukocyte migration and chemotaxis (47). Similar immune alterations have also been observed in sciatic nerve tissue from DPN mice, including a marked increase in activated CD68+ macrophages, significantly elevated levels of TNF-α, IL-1β, and iNOS, and significantly reduced levels of arginase-1, IL-10, and TGF-β proteins (48). Moreover, neuroimmune interactions in the DRG also participate in DPN pathogenesis. Early-stage DPN mice show increased neutrophil infiltration in the DRG, whereas late-stage DRG tissues exhibit increased infiltration of both neutrophils and T cells. Early DPN is characterized predominantly by hypersensitivity, whereas late DPN shows prominent sensory loss; however, both stages are accompanied by significant tonic pain (49). These findings indicate that DPN is not merely a metabolic nerve injury, but rather a neuroinflammatory pathological process involving the spatiotemporal participation of multiple immune-cell types.

### 3.1 Monocytes and macrophages

#### 3.1.1 Monocytes

Monocytes are immature phagocytic precursors that are found mainly in peripheral blood and serve as transitional cells linking circulating immunity with tissue immunity. Their migration and differentiation, as well as the initiation of inflammatory signal transduction, play important roles in the progression of DPN.

##### 3.1.1.1 Migration and differentiation of monocytes

Under diabetic conditions, monocyte infiltration into the spinal cord and sciatic nerve is increased. These cells subsequently differentiate into macrophages, leading to sensory abnormalities, pain, and neural injury. This migration is associated with upregulated expression of monocyte chemoattractant protein-1 (MCP-1) (50). Studies have shown that hyperglycemia can aggravate DPN-related axonal injury by enhancing sirtuin 3 (SIRT3)-mediated deacetylation and activation of sterile alpha and TIR motif containing 1 (SARM1). In a traumatic sciatic nerve injury model, SARM1 further promotes DRG neurons to upregulate chemokines such as MCP-1 through the c-Jun N-terminal kinase/c-Jun (JNK–c-Jun) signaling pathway and induces accumulation of CD11b+ immune cells in the DRG. Therefore, current evidence suggests that the hyperglycemia–SARM1 axis may participate in enhanced MCP-1 signaling and subsequent monocyte recruitment in DPN (51, 52). Meanwhile, hyperglycemia can also directly induce Schwann cells to upregulate MCP-1 and activate NF-κB/RAGE-related inflammatory responses (53), and MCP-1 expression is elevated in the sciatic nerves of diabetic rats together with leukocyte infiltration (54), These findings indicate that, under diabetic conditions, a chemotactic microenvironment favorable for monocyte recruitment is already established locally in peripheral nerves.

MCP-1 promotes the redistribution and recruitment of monocytes/mononuclear phagocytes to inflammatory sites. In DPN mouse models, CD68+ monocytes/macrophages are increased in the spinal cord and sciatic nerve and are correlated with neurobehavioral outcomes, whereas promotion of macrophage polarization toward the anti-inflammatory M2 phenotype can alleviate sciatic nerve inflammation and improve neural function (55). Compared with nondiabetic mice, streptozotocin (STZ)-induced DPN mice show increased infiltration of blood monocyte-derived macrophages in the spinal cord (56); Depletion of peripheral monocytes can simultaneously reduce IL-1β and TNF-α expression and relieve mechanical allodynia, indicating that monocyte recruitment is a functional component of pain generation in DPN. A machine-learning study also showed that, in a painful DPN cohort, monocyte counts were positively correlated with the presence of chronic neuropathic pain (46).

##### 3.1.1.2 Metabolic alterations of monocytes under hyperglycemic conditions

In the peripheral blood of patients with type 2 diabetes mellitus (T2DM), monocyte numbers are significantly increased, accompanied by subset redistribution characterized by decreased classical monocytes and increased intermediate monocytes (57). Classical monocytes, as inflammatory scavengers, can phagocytose pathogens and apoptotic cells and release anti-inflammatory factors such as IL-10, whereas intermediate monocytes exert pro-inflammatory effects, secrete cytokines such as TNF-α and IL-6, and participate in inflammatory infiltration of tissues. These findings suggest that, under hyperglycemic conditions, monocyte lineage composition shifts toward a pro-inflammatory phenotype and the body enters a state of chronic low-grade inflammatory activation.

*In vitro* studies have shown that incubation of monocytes with glucose shifts them toward a more glycolytic phenotype (58). Hyperglycemia not only alters monocyte energy allocation, but also promotes their activation toward pro-inflammatory M1 macrophages through endoplasmic reticulum stress and initiates inflammatory signaling mediated by phosphorylated c-Jun N-terminal kinase (p-JNK) (59); At the same time, endoplasmic reticulum stress increases ROS synthesis by upregulating NADPH oxidase, leading to enhanced endothelial-cell adhesion and lipid uptake. Peripheral blood monocytes from patients with T2DM show elevated ROS levels, and their plasma can also induce ROS production in THP-1 cells, indicating that this process may be attributable, at least in part, to circulating signals (60).

Hyperglycemia stimulates monocytes to produce more ROS, increases RAGE mRNA expression, and reduces secretion of soluble RAGE (sRAGE), thereby promoting oxidative stress (61). sRAGE can capture circulating pro-inflammatory ligands, consists of two isoforms—endogenous secretory RAGE (esRAGE) and cleaved RAGE (cRAGE)—and can inhibit the hyperglycemia-induced increase in IL-1β secretion. By interfering with the nuclear translocation of nuclear factor erythroid 2-related factor 2 (Nrf2), hyperglycemia suppresses antioxidant enzymes, increases oxidative stress in monocytes, and simultaneously inhibits the expression and release of cRAGE. Nrf2 is a redox-sensitive transcription factor that protects cells against toxicity and oxidative stress by regulating the expression of genes involved in detoxification and antioxidant defense (62). Impaired nuclear translocation of Nrf2 and its adverse effects have been observed in diabetic retinopathy (63), whereas in DPN mice, increased Nrf2 expression has been shown to alleviate neural injury and inflammation. Further mechanistic studies have shown that hyperglycemia induces ferroptosis in Schwann cells by inhibiting Nrf2 signaling (64, 65).

##### 3.1.1.3 Initiation of inflammation in DPN

The inflammatory state of DPN is associated with increased TLR4 expression and decreased caveolin-1 (Cav-1) expression in monocytes. Toll-like receptors (TLRs) are transmembrane pattern-recognition receptors expressed on multiple cell types, including monocytes. Upon recognizing specific ligands, TLRs trigger the release of pro-inflammatory cytokines and promote inflammatory conditions in T2DM and its complications. Cav-1 is a 22-kDa membrane-associated protein that regulates receptor signaling by directly binding receptors or downstream molecules and is regarded as an important regulator of innate immunity and inflammation. Studies have found that, in the peripheral blood of patients with T2DM complicated by DPN, monocyte expression of TLR4 and MYD88 is higher than that in patients with T2DM alone and in nondiabetic individuals, whereas Cav-1 levels are reduced. Plasma TNF-α and IL-6 levels are elevated, positively correlated with TLR4, negatively correlated with Cav-1, and TLR4 concentrations are negatively correlated with caveolin-1 (66). These results indicate that systemic inflammation in T2DM-related peripheral neuropathy may be associated with enhanced TLR4/MyD88 signaling and reduced Cav-1-mediated inhibition in monocytes. TLR4 can trigger a MYD88- and NF-κB-dependent signaling cascade, leading to the release of pro-inflammatory cytokines such as TNF-α and IL-6 and thereby aggravating inflammation (67). Evidence further indicates that upregulation of Cav-1 in mouse macrophages significantly inhibits the production of TNF-α and IL-6 and can exert anti-inflammatory effects by directly binding TLR4 and functionally suppressing ROS production and pro-inflammatory cytokine secretion (68). Elevated glucose, however, reduces both the number and size of Cav-1-positive structures in monocytes and suppresses Cav-1 expression (69).

Hyperglycemia also induces increased secretion of the pro-inflammatory cytokine IL-1β by monocytes. This process is mediated mainly through activation of protein kinase C (PKC) signaling, with the participation of p38 MAPK and extracellular signal-regulated kinase (ERK) phosphorylation, whereas JNK does not appear to be involved. In addition, inhibition of NADPH oxidase or NF-κB abolishes hyperglycemia-induced IL-1β secretion by monocytes. Further studies have shown that hyperglycemia can induce inflammatory gene expression by altering transcriptional mechanisms, increasing recruitment to the IL-1β promoter, promoting histone acetylation and methylation at the IL-1β promoter, and reducing histone deacetylase activity in monocytes, thereby facilitating chromatin remodeling and transcription (70). IL-1β can stimulate cyclooxygenase-2 (COX-2) expression in rat neurons and glial cells, thereby promoting prostaglandin E2 (PGE2) synthesis (71). When COX-2 catalyzes the synthesis of PGE2 from arachidonic acid, carbon-centered radicals are generated, and PGE2 acting on the EP receptor family further promotes mitochondrial ROS generation and activates the caspase-3 cascade, thereby aggravating oxidative stress and inflammatory responses in neural tissue (72, 73). IL-1β can also upregulate p38 MAPK, induce iNOS phosphorylation in spinal microglia and astrocytes, increase NO release into the cerebrospinal fluid, and induce thermal hyperalgesia in rats (74). Animal experiments have shown that IL-1β and TNF-α expression is elevated in the sciatic nerves and spinal cords of mice with painful DPN; after treatment, neuropathic pain is relieved, accompanied by reductions in IL-1β and TNF-α levels, suggesting that both cytokines participate in the development of DPN-induced neuropathic pain (56, 75). At the same time, studies have demonstrated that anti-IL-1 therapy protects myelin and axonal structure in the sciatic nerves of DPN rats (76).

#### 3.1.2 Macrophages

Macrophages, as core effector cells of the immune system, are generally divided into two major functional subtypes: classically activated macrophages (M1) and alternatively activated macrophages (M2). In addition, intermediate phenotypes regulated by the tissue microenvironment may also exist. M1 macrophages eliminate pathogens and mediate inflammatory responses by secreting pro-inflammatory cytokines such as TNF-α and IL-1β, as well as toxic mediators such as ROS and NO. By contrast, M2 macrophages highly express anti-inflammatory molecules such as arginase-1 (Arg-1) and TGF-β, together with tissue repair-related factors, and participate in the resolution of inflammation, tissue remodeling, and immune regulation. The dynamic balance between M1 and M2 macrophages is therefore crucial for maintaining tissue microenvironment homeostasis. In DPN, hyperglycemia and metabolic dysregulation disrupt macrophage subset balance, leading to excessive activation of M1 macrophages and their infiltration into neural tissue, where they aggravate neuroinflammation, oxidative stress, and myelin injury and inhibit nerve regeneration through the persistent release of pro-inflammatory factors and ROS. By contrast, the anti-inflammatory and reparative functions of M2 macrophages are relatively weakened. This imbalance contributes to the pathological progression of DPN.

##### 3.1.2.1 Immunometabolic reprogramming of macrophages and polarization toward a proinflammatory phenotype

Under hyperglycemic conditions, macrophages show enhanced proliferative responses and upregulated proinflammatory gene expression (77, 78). Their metabolism shifts from oxidative phosphorylation to aerobic glycolysis, with increased glucose uptake and conversion to lactate; ATP production becomes less efficient but more rapid in order to meet the energy demands of the pro-inflammatory phenotype (79). During this process, the tricarboxylic acid cycle coupled to oxidative phosphorylation is disrupted at the levels of citrate and succinate, inducing reverse electron transport and triggering increased mitochondrial ROS generation (19), thereby activating multiple pathogenic pathways, including AGE formation, RAGE signaling, and PKC activation (80, 81). IL-1β expression is also increased. This metabolic reprogramming depends on activation of hypoxia-inducible factor-1α (HIF-1α), which leads to upregulation of the glucose transporter GLUT1 (79). Accumulation of succinate can also stabilize and activate HIF-1α, thereby further increasing IL-1β protein levels (82). Another study showed increased HIF-1α expression in pericapillary infiltrating macrophages and T cells in sural nerves from patients with diabetic peripheral neuropathy (83), further supporting the important role of HIF-1α in promoting pro-inflammatory metabolic alterations in macrophages.

Culture under hyperglycemic conditions reduces macrophage autophagic activity, resulting in accumulation of mitochondrial dysfunction characterized by high intracellular ROS levels; hyperglycemic stimulation also increases macrophage pyroptosis, promotes IL-1β production, and aggravates inflammatory responses (84). By activating the NLRP3 inflammasome and the MAPK/NF-κB pathways, ROS promote the release of pro-inflammatory factors such as TNF-α and IL-1β, further driving macrophage polarization toward the M1 phenotype and thereby forming a vicious cycle (85). In the setting of DPN, persistent hyperglycemia also increases ROS generation in other cell types, and degenerating axons in injured peripheral nerves also produce high levels of ROS (86). Elevated ROS levels have been reported in the sciatic nerves of DPN mice (87). Injury-induced ROS and chemokines secreted by Schwann cells recruit inflammatory cells, including monocytes and macrophages (88). By interfering with epigenetic reprogramming, ROS alter macrophage differentiation and favor induction of the pro-inflammatory M1 phenotype (85). Increased ROS released by macrophages can also activate transient receptor potential ankyrin 1 (TRPA1) channels on sensory neurons, thereby triggering pain responses (89, 90). Although ROS may also participate in axonal regeneration and functional recovery in neural tissue, and in acutely injured axons such ROS generation requires NOX2 complexes provided by macrophages recruited in a CX3CR1-dependent manner (91), DPN, by contrast, is characterized by distal axonal degeneration caused by chronic hyperglycemia and lipotoxicity, in which ROS more often act to promote neuroinflammation and oxidative stress.

Hyperglycemia can activate macrophages through multiple signaling pathways. Studies have demonstrated (92) that hyperglycemia mediates upregulation of Rho-associated kinase, which in turn activates mouse macrophages through the JNK/ERK pathway, resulting in a more pro-inflammatory phenotype and greater TNF-α production (93). In addition, Xu et al. revealed that TGF-β-activated kinase 1 mediates the ERK1/2, p38 MAPK, and NF-κB signaling pathways involved in activation of macrophages toward a proinflammatory phenotype under hyperglycemic conditions, accompanied by abnormally high expression of MCP-1 and TNF-α (94). The latest study by Fatema et al. found that repetitive intermittent hyperglycemia promotes M1 polarization and inflammatory responses in macrophages through the TLR4–interferon regulatory factor 5 (IRF5) pathway (95).

When oxidative stress persists over a prolonged period, macrophages accumulate large amounts of oxidized proteins and lipids, leading to metabolic dysfunction, phenotypic alteration, and cell death (86). Studies have shown that prolonged high-glucose exposure also impairs macrophage glycolytic capacity and glycolytic reserve, reduces NO and ROS production, and disrupts phagocytic and bactericidal activity. Although it does not affect the number or phenotype of bone marrow-derived macrophages, it increases IL-1β and TNF-α expression in M1 macrophages (96). These results indicate that, under long-term hyperglycemic stimulation, macrophages enter a stressed state in which they are prone to exaggerated responses to external stimuli, thereby secreting excessive inflammatory factors while exhibiting impaired phagocytic and bactericidal functions. This suggests that macrophage dysfunction contributes, at least in part, to the progressive aggravation of systemic inflammatory status.

##### 3.1.2.2 Roles of macrophages in DPN through the AGEs–RAGE signaling pathway

Long-term exposure to high glucose promotes AGE formation on myelin proteins in peripheral nerves. These AGEs are deposited in axons and myelin within neural tissue, are specifically recognized by macrophages through scavenger receptors, and are subsequently phagocytosed and attacked by macrophages, thereby promoting nerve demyelination. This phenomenon has been observed in neural tissue from rats with long-term diabetes (1.5–2.0 years), but not in rats with short-term diabetes (4–6 weeks) (97). Hyperglycemia can also induce macrophages to express higher levels of RAGE (98). Studies suggest that RAGE activation triggers pro-inflammatory responses in diabetic neural tissue by determining macrophage polarity. In the sciatic nerves of wild-type mice with type 1 diabetes, infiltration of iNOS+ M1 macrophages is increased and TNF-α mRNA expression is elevated. Subsequently, the TNF-α/JNK pathway impairs both anterograde and retrograde axonal transport through phosphorylation of motor proteins, while simultaneously reducing insulin sensitivity in neural tissue and causing atrophy of DRG neurons. By contrast, diabetic mice lacking RAGE exhibit increased numbers of M2 macrophages in the sciatic nerve, together with preserved neural function and insulin sensitivity (99).

After AGEs bind to RAGE on the cell surface, intracellular NADPH oxidase can be activated, leading to increased ROS production and subsequent upregulation of TLR4 expression on the macrophage surface (100, 101). At the same time, AGEs can bind to myeloid differentiation protein 2 (MD2) on macrophages and alter its conformation, thereby activating the pattern-recognition receptor TLR4 and forming an AGEs–MD2–TLR4 complex. This leads to recruitment of MyD88, activation of MAPK and NF-κB signaling, upregulation of TNF-α and IL-6 expression, and activation of inflammatory responses (67).

##### 3.1.2.3 Dysregulated secretion of inflammatory factors

In patients with diabetes, macrophages tend to maintain an M1-like phenotype, produce pro-inflammatory cytokines and proteases, promote oxidative stress through myelin degradation, and impede nerve regeneration (102). Analysis of sciatic nerves from DPN mice showed that pro-inflammatory markers of M1 macrophages, such as TNF-α and IL-1β, are increased, whereas anti-inflammatory markers of M2 macrophages, such as IL-10 and TGF-β, are decreased (48). Another study showed that macrophages derived from peripheral blood monocytes of patients with DPN display dysregulated cytokine production under basal conditions, characterized by lower production of IL-10 and MCP-1; macrophages from patients with type 2 diabetes show increased basal IL-6 production. However, after lipopolysaccharide (LPS) stimulation, macrophages from both the diabetes and DPN groups display attenuated induction of TNF-α, IL-6, and MCP-1, suggesting a dysfunctional state of “basal pre-activation but imbalanced responsiveness” (103).

TNF-α can directly induce nerve demyelination and can also stimulate monocytes and endothelial cells to secrete inflammatory mediators, thereby further aggravating neural injury (7). It can also promote the expression of multiple growth factors and cell-adhesion molecules, leading to endothelial dysfunction and injury to the vasa nervorum (104, 105). Further experiments found (8) that inhibition of TNF-α can delay DPN progression, markedly improve sciatic nerve morphology in DPN rats, and increase expression of myelin basic protein (MBP). MBP is a major constituent of myelin in the nervous system; it binds myelin lipids and promotes myelin formation. When nerves are injured, especially under demyelinating conditions, MBP expression decreases. Studies have shown that, compared with controls, MBP expression in the sciatic nerve myelin sheath of DPN rats is significantly reduced, indicating that TNF-α may promote nerve demyelination by reducing MBP expression. TNF-α can also increase Schwann-cell apoptosis, leading to peripheral nerve injury (106, 107). In addition, TNF-α can be produced by Schwann cells and endoneurial macrophages (108), act directly on DRG sensory neurons, activate neuronal p38 MAPK signaling, and enhance the sensitivity of peripheral nerve endings to pain (109). Similar to IL-1β, TNF-α can induce neuronal hyperexcitability by enhancing phosphorylation of NMDA receptors, thereby promoting the development and maintenance of neuropathic pain (110). Meanwhile, TNF-α stimulates monocytes and endothelial cells to secrete inflammatory factors such as IL-1β and IL-6, thereby indirectly amplifying their pathological effects (111).

In DPN, changes in TNF-α within the DRG are stage-dependent. In STZ-induced rat models, compared with healthy controls, TNF-α levels in the DRG are significantly increased at 2–5 weeks and continue to rise with prolongation of the disease course; by week 5, increased TNF-α protein expression is also observed in the spinal cord. These changes coincide with the early appearance and maintenance of mechanical allodynia and thermal hyperalgesia, indicating that peripheral sensitization occurs earlier than central sensitization and that persistent peripheral inflammation stimulates central spinal sensitization (112). Another study showed that, compared with controls, DPN mice at 6 weeks after STZ injection exhibit a significant increase in the number of M1 macrophages in the DRG (113). At 2 and 5 months after STZ injection, however, TNF-α concentrations in the DRG of DPN rats are significantly reduced, and IFN-γ and IL-6 are likewise decreased, accompanied by sensory loss and loss of plantar cutaneous nerve fibers. Further experiments showed that exogenous TNF-α can promote axonal growth of adult rat DRG sensory neurons through activation of NF-κB signaling, whereas this growth-promoting effect is markedly weakened in neurons derived from DPN animals (114). Elevated TNF-α levels in the DRG during early DPN are accompanied by increased macrophage infiltration; TNF-α is produced mainly by infiltrating M1 macrophages, activated Schwann cells, and neurons themselves, reflecting inflammatory activation at this stage. By contrast, reduced TNF-α levels in the DRG during late DPN may be related to functional impairment of immune cells, Schwann cells, and neurons.

Compared with patients with type 2 diabetes alone, patients with DPN have lower levels of TGF-β (115). TGF-β plays a dual role in peripheral nerve regeneration, promoting Schwann-cell growth and recruitment while at the same time inhibiting remyelination of regenerating axons. This complex role makes TGF-β a potential therapeutic target in peripheral nerve injury (116).

Compared with healthy controls, plasma IL-6 levels are significantly elevated in both T2DM and DPN patients and are higher in DPN patients than in those with T2DM alone (66). IL-6 expression is elevated in the sciatic nerves of DPN mice, and the AGE–RAGE–NF-κB axis mediates IL-6 expression and loss of pain perception in DPN (117). IL-6 exacerbates inflammatory responses by mediating TNF-α and IL-1β expression through signaling pathways such as JAK2/STAT3 and ERK and amplifies the effects of other cytokines associated with neuropathic pain (118). Studies have shown that serum IL-6 levels are higher in patients with painful DPN than in those with painless DPN (119). Although IL-6 is usually considered a pro-inflammatory cytokine, it may also possess anti-inflammatory properties and contribute to suppression of inflammatory responses and promotion of tissue repair. Low-dose IL-6 has been shown to significantly improve neural function and morphology. Subcutaneous IL-6 injection improved multiple indicators of neural dysfunction in DPN rats, including sensory and motor nerve conduction velocities, thermal hyperalgesia, tactile allodynia, and endoneurial blood perfusion in the sciatic nerve, and also prevented thinning of the nerve myelin sheath, although it was ineffective against mechanical hyperalgesia (120, 121). IL-6 may also promote myelination and nerve repair by acting directly on Schwann cells (122).

As an anti-inflammatory factor, IL-10 has shown bidirectional changes in DPN. IL-10 levels are lower in the sciatic nerves of 20-week-old DPN mice than in controls (48). A clinical study showed that 16% of patients with DPN had elevated serum IL-10 levels; these patients also had lower body mass index and more severe nerve-conduction dysfunction, whereas the remaining patients had decreased serum IL-10. This led to the inference that IL-10 activation may represent a neuroprotective response to severe peripheral nerve injury (123). Another study showed that IL-10 expression is upregulated in the sciatic nerves of both early- and late-stage DPN mice (8- and 24-week-old db/db mice) (124). Cross-species transcriptional network analysis likewise suggested activation of the IL-10 pathway in peripheral nerves from both patients with DPN and mice (125). Against a background of persistent metabolic stress and neural injury, the local neuroimmune microenvironment may repeatedly initiate compensatory anti-inflammatory and reparative programs. In peripheral nerve crush injury, IL-10—expressed mainly by macrophages—is markedly increased and participates in resolution of inflammation while supporting axonal regeneration (126). In DPN, however, such elevation of IL-10 is more likely to represent compensatory regulation in response to neural injury than effective protection capable of halting disease progression. In fact, as inflammatory diseases such as DPN shift from active inflammation to chronic inflammation, pro-inflammatory and anti-inflammatory pathways are often activated simultaneously. However, persistent hyperglycemia, lipotoxicity, oxidative stress, Schwann-cell injury, and immune-cell infiltration may allow pro-inflammatory effects to regain dominance. Therefore, even if IL-10 rises again in late-stage DPN, it may merely represent a passive response to chronic inflammatory injury and remain insufficient to reverse the continued progression of neural damage. IL-10 expression in DPN also varies across tissues. Studies have shown that reduced neuron-derived IL-10 in the DRG participates in pain generation in DPN mice; supplementation with exogenous IL-10 alleviates pain symptoms and reduces the expression of inflammatory mediators such as NGF, TNF-α, and iNOS in the DRG (127). DRG neurons also express the IL-10 receptor. Binding of IL-10 to its receptor can downregulate MCP-1 and reduce immune-cell recruitment mediated by DRG neuron-derived MCP-1, but this effect is weakened in DPN (128).

##### 3.1.2.4 Protective roles of macrophages in neuroinflammation in DPN

Macrophages are generally regarded as pathogenic in DPN, but accumulating evidence also indicates that macrophages within peripheral nerve tissue can exert certain neuroprotective effects. During the prodromal stage of DPN induced by a high-fat high-fructose diet (HFHFD), mice already exhibit thermal hypoalgesia, but no decrease in intraepidermal nerve fiber density, terminal axonal degeneration, or obvious demyelination has yet been detected. Resident macrophages (ResMacs) in the sciatic nerve display inflammation-activated transcriptional features, with upregulation of multiple chemokine-related genes together with increased MCP-1 expression in neural tissue, suggesting that they may participate in recruitment of CCR2+ recruited macrophages (RecMacs) (129). Use of CCR2 deficiency and pharmacological blockade reduces the number of RecMacs in the sciatic nerves of DPN mice and further aggravates thermal hypoalgesia, causing earlier onset of epidermal denervation. Transcriptomic analysis shows that these RecMacs display transcriptional features similar to those of macrophages in crushed nerves, highly express the galectin-3 (Gal-3) gene, and exert neuroprotective effects, possibly by clearing debris, excess fatty acids, or deoxy-sphingolipids that accumulate in nerves. Further studies show that Gal-3 deficiency accelerates degeneration of cutaneous sensory axons and aggravates thermal hypoalgesia. Gal-3 can recognize AGEs and promote their phagocytosis and clearance, thereby exerting neuroprotective effects (130). Earlier studies of experimental diabetic neuropathy observed monocyte/macrophage infiltration in the sciatic nerve accompanied by upregulation of IL-1β and p75NTR expression, suggesting that macrophages and IL-1β not only participate in neural injury in DPN, but may also attempt to promote nerve regeneration by inducing Schwann cells to upregulate p75NTR expression (131–133).

In DPN, macrophages not only promote inflammation but may also activate intrinsic protective responses. In STZ-induced DPN mouse models, macrophages expressing mesencephalic astrocyte-derived neurotrophic factor (MANF) are increased in the DRG and sciatic nerve, and both M1 and M2 macrophages are significantly elevated, although M1 macrophages greatly outnumber M2 macrophages. This finding suggests that M1 macrophages play a dominant role in neuroinflammation in DPN, whereas anti-inflammatory and reparative responses mediated by M2 macrophages coexist. However, progression of neural injury is driven mainly by persistent M1-mediated inflammation. Increased endoplasmic reticulum activity may trigger the surge in MANF protein observed in macrophages. MANF may function as a negative regulator of macrophage-triggered inflammation in the sciatic nerves of diabetic mice. Addition of recombinant human MANF reduces macrophage activity and related inflammatory factors by inhibiting the endoplasmic reticulum stress signaling pathway or the TLR4 signaling pathway, decreases infiltration of M1 macrophages in the DRG and sciatic nerve of diabetic mice without significantly affecting M2 macrophages, and further supports the view that neuroinflammation in DPN is dominated by M1 macrophages (113).

### 3.2 T cells

T cells comprise several core subsets, including CD4+ helper T cells (Th), CD8+ cytotoxic T cells (CTLs), and regulatory T cells (Treg), each of which exerts distinct biological functions through the secretion of specific cytokines and regulation by transcription factors (134). CD4+ Th cells can be further differentiated into subsets such as Th1, Th2, and Th17. Th1 cells secrete interferon-γ (IFN-γ) and participate in intracellular pathogen clearance and pro-inflammatory responses; Th2 cells mediate antiparasitic immunity and allergic responses; and Th17 cells release pro-inflammatory mediators such as IL-17 and IL-22 to trigger inflammatory responses in host defense, although excessive activation can aggravate inflammatory injury. CD8+ CTLs specifically eliminate target cells through the release of perforin and granzymes and through FasL expression, thereby maintaining immune surveillance against abnormal cells. Treg cells (CD4+CD25+Foxp3+) are a key anti-inflammatory subset that restrains excessive activation of effector T cells and maintains immune homeostasis by secreting inhibitory cytokines such as IL-10 and TGF-β and by contact-dependent suppression. Available evidence suggests that, among the adaptive immune abnormalities reported in DPN, T-cell-related pro-inflammatory skewing and insufficient immune regulation occupy an important place, mainly manifested as enhanced Th17-related inflammatory signaling, downregulation of Treg cells, and activation of cytotoxic T cells.

#### 3.2.1 Phenotypic changes in T cells

As an important T-cell subset that prevents excessive immune injury, reduced numbers and impaired function of Treg cells may play important pathogenic roles in DPN. Studies have shown that peripheral blood Treg-cell levels in patients with DPN are significantly lower than those in patients with type 2 diabetes alone and in healthy controls, and that the proportion of Treg cells is negatively correlated with the MDNS score (135). Another type 2 diabetes cohort likewise found that peripheral blood CD4+CD25+Foxp3+ Treg levels were lower in patients with peripheral neuropathy than in those without neuropathy, suggesting that Treg deficiency is associated with the occurrence and severity of DPN (136).

A hyperglycemic environment may further amplify disruption of immune homeostasis by directly impairing the structure and function of Treg cells (137). Mitochondria in Treg cells isolated from the peripheral blood of patients with type 2 diabetes and mild cognitive impairment show marked morphological abnormalities characterized by increased ROS production, opening of the permeability transition pore, and apoptosis; similar changes have also been observed in Treg cells from db/db mice. Further studies showed that culturing Treg cells under hyperglycemic conditions downregulates Foxp3 expression and reduces immunosuppressive activity. Hyperglycemia induces mitochondrial calcium overload in Treg cells by downregulating Na+/Ca2+/Li+ exchanger expression through specificity protein 1 (SP1) overexpression-mediated recruitment of histone deacetylase 2 (HDAC2) and histone deacetylation, thereby triggering mitochondrial oxidative injury, and O-glycosylation further strengthens this effect.

Accompanying the reduction in Treg cells is upregulation of Th17-related inflammatory factors. Peripheral blood interleukin-17A (IL-17A) and IL-6 levels in patients with DPN are significantly higher than those in patients with type 2 diabetes alone, and IL-17A levels are positively correlated with IL-6 levels. IL-17A and IL-6 are risk factors for the development and progression of DPN, whereas Treg cells are protective factors (138). The major source of IL-17A is Th17 cells, whereas IL-6 is an inflammatory cytokine that drives Th17 polarization. IL-6 is a key hub regulating the Th17/Treg balance: in the presence of TGF-β, IL-6 drives naïve CD4^+^ T cells to differentiate into Th17 cells and weakens the stability of Foxp3^+^ Treg cells, thereby promoting IL-17A secretion, whereas Treg cells can secrete IL-10 to inhibit this process (139). Another cross-sectional study also found elevated serum IL-17 levels in patients with DPN, and the TCSS score was positively correlated with IL-17, indicating that IL-17 is associated with DPN severity (140). Taken together, these findings suggest that disruption of the antagonistic balance between Treg cells and Th17 cells may contribute to the development of DPN.

IL-17A may mediate neural injury under diabetic conditions by affecting glial-cell activation and inflammatory responses in neural tissue. In animal models of diabetic retinopathy, hyperglycemia increases the expression of IL-17A and IL-17RA in Müller cells, which has also been validated *in vitro*. IL-17A and its receptor subsequently activate the Act1/TRAF6/IKK/NF-κB signaling pathway, thereby triggering neuroinflammation and retinal vascular injury (141). Studies have shown that IL-17A induces polarization of microglia and astrocytes and stimulates them to release neurotoxic mediators such as TNF-α, IL-1β, and IL-6, thereby triggering an inflammatory cascade (142). IL-17 can also act on neurons in the spinal cord and DRG, increase neuronal excitability, lead to peripheral and central sensitization, and promote pain formation (143). Based on the available evidence, IL-17A/IL-17 may amplify neuroinflammation and promote pain formation in the diabetic setting by activating neuroglial cells. However, IL-17A may not act solely as a pro-inflammatory damaging factor; its potential protective effects have also been observed in specific experimental models and disease stages. One study found that local IL-17A expression in the sciatic nerve is decreased during the early stage of STZ-induced diabetes in rats, and further *in vitro* experiments showed that IL-17A can enhance axonal plasticity and mitochondrial function in sensory neurons by activating the ERK and PI3K pathways, suggesting that, under certain conditions, it may exert neurotrophic-like or pro-regenerative effects (144).

Regarding T-cell phenotypic remodeling, CD4^+^CD28^null^ T cells may provide supportive evidence for a DPN-related process of inflammation-associated vascular injury. The number of CD4^+^CD28^null^ T cells in the peripheral blood of patients with DPN is significantly higher than that in healthy controls and in patients with type 1 diabetes mellitus (T1DM) without neuropathy (145). CD4^+^CD28^null^ T cells are a subset of CD4^+^ T lymphocytes with stronger pro-inflammatory properties and can produce high levels of IFN-γ, TNF-α, and IL-2. They can also release perforin and directly kill vascular endothelial cells (146). Cellular experiments have shown that intervention with CD4^+^CD28^null^ T cells under hyperglycemic conditions promotes apoptosis of microvascular endothelial cells, accompanied by increased secretion of IFN-γ and TNF-α (147). Previous studies have shown that IFN-γ inhibits collagen synthesis, thereby suppressing proliferation of vascular endothelial cells and accelerating apoptosis, while TNF-α can likewise aggravate inflammatory injury in vascular endothelial cells (148). Given the pathological background of impaired neural microvascular perfusion in DPN, these findings suggest that CD4^+^CD28^null^ T cells may indirectly contribute to chronic inflammatory status in DPN by damaging vascular endothelial cells and affecting the microvessels that nourish nerves, thereby aggravating peripheral neuropathy.

#### 3.2.2 CD8^+^ T cell-mediated cytotoxicity toward Schwann cells promotes diabetic peripheral neuropathy

In DPN, Schwann-cell cytotoxic injury mediated by CD8^+^ T cells has also been reported. High glucose induces increased expression of CXCR3 in CD8^+^ T cells through p38 MAP kinase. At the same time, hyperglycemic stimulation induces Schwann cells to produce large amounts of CXCL9, CXCL10, and CXCL11, which mediate infiltration of CD8^+^ T cells into diabetic peripheral neuropathic tissue through CXCR3. Results from Schwann cell and CD8^+^ T-cell coculture experiments show that Schwann cells promote activation of CD8^+^ T cells, whereas activated CD8^+^ T cells in turn express TNF-α, Fas ligand (FasL), and programmed death-ligand 1 (PD-L1), thereby exerting cytotoxic effects on Schwann cells (149). Taken together, these findings indicate that Schwann-cell-mediated chemotactic recruitment and activation of CD8^+^ T cells, followed by induction of Schwann-cell apoptosis, may directly drive demyelination, impaired axonal support, and reduced nerve conduction, representing an important T-cell-mediated injury pathway in the immunopathological mechanisms of DPN.

### 3.3 B cells

B cells contribute to DPN progression through both antibody-dependent and antibody-independent pathways. Their roles in DPN may involve autoantibody-mediated neural injury, defects in regulatory B cells (Bregs), secretion of pro-inflammatory factors, and interactions with other immune cells. Accordingly, targeting B cells and their related pathways may provide new therapeutic strategies for DPN.

#### 3.3.1 Autoantibody-mediated neural humoral immune injury

B cells participate in humoral immune responses by differentiating into plasma cells and producing antibodies. In various autoimmune neurological disorders, including diabetes-related neuropathies, studies have shown that patients’ sera contain neural-related antibodies, such as anti-ganglioside antibodies, and that these antibodies are significantly associated with peripheral nerve injury and clinical symptoms. A clinical study by Ge et al. reported that serum anti-ganglioside antibody levels were significantly elevated in patients with DPN and were positively correlated with DPN severity and inflammatory markers such as TNF-α and C-reactive protein (CRP), suggesting that humoral immune responses participate in the immunopathological mechanisms of DPN (150). In addition, an earlier study in patients with DPN found that, compared with controls, a higher proportion of patients with DPN were positive for antinuclear antibodies (ANA), further supporting a possible association between autoantibodies and neural injury (151). Regarding the potential pathogenicity of anti-ganglioside antibodies in peripheral nerve injury, a review by Willison et al. proposed that such antibodies participate in neural injury in multiple autoimmune peripheral neuropathies by directly targeting glycolipid structures on the neural membrane surface, although direct evidence for this mechanism in DPN still requires further validation (152).

#### 3.3.2 Induction of T-cell activation by B cells as antigen-presenting cells

In addition to their humoral immune functions, B cells also possess antigen-presenting capacity, which is crucial for regulating T-cell-mediated immune responses. The main characteristics of regulatory B cells (Bregs) include immunoregulatory mechanisms such as secretion of IL-10 and IL-35, expression of TGF-β, and expression of programmed death-ligand 1 (PD-L1). Breg cells suppress the activation and proliferation of autoreactive T cells by secreting IL-10, thereby helping to maintain immune tolerance to islet autoantigens (153). Under conditions of limited antigen availability, B cells can present antigen more efficiently than other APCs and promote activation of antigen-specific T cells because of their high-affinity antigen capture mediated by the B-cell receptor (BCR) (154). Extensive evidence from multiple autoimmune diseases indicates that B cells can continuously drive inflammatory T-cell responses through antigen presentation and may thereby aggravate chronic inflammatory injury, providing a mechanistic basis for understanding the potential role of B cells in immune-mediated neural injury.

#### 3.3.3 Protective effects of B-cell depletion

In NOD mice, B cells act as antigen-presenting cells and are critical for activating pathogenic T cells and initiating autoimmune diabetes; depletion of B cells can delay the onset of diabetes (155). Bregs mainly suppress activation of effector T cells, such as Th1 and Th17 cells, as well as other immune cells, by secreting anti-inflammatory cytokines or through cell-contact-dependent signals, thereby alleviating immune-mediated inflammatory responses. In multiple autoimmune animal models, Breg deficiency leads to more severe inflammatory injury, whereas enhancement of Breg function suppresses inflammatory T-cell responses, promotes Treg expansion, and improves disease manifestations (156). IL-10-producing Bregs can inhibit effector T cells and enhance regulatory T-cell function, and this protective effect has been demonstrated in multiple disease contexts, including infection and inflammatory disorders (157). Collectively, these findings suggest that B-cell depletion or impaired Breg function may differentially influence chronic immune-mediated injury depending on the specific immune context, whereas restoration or enhancement of Breg function may represent a potential immunomodulatory therapeutic strategy.

### 3.4 NK Cells

#### 3.4.1 NK cells and the neuroinflammatory microenvironment

Natural killer (NK) cells are key effector cells of the innate immune system. They can rapidly recognize and eliminate injured or stressed cells while also secreting cytokines such as IFN-γ and TNF-α to regulate the inflammatory microenvironment. In the diabetic setting, chronic hyperglycemia not only triggers oxidative stress and inflammation in neural tissue, but also causes immune dysregulation, thereby creating background conditions for neuroinflammation mediated by immune effector cells, including NK cells. DPN is typically characterized by a long-standing inflammatory state and accumulation of pro-inflammatory cytokines, and these inflammatory factors together contribute to a persistently activated neuroinflammatory microenvironment (158). Although direct empirical evidence for NK-cell infiltration in DPN remains limited, NK-cell participation in neuroimmune interactions has been observed in certain models of peripheral nerve injury and neuroinflammation. NK cells can participate in local inflammatory responses by recognizing stress ligands expressed on the surface of injured or stressed cells, suggesting that, in the inflammatory background of DPN, NK cells may also participate in regulation of the neural immune microenvironment.

#### 3.4.2 NK cell-mediated neural injury and protective effects

NK cells possess cytotoxic activity and exert direct killing of target cells while also participating in immune regulation through the release of perforin, granzymes, and IFN-γ. Perforin forms pores in the membrane of target cells, allowing granzymes to enter the cell and initiate apoptosis, thereby mediating cytotoxic effects (159). This killing mechanism is essential in host defense against infection and tumors, and under conditions of neural injury or stress it may also act on damaged cells within neural tissue. Studies suggest that, under neuropathological conditions such as chronic neuropathic pain, NK-cell numbers are statistically associated with pain-related indicators. In a study of patients with chronic peripheral neuropathy and postherpetic neuralgia, cerebrospinal fluid immunocyte analysis showed that NK-cell frequency was negatively correlated with mechanical pain sensitivity (MPS), meaning that a higher NK-cell frequency was associated with lower mechanical pain sensitivity. This finding suggests that NK cells may exert a certain protective effect against neuroinflammatory pain by suppressing central sensitization processes or modulating the inflammatory microenvironment (160).

Under chronic metabolic and inflammatory conditions such as DPN, NK cells may possess both potentially protective immunoregulatory functions and, when abnormally activated, the capacity to directly damage neural tissue. These bidirectional effects underscore the complexity of NK cells in neuroimmune interactions and suggest that, in the pathological process of DPN, they may act either as protective factors or as participants in inflammatory injury.

### 3.5 Mast cells

Mast cells are tissue-resident immune effector cells whose activation results in release of mediators such as histamine, proteases, and cytokines. Under hyperglycemic conditions, mast cells not only undergo immunometabolic activation, but also aggravate inflammation in the neural microenvironment by altering mitochondrial function and metabolic pathways. In peripheral nerve tissue from patients with DPN, both the number and activity of mast cells are significantly increased, promoting release of inflammatory factors such as TNF-α and IL-1β and correlating with degenerative changes in neural axons (161). Using single-cell transcriptomics and multiple complementary techniques, Yao et al. systematically revealed the key pathogenic role of mast cells in DPN. Under hyperglycemic conditions, mast cells become aberrantly activated and, through degranulation-mediated release of inflammatory mediators together with induction of mitochondrial dysfunction and endoplasmic reticulum stress, synergistically drive neural demyelination and axonal degeneration. Activated mast cells participate in degenerative changes and inflammatory responses during DPN progression (161), thereby providing new cellular targets and potential intervention strategies for the prevention and treatment of DPN.

### 3.6 Dendritic cells

Dendritic cells (DCs) are the most potent antigen-presenting cells. Long-term hyperglycemia leads to accumulation of damage-associated molecular patterns in neural tissue. After sensing these danger signals, resident DCs become activated, mature, and migrate to draining lymph nodes, where they present captured neural antigens to naïve T cells and promote their differentiation into pro-inflammatory Th1/Th17 subsets. At the same time, activated DCs recruit macrophages and other immune cells to infiltrate the endoneurium by secreting chemokines and interact locally with T cells, thereby further amplifying inflammatory responses. This process directly leads to neuronal and Schwann-cell injury, endoneurial microangiopathy, and ischemic injury, ultimately causing peripheral nerve dysfunction (162, 163).

### 3.7 Neutrophils

Neutrophils are the principal effector cells of acute inflammation. Under diabetic conditions, their lifespan is prolonged and they readily form neutrophil extracellular traps (NETs), which consist of a DNA scaffold, histones, and granule proteins. NETosis is a form of hyperglycemia-induced programmed death in neutrophils and, through NET release, plays a central regulatory role in the pathogenesis of diabetes and its complications, including cardiovascular disease, nephropathy, retinopathy, and impaired wound healing (164). Neutrophils are also the most abundant phagocytic cells in the body and are rapidly recruited and activated during acute inflammation. In patients with DPN, an elevated neutrophil-to-lymphocyte ratio (NLR) suggests that chronic low-grade inflammatory status is related to lesion progression (165).

### 3.8 Basophils and eosinophils

Eosinophils and basophils are two typical granulocyte subsets that play important effector roles in humoral immune responses such as allergic reactions and parasitic infections. However, under chronic inflammatory conditions and states of immunometabolic dysregulation, they may also participate in peripheral neuropathological processes, including diabetic peripheral neuropathy.

Although basophils are few in number in peripheral blood, their function cannot be ignored as an important component of immune responses. Basophils can secrete Th2-type cytokines such as IL-4 and IL-13 and promote migration of other immune cells to inflammatory sites by inducing vascular endothelial cells to express adhesion molecules and chemokines (166); This network of immune mediators may exert sustained effects on the tissue microenvironment under chronic inflammatory conditions.

Eosinophils are granular leukocytes that differentiate and mature under regulation by cytokines such as IL-5 and IL-3. Their cytoplasm contains multiple cytotoxic granule proteins, including major basic protein (MBP), eosinophil cationic protein (ECP), and eosinophil-derived neurotoxin (EDN). Hypereosinophilic syndrome (HES) is clearly associated with peripheral neuropathy. Case reports have shown that patients with HES can present with sensory and motor nerve injury, and eosinophil infiltration together with neural tissue injury has been observed in peripheral nerve biopsies, suggesting that eosinophils may participate in neuropathological injury through tissue infiltration and release of cytotoxic mediators (167). Eosinophilic granulomatosis with polyangiitis (EGPA), a disease characterized by eosinophil infiltration and necrotizing vasculitis, commonly involves peripheral neuropathy in clinical practice, and its pathological features suggest that eosinophil-related vascular occlusion and tissue injury mechanisms participate in peripheral neuropathology. In a clinical study, anti-IL-5 monoclonal antibodies such as mepolizumab improved symptoms of peripheral neuropathy in patients with EGPA, indicating that lowering eosinophil levels can alleviate pain and sensory abnormalities related to neural injury. This further underscores the importance of eosinophils to neural health under specific inflammatory conditions (168).

### 3.9 Microglia

Microglia are resident immune cells of the central nervous system and possess both macrophage-like phagocytic and immune-surveillance functions (169). under normal physiological conditions, microglia remain in a homeostatic surveillance state and maintain microenvironmental homeostasis in the central nervous system. Through TGF-β1, they regulate the growth of existing myelin, preserve myelin integrity, and at the same time prevent demyelination and abnormal myelin hyperplasia. When the neural environment changes, microglia become activated and initiate inflammatory responses.

In the diabetic setting, persistent hyperglycemia induces metabolic imbalance, oxidative stress, and peripheral nerve injury, all of which together enhance microglial reactivity in the spinal dorsal horn. Increased numbers of activated microglia have been observed in experimental diabetic models, and these cells participate in DPN development (170). Hyperglycemia drives microglia toward a pro-inflammatory reactive state and is accompanied by increased production of inflammatory mediators (171). In a hyperglycemia-induced inflammatory environment, extracellular ATP levels increase (172). Binding of ATP to P2X4R on microglia activates the intracellular inflammatory complex NLRP3, which assembles into an inflammasome complex and promotes production of inflammatory factors such as IL-1β and IL-18 (172). IL-18 is released specifically by spinal microglia and promotes chronic pain development by activating NF-κB signaling in astrocytes through IL-18R (173). Compared with nondiabetic controls, patients with type 2 diabetes show significantly elevated serum IL-18 levels together with upregulated IL-18R expression (174). In nerve injury models, activated microglia promote tactile allodynia through P2X4R activation. At the same time, microglia act on astrocytes through the IL-18/IL-18R pathway and promote NF-κB phosphorylation in astrocytes, thereby also participating in the induction of tactile allodynia (173, 175). Hyperglycemia also causes mitochondria to generate large amounts of superoxide and increases flux through the hexosamine and PKC pathways, thereby increasing ROS production in microglia (176). Excessive ROS in microglia can further enhance NLRP3 activation (177), thereby promoting maturation and release of inflammatory mediators.

Hyperglycemia enhances aerobic glycolysis in microglia by inducing downregulation of sirtuin 3 (Sirt3) (178), thereby promoting microglial proliferation, activation, and inflammatory cytokine secretion. Studies have shown that Sirt3 expression is downregulated in the spinal dorsal horn of DPN mice. Further experiments demonstrated that Sirt3 deficiency aggravates hyperglycemia-induced neuroinflammation and diabetic neuropathic pain by enhancing aerobic glycolysis in microglia, whereas microglial overexpression of Sirt3 alleviates inflammation by reducing aerobic glycolysis. Mechanistically, high glucose activates protein kinase B (Akt), causing phosphorylation-mediated inactivation of FoxO1 and thereby reducing the transcriptional capacity of Sirt3; at the same time, hyperglycemia induces Sirt3 degradation through the mitophagy–lysosomal pathway. In addition, Sirt3 expression is decreased in DRG neurons of rats with painful DPN, whereas induced overexpression of Sirt3 ameliorates painful DPN by activating FoxO3a–PINK1–Parkin-mediated mitophagy (179), indicating that Sirt3 may be a potentially effective therapeutic target in DPN.

After peripheral nerve injury, inflammatory factors derived from the periphery can also bind to TLRs on microglia. TLR4 induces recruitment of myeloid differentiation primary response 88 (MyD88) and activates NF-κB transcription in neurons and glial cells, resulting in production of multiple cytokines and promoting the induction and persistence of pain (180, 181). Hyperglycemia also prolongs LPS–TLR-binding-induced NF-κB activation, thereby aggravating inflammatory responses (182). Together, these mechanisms promote microglial participation in central sensitization, that is, heightened responsiveness of nociceptive neurons in the central nervous system to incoming signals, ultimately leading to hypersensitivity, generalization, and persistence of neuropathic pain.

**Figure 1:**
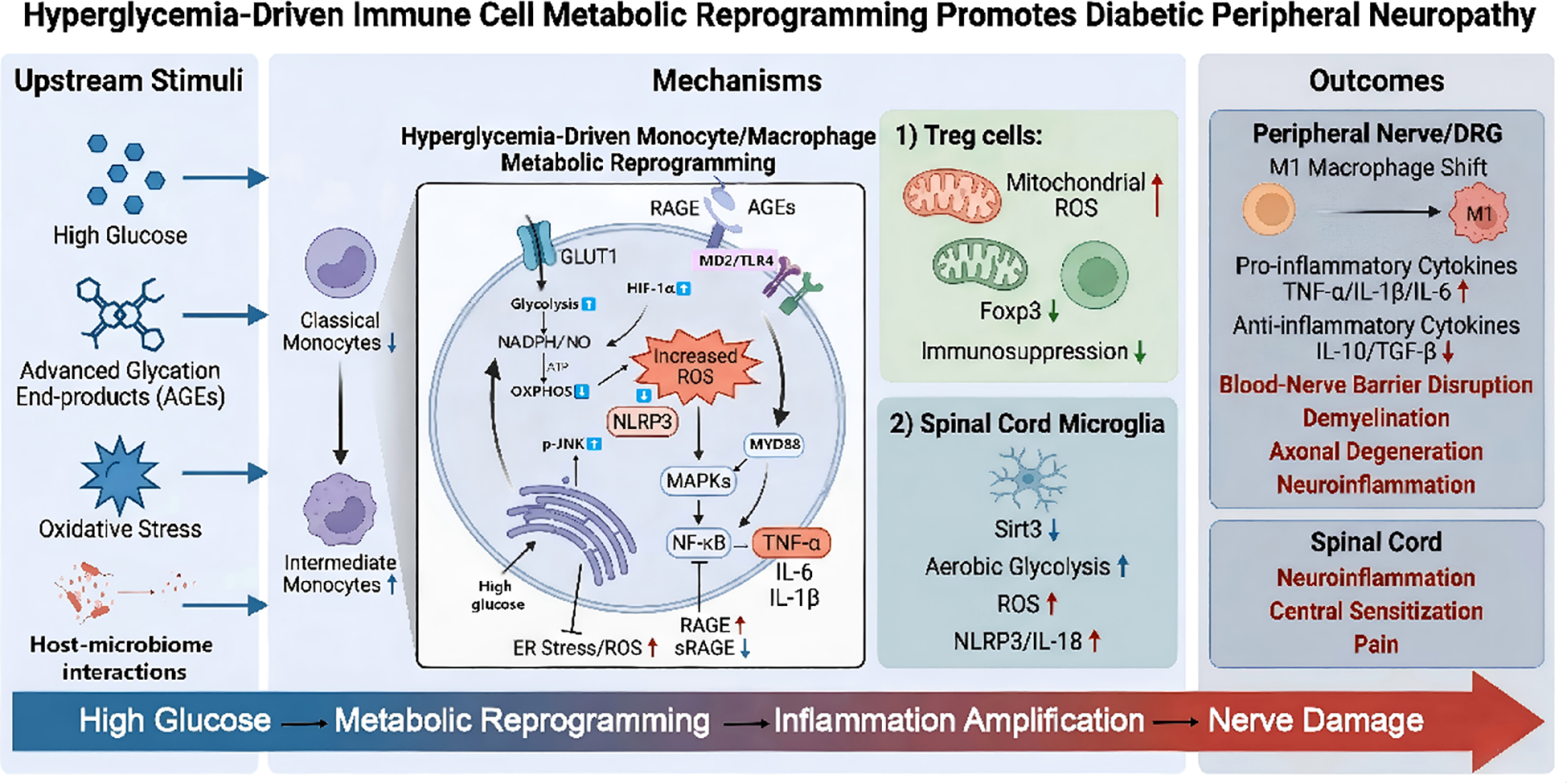
Hyperglycemia-Driven Immune Cell Metabolic Reprogramming Promotes Diabetic Peripheral Neuropathy.

**Figure 2:**
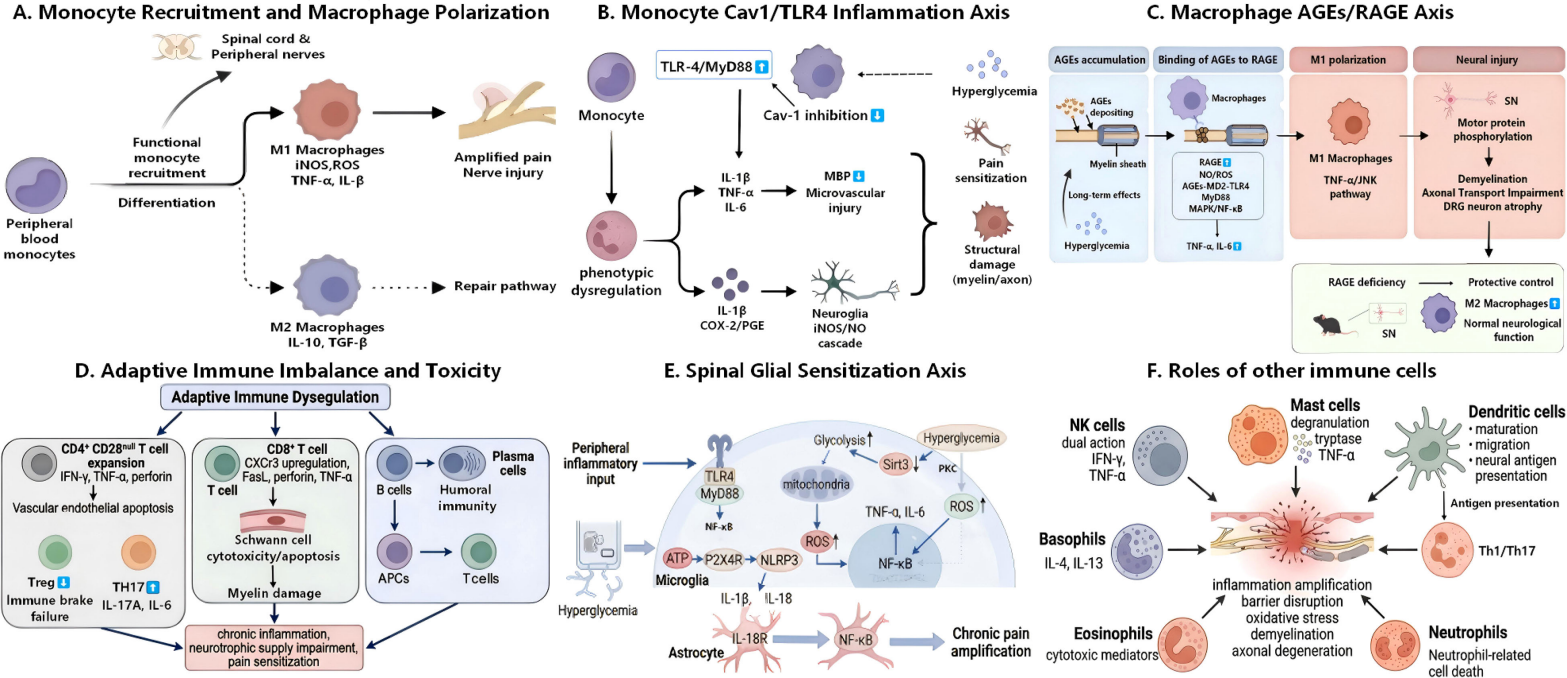
Roles of immune cells in the pathogenesis of DPN. **(A)** Monocyte recruitment and macrophage polarization; **(B)** Monocyte Cav1/TLR4 inflammation axis; **(C)** Macrophage AGEs/RAGE axis; **(D)** Adaptive immune imbalance and toxicity; **(E)** Spinal glial sensitization axis; **(F)** Roles of other immune cells.

Immune cells contribute to DPN progression through a complex interaction network (**Figures 1**, **2**; **Table 1**). As transitional cells linking blood and tissue immunity, monocytes infiltrate the spinal cord and sciatic nerve under diabetic conditions and differentiate into macrophages; this process is related to the MCP-1/CCR2 axis, and depletion of peripheral monocytes can concomitantly reduce IL-1β and TNF-α levels and reverse mechanical allodynia. Meanwhile, hyperglycemia induces immunometabolic reprogramming in monocytes/macrophages: enhanced glycolysis, reduced oxidative phosphorylation (OXPHOS), ROS accumulation, and endoplasmic reticulum stress together amplify pro-inflammatory signaling such as p-JNK, MAPK/NF-κB, and NLRP3 and link oxidative stress to demyelination through the AGEs–RAGE pathway. Expansion of pro-inflammatory CD4^+^CD28null T cells together with reduced number and impaired function of Treg cells jointly promote the chronic inflammatory state of DPN, whereas hyperglycemia drives CD8^+^ T-cell infiltration through the CXCL9/10/11–CXCR3 axis and mediates Schwann-cell cytotoxicity, thereby directly linking immune attack to destruction of myelin and axonal structure. Through autoantibody production, antigen presentation, and cytokine secretion, B cells promote immune-mediated injury in neural tissue.

**Table 1:** Summary of the roles of immune cells in DPN.

Immune cell	Major changes in DPN	Key molecules/pathways	Impact on nerve injury
Monocytes/Macrophages	Increased M1 polarization, insufficient M2 response, increased monocyte recruitment/infiltration	TLR4/NF-κB, TNF-α, IL-1β, NLRP3, MCP-1/CCR2, AGEs-RAGE, MyD88	Promotes inflammation, myelin damage, abnormal neural blood flow, mechanical pain hypersensitivity, and impaired nerve regeneration
T cells	Th1/Th17 shift, insufficient Treg cells, increased CD8+ T-cell infiltration	IFN-γ, IL-17, IL-10, Foxp3, CXCL9/CXCL10/CXCL11-CXCR3	Amplifies chronic inflammation, contributes to pain sensitization, damages Schwann cells, and promotes demyelination and axonal injury
B cells	Abnormal antibody and cytokine secretion, defective Breg function, enhanced antigen presentation	IL-6, TNF-α, anti-ganglioside antibodies, IL-10, IL-35, TGF-β	May participate in amplification of inflammation and drive autoimmune injury to neural tissue
NK cells	Abnormal cytotoxicity and inflammatory regulation, increased proportion of mature NK cells	IFN-γ, perforin/granzyme, TNF-α, ADCC	May participate in neuroinflammation and target-cell apoptosis, and aggravate nerve injury when aberrantly activated
Mast cells	Degranulation activation, increased number and activity	Histamine, TNF-α, tryptase, IL-1β	Causes neural sensitization, amplifies inflammation, disrupts the blood-nerve barrier, and induces axonal degeneration
Dendritic cells	Enhanced antigen presentation, increased activation/maturation and migration	T-cell activation, Th1/Th17	Promotes Th1/Th17 responses, amplifies neuroinflammation, and damages neurons/Schwann cells
Neutrophils	Early inflammatory recruitment, prolonged lifespan, increased NET formation	ROS, proteases, NETs	Aggravates oxidative stress and tissue injury, and promotes endothelial injury
Eosinophils	Limited evidence; may infiltrate and participate in peripheral nerve injury	MBP, ECP, EDN, IL-5	May participate in tissue inflammation and nerve injury
Basophils	Limited evidence	IL-4, IL-13	May participate in inflammatory networks and endothelial dysfunction
Microglia/Satellite glial cells	Enhanced activation, increased number, and polarization toward the M1 phenotype	p38 MAPK, NF-κB, TLR4/MyD88, NLRP3, IL-1β, IL-18	Promote pain sensitization in the DRG and spinal cord, central sensitization, and maintenance of chronic pain

NK cells may also aggravate neuroinflammation and functional impairment through cytotoxicity, release of pro-inflammatory factors, and direct interactions with neurons. B cells and NK cells may act synergistically: immune complexes produced by B cells can activate complement and release chemotactic factors such as C5a, thereby attracting NK cells to neural tissue; meanwhile, IFN-γ secreted by NK cells may promote differentiation of B cells into plasma cells, forming a positive-feedback loop. Mast cells promote neuroinflammation and blood–nerve barrier disruption by releasing histamine, tryptase, and TNF-α through degranulation. Dendritic cells mature and migrate to the DRG under hyperglycemic conditions and activate T-cell-mediated autoimmune responses, whereas neutrophils aggravate oxidative stress and endothelial injury by forming NETs. Basophils can regulate Th2 immune skewing by secreting IL-4 and histamine and promote endothelial dysfunction under hyperglycemic conditions; eosinophils may participate in tissue inflammation and neural injury by releasing major basic protein (MBP), eosinophil cationic protein (ECP), and IL-5. Peripheral inflammation is transmitted centrally through pathways such as TLR4–MyD88–NF-κB; under hyperglycemic conditions, microglia increase in number and shift toward a pro-inflammatory phenotype, and activated microglia together with astrocytes promote central sensitization and participate in the formation of chronic pain in DPN. Collectively, these immune cells and their related pathways may provide new strategies for the treatment of DPN.

## 4 Cell–cell crosstalk networks in DPN

**Figure 3:**
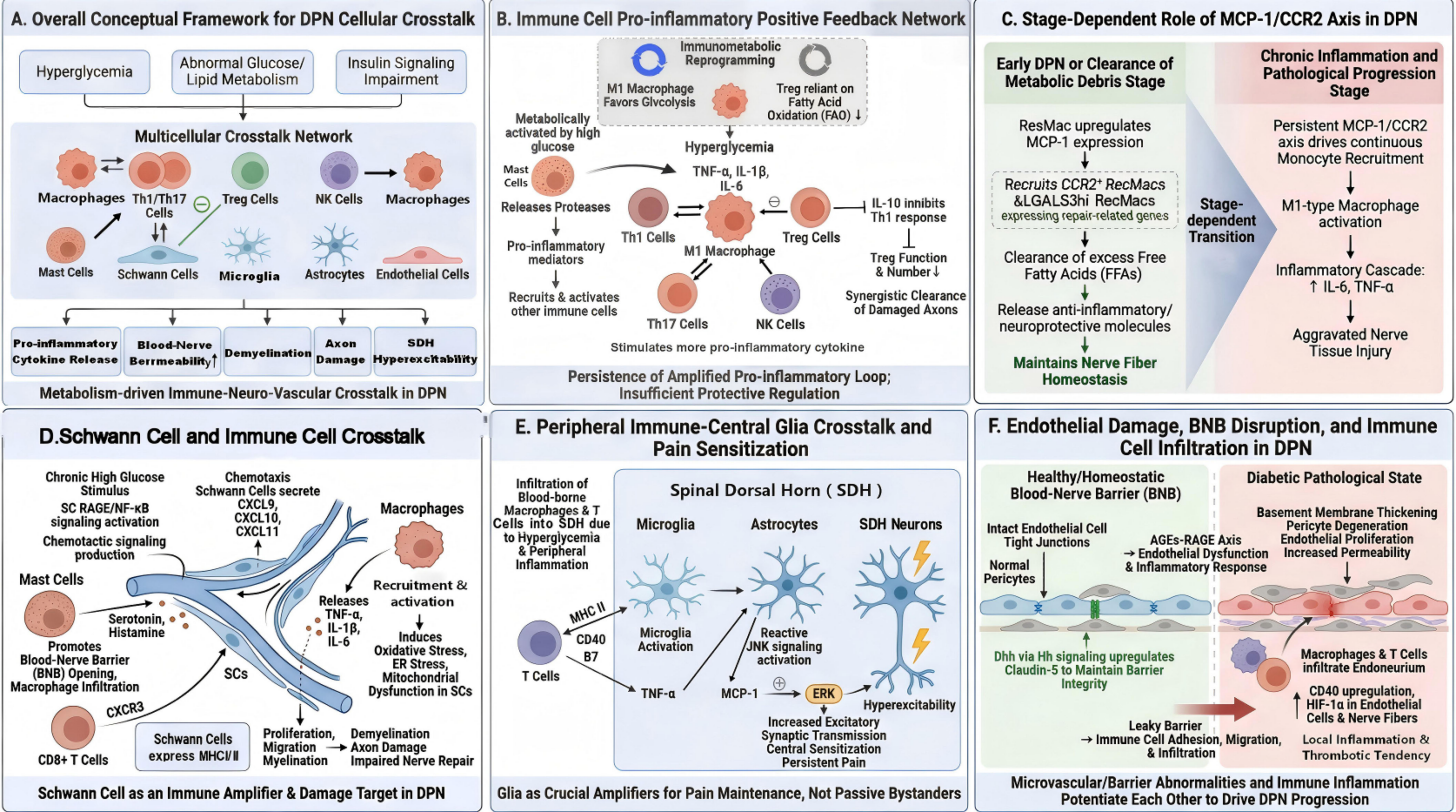
Cell–Cell Crosstalk Metabolic–neural–immune Networks in DPN. **(A)** Overall Conceptual Framework for DPN Cellular Crosstalk; **(B)** Immune Cell Pro-inflammatory Positive Feedback Network; **(C)** Stage-Dependent Role of MCP-1/CCR2 Axis in DPN; **(D)** Schwann Cell and Immune Cell Crosstalk; **(E)** Peripheral Immune-Central Glia Crosstalk and Pain Sensitization; **(F)** Endothelial Damage, BNB Disruption, and Immune Cell Infiltration in DPN.

The occurrence and progression of diabetic neuropathy are complex processes in which interactions and crosstalk mechanisms among immune cells play key roles. These mechanisms involve not only the activation and reprogramming of immune cells themselves, but also multidirectional interactions between immune cells and Schwann cells and other cell types within the peripheral nervous system (7), together forming a complex metabolic–neural–immune network under conditions of metabolic dysregulation such as hyperglycemia (**Figure 3**).

### 4.1 Crosstalk among immune cells

Under hyperglycemic conditions, immune cells become activated and undergo extensive metabolic reprogramming involving glycolysis, oxidative phosphorylation, and metabolite synthesis, and this constitutes the basis of immune-cell crosstalk (183). This process is termed immunometabolism. It not only provides energy for immune cells and regulates their functions, but changes in intracellular metabolic pathways also directly affect the homeostasis, proliferation, and differentiation of innate and adaptive immune-cell subsets (24).

Glycolysis is a key metabolic pathway by which activated immune cells meet anabolic demands. After LPS stimulation, M1 macrophages undergo the Warburg effect (184), preferentially performing glycolysis even under aerobic conditions to produce ATP and biosynthetic precursors, thereby promoting secretion of inflammatory cytokines. In T cells, increased glycolysis is observed after activation in multiple subsets, such as helper T cell 1 (Th1) (185), Th17 (186), and effector CD8+ T cells (187), to support their rapid proliferation, clonal expansion, and production of effector cytokines. In addition, natural killer cells (188) and B lymphocytes (189) likewise depend on glycolysis after activation to support their cytotoxic functions and antibody production.

Fatty-acid oxidation plays a key role in regulating adaptive and innate immune responses and is associated with anti-inflammatory, tolerant, and long-lived immune-cell phenotypes. In macrophages, abnormal accumulation of fatty acids and their derived lipoproteins promotes foam-cell formation, whereas enhancement of fatty-acid oxidation can alleviate macrophage-mediated inflammatory responses (190). Regulatory T cells depend on fatty-acid oxidation, and this metabolic pathway helps create a metabolic microenvironment that suppresses effector T-cell function and promotes immune tolerance (191). In addition, CD8+ T cells also depend on fatty-acid oxidation during memory formation to maintain their long-term survival and rapid recall responses (192).

Fatty-acid synthesis is associated with the proinflammatory, proliferative, and effector functions of immune cells. Inflammatory signaling upregulates fatty acid synthase through the SREBP transcription factor to promote lipogenesis, a process that is crucial for membrane synthesis, endoplasmic reticulum expansion, and inflammasome activation in M1 macrophages (193). After activation, dendritic cells enhance glycolysis (194) and fatty-acid synthesis (195), thereby supporting their antigen-presenting function and activation of CD8+ T cells. Fatty-acid synthesis is likewise crucial for Th17-cell differentiation (196), and polyunsaturated fatty acids synthesized under the guidance of CD5 antigen-like protein (CD5L) promote Th17 cells to produce the anti-inflammatory cytokine IL-10 (197). Activated effector T cells (198) and B cells (199) require large-scale synthesis of new fatty acids and sterols to build cell membranes and signaling molecules for proliferation.

In neural tissue from patients with DPN, infiltration of macrophages, T cells, and NK cells is present, with macrophage accumulation being particularly prominent and distinct from the response to acute injury (161). The sensory abnormalities and persistent pain of chronic DPN are accompanied by infiltration of T cells and neutrophils into the DRG (49). After nerve injury, T cells infiltrate the spinal dorsal horn, where IFN-γ-expressing Th1 cells are significantly upregulated and directly participate in and drive the evolution of pain-like hypersensitivity (200). Hyperglycemic stimulation promotes macrophage polarization toward an M1 phenotype characterized predominantly by glycolysis (103). The inflammatory cytokines secreted by these macrophages, such as TNF-α, IL-1β, and IL-6 (201), not only directly injure neurons and Schwann cells, but also act as key signaling molecules driving the differentiation and activation of Th1 and Th17 cells (202, 203). Meanwhile, IFN-γ and IL-17 produced by Th1/Th17 cells further stimulate the production of inflammatory factors such as TNF-α, IL-1β, and IL-6 and in turn induce chemokine-mediated recruitment of macrophages, forming a positive-feedback amplification loop that aggravates neuroinflammatory responses (204). Mast cells also exert an important amplifying effect in the immune microenvironment of DPN. Under hyperglycemic stimulation, mast cells become metabolically activated through the ERK1/2–mTOR signaling pathway and release proteases and proinflammatory mediators (161), thereby acting upstream to recruit and activate macrophages, T cells, and other immune cells and to initiate and amplify the local inflammatory cascade.

Studies have shown that the proportion of mature NK cells is increased in patients with diabetic neuropathic pain and that these cells can induce apoptosis of target cells through antibody-dependent cellular cytotoxicity (ADCC), suggesting their potential role in immune responses related to neural injury (205). Recruitment of NK cells to sites of injured nerves may participate synergistically with macrophages in the process of Wallerian degeneration, selectively clearing damaged axons and thereby limiting secondary inflammatory responses and alleviating subsequent pain hypersensitivity (206). However, the specific impact of NK cell–macrophage cooperation on pain outcomes remains unclear (205). Treg cells play protective roles in pain caused by neuropathy by suppressing the Th1 response of CD4+ T cells through secretion of IL-10 (207), and can reduce infiltration of T cells, macrophages, and antigen-presenting cells in ganglia (208). In DPN, however, Treg function and numbers are suppressed, leading to diminished counterbalancing capacity against proinflammatory immune crosstalk and allowing inflammatory responses to remain chronically dominant. These studies reveal the important roles of immune-cell infiltration and inflammation in DPN development.

MCP-1 is a monomeric polypeptide secreted by monocytes, macrophages, and dendritic cells at sites of infection, injury, and tissue damage (209). A study including 102 patients with T2DM showed that patients with confirmed DPN had higher MCP-1 levels than patients without DPN, and that IL-8 and MCP-1 were simultaneously increased in patients with DPN; elevated MCP-1 levels were also associated with other inflammatory cytokines such as IL-6 and TNF-α. All of these findings indicate the presence of an inflammatory cascade in patients with DPN and further suggest the involvement of MCP-1 in these inflammatory pathways (115). During inflammation, MCP-1 secretion is induced by proinflammatory mediators such as TNF-α, IFN-γ, IL-1β, and platelet-derived growth factor (PDGF). By promoting monocyte recruitment, monocyte infiltration, and inflammation, and by having its effects amplified by obesity and other inflammatory factors, MCP-1 may participate in neural injury in diabetic neuropathy (102). Animal experiments showed that MCP-1 levels were elevated in the sciatic nerves of DPN rats, and paeonol treatment significantly reduced MCP-1 levels, improved neural function, and prevented neural injury (54). Another experiment showed that inhibition of the MCP-1/CCR2 pathway significantly reduced expression of M1 macrophage markers and secretion of proinflammatory factors, thereby improving the inflammatory status in diabetic nephropathy, suggesting that similar mechanisms may also be effective in peripheral neuropathy (210).

However, macrophages recruited through the MCP-1/CCR2 pathway exert neuroprotective effects in early DPN. In the sciatic nerves of mice fed a high-fat high-fructose diet for 12 weeks, ResMacs upregulate MCP-1 expression, leading to increases in CCR2+ RecMacs and LGALS3hi recMacs, which express repair-promoting genes. These phagocytes may reduce toxic injury by clearing metabolic debris in the neural microenvironment, such as excess free fatty acids, while also secreting anti-inflammatory and neuroprotective molecules, suppressing nociceptor sensitization, and promoting maintenance of nerve-fiber homeostasis (129). By contrast, macrophages differentiated from peripheral blood monocytes of patients with painful diabetic neuropathy (PDN) show reduced production of the proinflammatory chemokine MCP-1 and the anti-inflammatory cytokine IL-10show dysfunctional reductions in production of the proinflammatory chemokine MCP-1 and the anti-inflammatory cytokine IL-10 (102), which may be related to long-term exposure to a hyperglycemic microenvironment.

MCP-1 also participates in the development and progression of neuropathic pain. In spinal astrocytes, TNF-α activates JNK and stimulates upregulation of MCP-1, thereby inducing phosphorylation of extracellular signal-regulated kinase in spinal dorsal horn neurons, enhancing excitatory synaptic transmission, and promoting central sensitization and neuropathic pain; furthermore, intrathecal administration of an MCP-1-neutralizing antibody can alleviate neuropathic painurther intrathecal administration of an MCP-1-neutralizing antibody can alleviate neuropathic pain. Experiments have also found that the MCP-1 receptor CCR2 is expressed in spinal neurons and some non-neuronal cells, suggesting that the MCP-1/CCR2 pathway participates in neuronal pain sensitization (211). In addition, MCP-1 can activate wild-type spinal microglia, but not microglia from CCR2-knockout mice. Another experiment found that MCP-1 can promote neuropathic pain and motor dysfunction in DPN mice through CCR4 (212). Clinical trials have shown that deep tissue laser therapy significantly reduces pain in patients with diabetic peripheral neuropathy by lowering serum IL-6 and MCP-1 levels (213).

### 4.2 Crosstalk between immune cells and non-immune cells

The role of immune cells in the pathogenesis of DPN is far from isolated. In addition to interacting with one another, immune cells form complex crosstalk networks with non-immune cells such as neurons and endothelial cells, and these networks are a core mechanism jointly driving the onset and progression of DPN. Abnormal glucose and lipid metabolism and impaired insulin signaling not only directly induce a series of pathological changes in neurons and glial cells, including DNA damage (214), endoplasmic reticulum stress (215), and mitochondrial dysfunction (25), thereby causing neural dysfunction, but also activate inflammatory signaling pathways related to non-immune cells, which in turn recruit and amplify immune-cell activation and infiltration and ultimately aggravate neural injury (216).

#### 4.2.1 Immune–neural cell crosstalk

Schwann cells, as the principal glial cells of the peripheral nervous system, play key roles in maintaining the structural and functional integrity of both unmyelinated and myelinated axons (217). Schwann cells are involved in activation of innate immune responses; after nerve injury, they can recruit macrophages to sites of nerve injury by secreting chemokines and cytokines, regulate macrophage activation states, and modulate myelin phagocytosis (218, 219). Opening of the blood–nerve barrier triggered by serotonin, histamine, and other substances released by resident mast cells promotes macrophage infiltration (220). Schwann cells can also initiate adaptive immune functions by expressing major histocompatibility complex (MHC) class I and II molecules and activating T cells in peripheral nerves (220, 221). Under long-term hyperglycemic conditions, pathological changes such as abnormal axon–Schwann cell metabolic transport, dysregulated protein expression in the DRG, demyelination, and axonal degeneration can be triggered (216). High glucose levels upregulate expression of the chemokine receptor CXCR3 on CD8+ T cells through activation of the MAPK signaling pathway and simultaneously induce expression of CXCL9, CXCL10, and CXCL11 in Schwann cells, thereby establishing a specific chemotactic axis that guides CD8+ T cells to infiltrate peripheral nerve tissue and exert cytotoxic effects that damage Schwann cells (149). Persistent hyperglycemic stimulation can activate the RAGE/NF-κB signaling pathway in Schwann cells and recruit and activate immune cells such as macrophages (222). After accumulating in peripheral nerves, these cells release proinflammatory cytokines that can trigger apoptosis, pyroptosis, and other lytic cell-death pathways. The proinflammatory cytokines released by these cells after accumulating in peripheral nerves can trigger activation of apoptosis, pyroptosis, and pan-cell-death pathways in Schwann cells by inducing oxidative stress, endoplasmic reticulum stress, and mitochondrial dysfunction (222), thereby inhibiting their proliferation, migration, and myelin-forming capacity, leading to demyelination and axonal injury, and hindering the process of nerve repair (201).

Glial cells in both the central and peripheral nervous systems jointly mediate neuroinflammatory signal transmission and neuronal hyperexcitability in DPN (223). Bidirectional communication between astrocytes and microglia plays a central role in the regulation of central neuroinflammation, and both cell types mediate neuroinflammatory responses through release of multiple cytokines and inflammatory mediators. For example, LPS-activated microglia can induce astrocytes to shift toward a neurotoxic reactive phenotype (224). Activated astrocytes participate in regulation of pain.

Activated astrocytes regulate pain signaling by secreting inflammatory mediators and modulating synaptic activity and neurotransmitter homeostasis signaling pathways by secreting inflammatory factors and modulating synapses and neurotransmitter homeostasis, and they are key drivers in the occurrence and maintenance of diabetic neuropathic pain (225). In addition to crosstalk among glial cells, peripheral immune cells also aggravate neuroinflammatory responses through interactions with central glial cells. T cells may interact with microglia that have local antigen-presenting capacity through co-stimulatory molecules such as MHC II, CD40, and B7, thereby inducing activation of astrocytes in the spinal dorsal horn and exacerbating neuropathic pain (226). Spinal microglia are activated and undergo morphological remodeling under hyperglycemic conditions (227), synthesize and release proinflammatory cytokines and neuroactive molecules, and thereby cause hyperexcitability of spinal nociceptive neurons (228). Under hyperglycemic conditions, blood-derived monocyte/macrophages become activated and infiltrate spinal tissue.Blood monocyte-derived macrophages are activated and infiltrate spinal tissue under the drive of hyperglycemia, and the immune responses in which they participate may further promote the development of painful diabetic neuropathy (56).

#### 4.2.2 Immune–endothelial cell crosstalk

In DPN, in addition to injury of peripheral nerve axons and dysfunction of Schwann cells, peripheral nerve homeostasis is also disrupted by breakdown of blood–nerve barrier (BNB) integrity the homeostatic mechanisms of peripheral nerves are also affected by disruption of blood–nerve barrier (BNB) homeostasis, ultimately leading to neuronal dysfunction and promoting neuropathy progression (10, 229). BNB dysfunction is manifested as increased permeability, and its structural abnormalities typically include basement membrane thickening, pericyte degeneration, and endothelial hyperplasia its structural abnormalities include common basement membrane thickening, pericyte degeneration, and endothelial-cell hyperplasia (230). Impaired endothelial-cell function not only disrupts neural microvascular homeostasis, but also creates favorable conditions for the recruitment, adhesion, and infiltration of peripheral immune cells by weakening barrier integrity, thereby amplifying neuroinflammatory responses. Desert hedgehog (Dhh) maintains BNB integrity through hedgehog (Hh) signaling in endothelial cells and upregulates expression of the tight-junction protein claudin-5 (Cldn5), thereby reducing vascular permeability (231). A sural nerve biopsy study in diabetic patients further revealed the close association between microangiopathy and immune responses (83). In the damaged microvascular environment, macrophages and T cells markedly infiltrate the endoneurium, accompanied by upregulated expression of surface CD40 molecules, which induces increased expression of HIF-1α in endothelial cells and nerve fibers and thereby aggravates local inflammation and thrombosis (83). At the same time, RAGE is upregulated in peripheral nerve endothelial cells and Schwann cells of patients with diabetic neuropathy (232). Through the RAGE signaling pathway, AGEs cause endothelial dysfunction and inflammatory responses and thereby further promote the development of diabetic neuropathy (233). Under neuropathic pain conditions, the chemokines CCL2 and CX3CL1 enhance permeability of the blood–central nervous system barrier by promoting expression of adhesion molecules on endothelial cells and guide migration of peripheral monocytes and T cells into the central nervous system (234). After entering the central nervous system, these immune cells interact with activated central immune cells such as microglia through immune synapses (234), promoting the persistence and spread of inflammatory responses and ultimately leading to abnormal neuronal activity.

## 5 Therapeutic strategies targeting immunometabolic mechanisms

At present, clinical interventions for diabetic neuropathy (DN) focus mainly on glycemic control, lifestyle intervention, and management of neuropathic pain (235), among which good glycemic control remains the cornerstone for delaying disease progression. Current clinical guidelines recommend a variety of pharmacological regimens for painful diabetic peripheral neuropathy (PDPN), mainly including calcium channel α2δ ligands (such as gabapentin and pregabalin), selective serotonin and norepinephrine reuptake inhibitors (such as duloxetine), and tricyclic antidepressants (such as amitriptyline, nortriptyline, and desipramine) (236), which can alleviate neural dysfunction and neuropathic pain to some extent (237). However, existing oral medications have problems in clinical application, including limited overall efficacy, an unfavorable risk–benefit ratio, poor patient adherence, and high discontinuation rates (238). In addition, although opioids have analgesic effects, the risks of addiction and abuse associated with long-term use mean that guidelines no longer recommend them as routine therapy (239). With deeper understanding of the pathogenesis of DN, immunometabolic regulation has gradually become an important research direction. Metabolic processes not only provide cells with energy and biosynthetic precursors, but also regulate immune and inflammatory responses. Therefore, therapeutic strategies targeting immunometabolic mechanisms show great promise(**Table 2**).

**Table 2:** Therapeutic strategies for DN and mechanisms.

Therapeutic strategy	Main mechanisms	Effects on nerve injury
Classical glucose-lowering agents		
Metformin	Reprograms T-cell metabolism, inhibits macrophage FASN, suppresses mitochondrial complex I, reduces ROS, and rebalances IL-1β/IL-10	Suppresses immune inflammation and indirectly confers neuroprotection
SGLT2 inhibitors	Reduce Iba1 and IL-6 expression; inhibit macrophage and microglial activation	Attenuate neuroinflammation
GLP-1 receptor agonists	Activate cAMP/PKA, inhibit iNOS, promote DRG neurite outgrowth, and reduce IL-6	Inhibit neuroinflammation, promote neurite growth, and protect nerves
Antioxidant/polyol-pathway inhibition		
Benfotiamine	Inhibits the polyol and hexosamine pathways and AGEs formation; reduces aldose reductase activity; suppresses pro-oxidative microglial activity	Improves DN-related symptoms
α-Lipoic acid	Regenerates endogenous antioxidants, promotes glucose uptake, and exerts anti-inflammatory effects	Improves sensory symptoms and relieves pain
Aldose reductase inhibitors	Inhibit the polyol pathway	Delay decline in motor nerve conduction and vibration perception; improve motor nerve function
PKC inhibitors	Reduce p-PKCβ in DRG, restore cGMP, and regulate the nNOS-cGMP axis	Improve hyperalgesia and possibly overall symptom scores
Support of energy metabolism		
Acetyl-L-carnitine	Improves mitochondrial energy metabolism	Improves neuropathic symptoms and neurophysiology, though evidence remains inconsistent
Neurotrophic factors		
Neurotrophic factors	NGF activates PI3K/Akt to inhibit ER stress-induced apoptosis; BDNF regulates neuronal excitability; MANF inhibits NF-κB/p38 MAPK and reduces macrophage infiltration	Improve neurodegeneration/demyelination, relieve pain, and improve mechanical hypersensitivity and nerve conduction
Cell/gene/molecular-targeted therapies		
Cell therapy	Intravenous/intrathecal delivery; inhibits TLR2/NF-κB, regulates macrophage M1/M2 polarization, and releases neurotrophic factors/exosomes	Reduces neuronal injury, promotes remyelination, relieves neuropathic pain, and improves conduction velocity and neural blood flow
Gene therapy	Immunomodulatory and reparative; promotes microvascular angiogenesis in peripheral nerves	Improves symptoms, short-term quality of life, sensory loss, and pain scores
Molecular targeting	Improves slowed nerve conduction and lowers nitrotyrosine and TNF-α	Reduces axonal atrophy and inflammation
Natural compounds		
Quercetin	Upregulates axonal growth factors, inhibits Rho/ROCK, activates AMPK/PGC-1α, and suppresses TLR4/NF-κB	Relieves neuropathic pain and sensory dysfunction
Curcumin	Regulates pJNK signaling and miRNAs (↓miR-21, ↑miR-146a)	Improves mechanical, cold, and thermal hyperalgesia, neuropathy scores, and reflexes
Resveratrol	Activates Nrf2 and SIRT1/PGC-1α, inhibits NF-κB, TRPV4, and P2X3	Reduces oxidative stress and inflammation, protects peripheral nerves, and relieves neuropathic pain
Alkaloids	Activate AMPK/PGC-1α, enhance Nrf2 antioxidant signaling, inhibit HDAC3, and regulate NRG1-ErbB2-PI3K-AKT	Reduce hyperalgesia and improve Schwann cell myelination
Capsaicin	At high concentration activates TRPV1, induces calcium overload, and functionally prunes hyperexcitable nociceptive endings	Markedly relieves pain without systemic adverse effects
Others	Modulate Nrf2, P2X7, SIRT1/p53, PI3K/AKT/mTOR, PPARγ/NF-κB, and AMPK signaling	Improve oxidative stress, neuroinflammation, mitochondrial dysfunction, apoptosis, and neuropathic pain
Non-pharmacological therapies		
TENS	Modulates sensory afferent input	Improves pain perception, though benefits in DN are limited
SCS	Promotes GABA release in the spinal dorsal horn and restores glutamate uptake	Produces sustained relief of chronic lower-limb pain and improves quality of life
Acupuncture/electroacupuncture/moxibustion	Downregulate spinal P2X4, inhibit NF-κB/CBS, activate SIRT1/PGC-1α, and suppress pro-inflammatory cytokines	May slow DPN progression, mitigate nerve injury, and reduce neuroinflammation, but stronger standardization and evidence are needed

### 5.1 Immunometabolic effects of classical glucose-lowering drugs

As a first-line oral glucose-lowering drug for T2DM, metformin acts not only by reducing hepatic gluconeogenesis and improving glucose uptake, but also exerts anti-inflammatory effects by targeting immunometabolism (240). Studies have shown that metformin can suppress T-cell immune responses through metabolic reprogramming, for example, by inhibiting mammalian target of rapamycin complex 1 (mTORC1) through an AMPK-independent pathway, thereby suppressing the transcription factors c-Myc and HIF-1α. In addition, metformin can improve T2DM-related B-cell dysfunction and reduce intrinsic inflammatory responses in B cells (241). Metformin can also inhibit the LPS-induced increase in fatty acid synthase (FASN) in macrophages, reduce palmitoylation of Akt protein, and thereby attenuate inflammatory responses by inhibiting activation of the Akt/MAPK signaling pathway (242). At the same time, by inhibiting mitochondrial complex I activity and reducing ROS generation, metformin specifically suppresses LPS-induced IL-1β production in macrophages and promotes production of the anti-inflammatory cytokine IL-10 (243).

Sodium–glucose cotransporter 2 inhibitors (SGLT2is), while improving glucose metabolism, also exhibit certain anti-inflammatory and immunomodulatory effects. Studies have shown that SGLT2is represented by canagliflozin can reduce expression of the proinflammatory markers ionized calcium-binding adapter molecule 1 (Iba1) and IL-6 and inhibit activation of macrophages and microglia in the nodose ganglion and hypothalamus (244).

Glucagon-like peptide-1 (GLP-1) receptor agonists likewise possess important immunometabolic regulatory and neuroprotective potential. GLP-1 receptors are widely distributed in the central and peripheral nervous systems, and their activation can suppress neuroinflammation and reduce oxidative stress. Studies have shown that exenatide can inhibit LPS-induced iNOS expression in macrophages by activating the cAMP/PKA pathway (245) and promote neurite outgrowth in DRG neurons (246). A randomized, double-blind, placebo-controlled trial showed that liraglutide significantly reduced levels of the proinflammatory cytokine IL-6 (247). In summary, some classical glucose-lowering drugs, while controlling blood glucose, can simultaneously exert neuroprotective effects by regulating the metabolic functions of immune cells and inflammatory signaling pathways, thereby providing new ideas for DPN intervention.

### 5.2 Targeting redox metabolism and the polyol pathway

Benfotiamine, a lipid-soluble derivative of thiamine (vitamin B1), can reduce aldose reductase activity by inhibiting multiple metabolic pathways, including the polyol pathway, the hexosamine pathway, and AGE formation, thereby alleviating hyperglycemia-induced metabolic dysregulation (248, 249). In addition, it can inhibit the pro-oxidative activity of microglia and exert direct antioxidant effects (250). Clinical studies have shown that 6 weeks of benfotiamine treatment (300 mg bid) can significantly improve DPN-related symptoms (251).

α-Lipoic acid is a regenerating endogenous antioxidant that also promotes glucose uptake and exerts anti-inflammatory effects (252, 253). Meta-analyses have shown that α-lipoic acid treatment can improve sensory neurological symptoms, but has no significant effect on muscle strength, vibration perception threshold, or nerve conduction velocity (254). Another systematic review likewise suggested that it is superior to placebo in relieving pain, but its effect on short-term numbness or paresthesia is not significant (255).

Aldose reductase inhibitors reduce NADPH consumption by blocking the conversion of glucose to sorbitol, thereby restoring NO generation and improving microvascular endothelial function. Agents such as epalrestat (256) and ranirestat (257) can both effectively reduce sorbitol accumulation and alleviate oxidative stress. Clinical studies have shown that, in a 3-year randomized open-label study, epalrestat effectively prevented deterioration in motor nerve conduction velocity (MNCV), minimum F-wave latency (MFWL), and vibration perception threshold (VPT) (258). Ranirestat, in turn, exerts some improvement in motor nerve function in patients with mild to moderate diabetic sensorimotor polyneuropathy, but no statistically significant difference has been observed in sensory nerve function (259).

The PKC pathway participates in multiple pathological processes induced by hyperglycemia, including inflammation, oxidative stress, and abnormal blood flow (28). PKCβ inhibitors such as LY-333531 can reduce p-PKCβ expression in the DRG, restore cyclic guanosine monophosphate (cGMP) levels, and improve hyperalgesia, suggesting that they may participate in neuroprotection by regulating the nNOS–cGMP signaling axis (260). A systematic review including six randomized controlled trials of the PKC inhibitor ruboxistaurin showed that it may improve total symptom scores (TSS) in patients with DPN, although not all studies have confirmed this (261).

### 5.3 Energy metabolism and neurotrophic support

Acetyl-L-carnitine (ALC) is a natural compound generated by the catalytic action of carnitine acetyltransferase. It plays a key role in mediating the transport of long-chain fatty acids into mitochondria for β-oxidation, promotes fatty-acid oxidation and mitochondrial function, and thereby helps maintain neural energy supply (262). Clinical trials have shown that ALC is comparable to mecobalamin in improving neuropathy symptom scores, disability scores, and neurophysiological parameters, and it has good tolerability (263). However, systematic reviews indicate that current evidence for the efficacy of ALC remains insufficient and of low quality, and no consistent conclusion has been reached regarding its pain-relieving effect in patients with DPN (264).

Neurotrophic factors such as nerve growth factor (NGF) and brain-derived neurotrophic factor (BDNF) play important roles in neural repair in DPN. NGF effectively improves hyperglycemia-induced sciatic nerve degeneration and demyelination by activating the PI3K/Akt/GSK3β and ERK1/2 signaling pathways and inhibiting excessive endoplasmic reticulum stress and its associated apoptosis (265). BDNF can regulate neuronal excitability and reduce abnormal neuronal firing, thereby alleviating pain symptoms (266). In addition, recombinant human mesencephalic astrocyte-derived neurotrophic factor can improve mechanical allodynia and nerve conduction function such as MNCV. Its mechanism may be related to inhibition of NF-κB and p38 MAPK signaling pathway activation, reduced macrophage infiltration in the DRG and sciatic nerve, and attenuation of inflammatory responses (113).

### 5.4 Cell-, gene-, and molecule-targeted therapies

Cell-, gene-, and molecule-targeted therapies promote neural repair and regeneration by regulating inflammatory responses and improving the immune microenvironment. Specific delivery of the anti-inflammatory cytokine IL-10 to the DRG using a recombinant herpes simplex virus vector can effectively regulate the local immune microenvironment (267), inhibit expression of the proinflammatory cytokine IL-1β and phosphorylation of p38 MAPK and PKC, and simultaneously upregulate expression of heat shock protein 70 (HSP-70), thereby exerting anti-inflammatory and neuroprotective effects. Intravenous administration of umbilical cord-derived mesenchymal stem cells (MSCs) can suppress neuronal injury, attenuate inflammatory responses, and promote remyelination (268). Intrathecal injection of bone marrow mesenchymal stem cells (BMSCs) can target the TLR2/MyD88/NF-κB pathway in microglia of the spinal dorsal horn and induce neuroprotection through secretion of tumor necrosis factor-stimulated gene-6 (TSG-6), thereby persistently relieving neuropathic pain (269). Human adipose-derived stem cells (hASCs) can alleviate mechanical and thermal hyperalgesia, promote recovery of neural function, and regulate the Th1/Th2 immune balance (270). Exosomes derived from MSCs can improve diabetic neurovascular dysfunction and axonal demyelination, and their mechanism is related to targeted inhibition of the TLR4/NF-κB signaling pathway and promotion of macrophage polarization from the M1 to the M2 phenotype (48). Transplantation of dental pulp stem cells can increase sensory nerve conduction velocity, nerve blood flow, capillary density, and epidermal nerve fiber density, and can also promote neurite outgrowth in the DRG and enhance Schwann-cell activity and myelination (271); at the same time, it can suppress monocyte/macrophage infiltration and TNF-α expression in the sciatic nerve and upregulate expression of the M2 macrophage marker gene CD206 (55), thereby improving the immune microenvironment. In addition, MSC subsets express neurotrophic factors such as BDNF and multiple neuromodulatory molecules, thereby promoting neuronal survival and regeneration (272).

VM202 is a nonviral plasmid DNA expressing two functional isoforms of human hepatocyte growth factor (HGF), and HGF can repair Schwann cells and promote peripheral nerve repair and regeneration (273). Clinical studies have shown that VM202 has good safety and tolerability and can improve symptoms and short-term quality of life (274), although some study endpoints have not fully met expectations (275). Intramuscular gene transfer of plasmid vascular endothelial growth factor (VEGF) can promote angiogenesis in the peripheral nerve microcirculation and, to some extent, improve sensory loss and pain scores (276). In terms of molecule-targeted therapy, poly(ADP-ribose) polymerase inhibitors can improve pathological changes such as slowed motor and sensory nerve conduction and atrophy of large myelinated fiber axons, and can reduce levels of nitrotyrosine and TNF-α in the sciatic nerve and spinal cord (277).

### 5.5 Natural compounds

The therapeutic value of natural compounds in DPN has gradually been supported by research. They may exert preventive and therapeutic effects by regulating, through multiple targets, immune inflammation, oxidative stress, mitochondrial dysfunction, and apoptosis (278). Compared with single-target drugs, the active components of natural compounds often achieve systemic intervention by modulating multiple signaling pathways, which gives them unique advantages in complex metabolic neuropathic diseases.

Flavonoids, a class of polyphenolic compounds widely present in plants, possess antioxidant, anti-inflammatory, and neuroprotective effects (279). Quercetin, a representative flavonoid compound, exerts neuroprotective effects and alleviates neuropathic pain and sensory dysfunction by reducing DNA damage, regulating inflammatory factors, and enhancing antioxidant enzyme activity (280). Studies have shown that quercetin can upregulate expression of the axon-guidance factors Slit-2 and Netrin-1 while inhibiting abnormal activation of the Rho/ROCK signaling pathway (281); it also improves mitochondrial function by activating the AMPK/PGC-1α pathway (282) and lowers inflammatory factor levels mediated by the TLR4/MyD88/NF-κB signaling pathway (283).

Curcumin is also a typical polyphenolic compound that can exert antioxidant and anti-inflammatory effects and improve mitochondrial function (284). Curcumin intervention can improve mechanical, cold, and thermal pain abnormalities, and its mechanism may be related to regulation of the p-JNK signaling pathway in DRG astrocytes and neurons (285). Curcumin can regulate multiple miRNA pathways, such as downregulating expression of miR-21, a key mediator of neuropathic pain (286), while upregulating expression of the anti-inflammatory miR-146a to inhibit NF-κB activation (287). Clinical studies have shown that nanocurcumin intervention improves metabolic indicators, including reductions in HbA1c and fasting blood glucose, as well as neuropathy outcomes, including increased Toronto Clinical Neuropathy Score, improved reflex function, and improved temperature sensitivity (288).

Resveratrol is a polyphenolic compound with multiple bioactivities (289). It can reduce oxidative stress and inflammatory responses by inhibiting the NF-κB pathway and activating Nrf2 signaling, thereby protecting peripheral nerves from apoptosis-related injury (290). At the same time, resveratrol can also improve mitochondrial function through the SIRT1/PGC-1α pathway (291) and upregulate HO-1 and other antioxidant enzymes to enhance endogenous antioxidant defense (292). Resveratrol can inhibit the TRPV4 channel and downregulate P2X3 receptor expression in the DRG and SDH, thereby alleviating neuropathic pain, apoptosis, and oxidative neurotoxicity (293, 294).

Alkaloids, including alkaloid extracts and isolated molecules from multiple species, also play important roles in diabetes and its complications. They regulate glucose metabolism and neural function through multiple pathways, including inhibition of α-glucosidase, blockade of PTP-1B, inhibition of DPP-IV, and improvement of insulin sensitivity (295). Berberine, a representative component, can alleviate mechanical and thermal hyperalgesia and exert neuroprotective effects by inhibiting oxidative stress and inflammatory responses (296). Berberine improves mitochondrial function and autophagic injury by activating the AMPK/PGC-1α signaling pathway and at the same time enhances the Nrf2-mediated antioxidant defense system, thereby suppressing neuronal injury and neuroinflammation (297). In addition, jatrorrhizine, one of the active components of Coptis chinensis, can improve Schwann-cell myelination by regulating the NRG1–ErbB2–PI3K–AKT signaling pathway through inhibition of histone deacetylase 3 (HDAC3) (298).

Capsaicin, as an agonist of the transient receptor potential vanilloid subtype 1 (TRPV1) receptor, has been used clinically for the management of neuropathic pain (299). The high-concentration capsaicin topical system (HCCTS) uses a special matrix technology to forcibly deliver high-concentration capsaicin to the epidermis and dermis, selectively activate TRPV1-positive nociceptive nerve endings, induce calcium overload that triggers processes such as mitochondrial dysfunction and cytoskeletal disassembly, and thereby “pruning” hyperexcitable nociceptive nerve endings thereby “prune” hyperexcitable pain-sensing nerve endings without affecting survival of neuronal cell bodies (300). Clinical studies have shown that the 8% capsaicin patch can significantly improve pain symptoms, with efficacy comparable to existing therapies and without systemic adverse effects or sensory impairment with efficacy comparable to other known treatments and without systemic adverse effects or sensory functional impairment (301).

Salvianolic acid A can effectively improve abnormalities in glucose and lipid metabolism. Its neuroprotective effect in DPN may be related to inhibition of Nrf2 expression, thereby improving mitochondrial function and reducing oxidative stress and neuroinflammatory responses (64). Salidroside can inhibit neuroinflammation and downregulate the P2X7 receptor, an important regulator of spinal inflammatory responses in diabetic rats, thereby improving neuropathic pain (302, 303). Astragaloside IV reduces the occurrence of mitochondria-dependent apoptosis by regulating the SIRT1/p53 pathway (304). Artesunate significantly improves hyperglycemia-induced sensory abnormalities and neural injury by inhibiting apoptosis in the sciatic nerve and activating the PI3K/AKT/mTOR signaling pathway to promote Schwann-cell survival (305). Magnolol, in turn, ameliorates mitochondrial dysfunction by regulating the PPARγ/MKP-7/JNK/SIRT1/LKB1/AMPK/PGC-1α signaling axis in DRG neurons and suppresses inflammatory responses and apoptosis in DRG neurons and the sciatic nerve through the PPARγ/NF-κB pathway (306).

### 5.6 Non-pharmacological therapies

Neuromodulation is an emerging method for the treatment of painful DN and mainly includes transcutaneous electrical nerve stimulation (TENS), intrathecal pain therapy, and spinal cord stimulation (SCS) (307). TENS is a noninvasive, nonpharmacological, and relatively low-cost intervention that can improve pain perception by modulating sensory nerve input. Existing evidence indicates that the efficacy of TENS in alleviating diabetic neuropathic pain is limited and that, compared with placebo or other electrostimulation therapies, it shows no significant advantage, although it may provide better analgesia in neuropathic pain associated with spinal cord injury (308). SCS modulates neuropathic pain through supraspinal feedback circuits and descending serotonergic fibers SCS regulates neuropathic pain through supraspinal feedback circuits and their descending serotonergic fibers (309), inducing inhibitory interneurons in the spinal dorsal horn to release γ-aminobutyric acid (GABA) (310). Clinical studies have shown that SCS can effectively relieve chronic pain symptoms in the lower limbs of patients with painful DN for as long as 5 years after treatment initiation (311). High-frequency spinal cord stimulation (HF-SCS) can partially restore glutamate uptake in the spinal cord and reduce abnormally elevated glutamate levels and the frequency of miniature excitatory postsynaptic currents, thereby alleviating neuropathic pain (312). A multicenter randomized controlled trial showed that 10-kHz HF-SCS is a safe and effective treatment for patients with refractory painful DN who respond poorly to conventional therapy, achieving significant, durable pain relief and improved quality of life over 6 months achieving significant and durable pain relief and improving quality of life within 6 months (313).

As a complementary and alternative medical approach, acupuncture has been widely used to treat various diseases and is beneficial and effective for pain relief (314). Studies have shown that acupuncture can downregulate P2X4 expression in spinal microglia, reduce inflammatory factor levels (315), and alleviate DPN progression. Electroacupuncture can slow neural injury by inhibiting expression of NF-κB and cystathionine β-synthase (CBS) in the DRG (316), as well as by activating the sirtuin 1 (SIRT1)/PGC-1α axis to promote mitochondrial biogenesis and endogenous antioxidation (317). In addition, moxibustion can significantly reduce expression levels of the proinflammatory cytokines IL-1β, IL-6, and IL-8 by inhibiting NF-κB and activating the Nrf2 signaling pathway (318), thereby alleviating neuroinflammatory responses.

### 5.7 Clinical evidence and translational progress

Current treatment strategies for DPN differ in terms of evidence from randomized controlled trials (RCTs), clinical feasibility, and long-term real-world effects. Because of the complex pathophysiological mechanisms of DPN and the lack of effective interventions, clinical treatment remains focused mainly on symptomatic control, and some patients ultimately still need to rely on opioids to help manage chronic pain. Although a certain number of high-quality RCTs have been conducted in this field, overall efficacy remains unsatisfactory, with most studies providing evidence only for short-term pain relief and with relatively high rates of treatment-related adverse events (235). In addition, although the number of clinical trials has gradually increased, there is a marked imbalance there is a marked disproportion in the assessment of biological outcomes in the evaluation of biological effects (319), so further research is needed to explore more effective treatment options and achieve clinical translation.

Evidence-based medicine indicates that intensive glycemic control can effectively prevent and delay the onset of DPN. Glycemic control provides clear benefits in reducing the risk of neuropathy in T1DM (320). Good glycemic management can not only reduce the incidence of distal symmetric polyneuropathy (DSPN), but also lower the risk of diabetic autonomic neuropathy (321, 322). However, for patients with established DPN, especially those with T2DM, intensive glycemic control alone has limited ability to reverse existing neural injury or substantially improve neural function. Multiple studies have shown that glycemic control in patients with T2DM cannot significantly reduce the incidence of neuropathy or can only delay disease progression to a certain extent (323, 324). Current clinical guidelines recommend mechanism-based therapies targeting oxidative stress, metabolic pathways, and microcirculatory dysfunction recommend drugs targeting the mechanisms of DPN mainly in the areas of anti-oxidative stress, metabolic pathway regulation, and microcirculatory improvement, including vasodilators such as prostaglandin E1, antioxidants such as α-lipoic acid, and aldose reductase inhibitors (325). In addition, the multitarget effects of natural compounds also have potential therapeutic value. However, existing evidence indicates that the clinical efficacy of the above agents is limited, and most therapeutic measures have not yet demonstrated clear and stable clinical benefits in large-scale, high-quality RCTs; their efficacy and long-term effects remain to be further validated (235).

At present, symptomatic treatment of DPN still focuses mainly on pain management, with gabapentin, pregabalin, duloxetine, and amitriptyline recommended as first-line drugs (236, 326). Clinical trials have confirmed that calcium channel modulators such as gabapentin (327) and pregabalin (328) can alleviate pain symptoms in patients with PDPN. In China, both crisugabalin and mirogabalin have also been approved for the treatment of PDPN and have become important first-line therapeutic options (329, 330). Systematic reviews and meta-analyses have shown that duloxetine has clear efficacy in reducing pain, lowering symptom burden, and improving quality of life (331). Clinical trials have shown that over a 12-week treatment period it exerts significantly better analgesic effects than placebo, and its safety in the Chinese population is consistent with previous studies (332). However, current pharmacological strategies still have limitations, including limited efficacy of monotherapy, additive adverse effects from combination therapy, and insufficient evidence regarding long-term safety.

With growing recognition of the limitations of conventional drugs, nonpharmacological therapies are receiving increasing attention. Studies have shown that SCS, especially 10-kHz high-frequency stimulation, has significant advantages in analgesic efficacy, safety, and improvement of quality of life (333). The SENZA-PDN randomized controlled trial showed that 10-kHz SCS achieved sustained and significant pain relief during 6 months of follow-up and significantly improved health-related quality of life, safely and effectively treating patients with refractory PDN (313). In addition, complementary and alternative therapies such as acupuncture and electroacupuncture have also shown some efficacy, although the quality of evidence still needs to be improved.

Given the multifactorial pathogenesis of DPN, current research is shifting from simple symptom relief toward disease modification. Multiple emerging therapeutic strategies remain in the stages of mechanistic validation and preclinical research, including MSCs, gene therapy, and molecularly targeted interventions molecule-targeted interventions, all of which have shown some therapeutic potential. However, these strategies still face multiple challenges in clinical translation, mainly including insufficient target specificity, unclear long-term safety, limited tissue-delivery efficiency, and lack of uniform clinical evaluation systems. At the same time, the design of DPN clinical trials itself also faces bottlenecks, mainly reflected in slow disease progression, with significant changes usually requiring 12–24 months to observe, high patient heterogeneity, and marked placebo effects (334). These factors together increase the difficulty of trial implementation and limit the sensitivity and reliability of efficacy evaluation.

Taken together, future therapeutic strategies may need to integrate multiple modalities, and exploration of new treatment strategies is also an important direction. Potential therapeutic strategies targeting immunometabolism in DPN cover multiple levels, including regulation of immune inflammation, restoration of metabolic balance, and neuroprotection, and aim to achieve more effective and durable therapeutic effects through intervention in key pathological mechanisms.

## 6 Discussion

The evidence synthesized in this review suggests that immunometabolic dysregulation driven by persistent metabolic stress is more likely to constitute an important pathological basis for the onset and progression of DPN, rather than merely representing a secondary phenomenon superimposed on hyperglycemia-induced injury. If DPN continues to be interpreted within a conventional framework that treats glucotoxicity, lipotoxicity, microvascular dysfunction, and neuronal energy imbalance as largely separate processes, then the marked heterogeneity of DPN in pain phenotypes, patterns of tissue injury progression, and capacity for neural repair often remains insufficiently explained. By contrast, an immunometabolic perspective offers a more integrative interpretive framework. Along the continuous peripheral nerve–dorsal root ganglion–spinal cord axis, immune cells, glial cells, Schwann cells, and the neural microvascular unit engage in sustained, bidirectional, and mutually amplifying signaling interactions. Based on the current body of evidence, these interactions appear less like accessory responses secondary to metabolic disturbance and more like a concentrated manifestation of the close interweaving of metabolic abnormalities with inflammatory processes. Together, they form a pathological network that drives the progressive evolution of neural injury. From this perspective, DPN may be more appropriately understood as a complex neuropathological process involving the combined contributions of metabolic derangement, neuroinflammation, and microenvironmental disequilibrium.

Further consideration suggests that the pathways discussed above—oxidative/nitrosative stress, aberrant AGEs–RAGE signaling, and gut microbial dysbiosis—are unlikely to represent isolated secondary abnormalities. More plausibly, they may serve as key pathological nodes through which metabolic disturbance is translated into sustained immune activation. Persistent hyperglycemia and lipotoxicity can induce excessive production of ROS and disrupt NO signaling, followed by mitochondrial dysfunction, protein nitration, lipid peroxidation, and DNA damage, while continuously activating inflammatory pathways such as NF-κB, MAPK, and the NLRP3 inflammasome within a certain range. At the same time, gut microbiota dysbiosis and altered microbial metabolites may further amplify systemic inflammation and redox imbalance by increasing endotoxin burden, weakening short-chain fatty acid-mediated immune homeostasis, and disrupting intestinal barrier integrity. From the standpoint of biological interpretability, these pathways are worth considering within a unified framework not only because they intersect with one another, but also because they can simultaneously act on neurons, Schwann cells, and neural microvascular endothelial cells, thereby inducing cellular metabolic reprogramming, disrupting redox homeostasis, and sustaining proinflammatory signaling. In turn, this promotes a progressively self-amplifying pathological feedback loop. For this reason, placing metabolic abnormalities, amplification of immune effector responses, and neural tissue injury within the same analytical framework may better explain the internal coherence underlying the complex course of DPN, while also providing a rationale for identifying intervention targets of greater biological relevance.

It is equally important to note that DPN-related immune responses are not static events; rather, they exhibit relatively clear temporal features, tissue specificity, and cell type dependence. Taken together with the immune-cell landscape outlined above, the available evidence suggests that under conditions of sustained hyperglycemia and lipotoxicity, monocytes/macrophages, T cells, glial cells, and Schwann cells form a relatively central cellular network in the immunopathology of DPN. Within this network, proinflammatory macrophage activation, imbalance among T-cell subsets, and persistent glial activation are closely associated with altered neuronal excitability, myelin injury, barrier disruption, and progressive deterioration of the local neural microenvironment. Schwann cells and vascular endothelial cells, moreover, should not be viewed merely as passive targets of injury; they also participate in chemokine release, barrier dysfunction, and inflammatory amplification, thereby further promoting immune-cell recruitment and activation. By comparison, although certain anti-inflammatory mediators, protective macrophage responses, or compensatory immunoregulatory mechanisms suggest the presence of endogenous repair attempts, their magnitude is usually insufficient to counteract the sustained burden imposed by hyperglycemia, lipotoxicity, and oxidative stress. As for other immune cell types, such as natural killer cells, eosinophils, and basophils, the currently available evidence in DPN remains largely supportive rather than definitive, and more direct histological, temporal, and functional evidence is still needed. Put differently, an understanding of DPN immunopathology based solely on single cell types or isolated mediators is likely to have limited explanatory power. A more informative approach may be to emphasize the dynamic interactions among different cellular populations and the shifting dominance of these populations across different stages of disease progression.

From a translational perspective, strengthening our understanding of immunometabolic mechanisms may be valuable not only because it refines the pathological interpretation of DPN, but also because it may help shift therapeutic strategies from simple symptom control toward disease-course intervention. Current clinical management still centers primarily on glycemic control, risk-factor modification, and pain relief. Tight glycemic control has relatively clear preventive value for early neural injury, particularly in the setting of T1DM. However, for established DPN—especially T2DM-related DPN—its capacity to reverse existing injury remains limited. To some extent, this suggests that therapeutic strategies directed solely at correcting metabolic abnormalities may be insufficient to address the complex and multilayered pathogenesis of DPN. By contrast, intervention strategies aimed at immunometabolic remodeling—whether involving the immunometabolic effects of classical glucose-lowering agents, drugs targeting redox metabolism and the polyol pathway, neurotrophic support, cell- or gene-based therapies, natural bioactive compounds, or neuromodulatory and other non-pharmacological approaches—may be better positioned to act simultaneously on the inflammatory microenvironment, redox balance, neural blood supply, and regenerative capacity, thereby improving the durability and clinical relevance of treatment. Nevertheless, judged by the current strength of evidence, most of these approaches remain at the stage of mechanistic validation, animal experimentation, or early clinical observation, and only a limited number of interventions have demonstrated clear benefit in large cohorts, with long-term follow-up and stable clinical endpoints. Future studies would therefore benefit from advancing mechanistic validation and clinical translation on the basis of patient stratification, while integrating pain phenotypes, immune-inflammatory status, redox indicators, metabolic characteristics, and microbial ecological information into study design. Composite endpoints encompassing neural function, structural injury, and quality of life may also be needed to more accurately assess the true benefit of immunometabolism-targeted interventions.

Overall, immunometabolic dysregulation should not be regarded as a secondary feature of the pathological process in DPN. At least on the basis of the evidence synthesized in this review, it appears more consistent with a core mechanistic framework linking metabolic abnormalities, neuroinflammation, structural injury, and variability in therapeutic response. This perspective not only improves the interpretability of the pathological heterogeneity of DPN, but also provides a more integrated theoretical basis for precision stratification and targeted intervention.

## References

[1] Savelieff MG Elafros MA Viswanathan V Jensen TS Bennett DL Feldman EL . The global and regional burden of diabetic peripheral neuropathy. Nat Rev Neurol. (2025) 21:17–31. doi: 10.1038/s41582-024-01041-y 39639140 PMC13011988

[2] Eid SA Rumora AE Beirowski B Bennett DL Hur J Savelieff MG . New perspectives in diabetic neuropathy. Neuron. (2023) 111:2623–41. doi: 10.1016/j.neuron.2023.05.003 37263266 PMC10525009

[3] Vincent AM Callaghan BC Smith AL Feldman EL . Diabetic neuropathy: cellular mechanisms as therapeutic targets. Nat Rev Neurol. (2011) 7:573–83. doi: 10.1038/nrneurol.2011.137 21912405

[4] Calvo M Dawes JM Bennett DLH . The role of the immune system in the generation of neuropathic pain. Lancet Neurol. (2012) 11:629–42. doi: 10.1016/S1474-4422(12)70134-5 22710756

[5] Obrosova IG Drel VR Pacher P Ilnytska O Wang ZQ Stevens MJ . Oxidative-nitrosative stress and poly(ADP-ribose) polymerase (PARP) activation in experimental diabetic neuropathy: the relation is revisited. Diabetes. (2005) 54:3435–41. doi: 10.2337/diabetes.54.12.3435 16306359 PMC2228259

[6] Jeffcoate WJ Game F Cavanagh PR . The role of proinflammatory cytokines in the cause of neuropathic osteoarthropathy (acute Charcot foot) in diabetes. Lancet. (2005) 366:2058–61. doi: 10.1016/S0140-6736(05)67029-8 16338454

[7] Xue T Zhang X Xing Y Liu S Zhang L Wang X . Advances about immunoinflammatory pathogenesis and treatment in diabetic peripheral neuropathy. Front Pharmacol. (2021) 12:748193. doi: 10.3389/fphar.2021.748193 34671261 PMC8520901

[8] Shi X Chen Y Nadeem L Xu G . Beneficial effect of TNF-α inhibition on diabetic peripheral neuropathy. J Neuroinflamm. (2013) 10:69. doi: 10.1186/1742-2094-10-69 23735240 PMC3679954

[9] Lukic IK Humpert PM Nawroth PP Bierhaus A . The RAGE pathway: activation and perpetuation in the pathogenesis of diabetic neuropathy. Ann N Y Acad Sci. (2008) 1126:76–80. doi: 10.1196/annals.1433.059 18448798

[10] Takeshita Y Sato R Kanda T . Blood-nerve barrier (BNB) pathology in diabetic peripheral neuropathy and *in vitro* human BNB model. Int J Mol Sci. (2020) 22:62. doi: 10.3390/ijms22010062 33374622 PMC7793499

[11] Ma X Nan F Liang H Shu P Fan X Song X . Excessive intake of sugar: An accomplice of inflammation. Front Immunol. (2022) 13:988481. doi: 10.3389/fimmu.2022.988481 36119103 PMC9471313

[12] Daulhac L Maffre V Mallet C Etienne M Privat AMRE Kowalski-Chauvel A . Phosphorylation of spinal N-methyl-d-aspartate receptor NR1 subunits by extracellular signal-regulated kinase in dorsal horn neurons and microglia contributes to diabetes-induced painful neuropathy. Eur J Pain. (2011) 15:161–69. doi: 10.1016/j.ejpain.2010.06.003 20594879

[13] Obrosova IG Drel VR Oltman CL Mashtalir N Tibrewala J Groves JT . Role of nitrosative stress in early neuropathy and vascular dysfunction in streptozotocin-diabetic rats. Am J Physiol Endocrinol Metab. (2007) 293:E1645–55. doi: 10.1152/ajpendo.00479.2007 17911342

[14] Purwata TE . High TNF-alpha plasma levels and macrophages iNOS and TNF-alpha expression as risk factors for painful diabetic neuropathy. J Pain Res. (2011) 4:169–75. doi: 10.2147/JPR.S21751 21811392 PMC3141833

[15] Vincent AM McLean LL Backus C Feldman EL . Short-term hyperglycemia produces oxidative damage and apoptosis in neurons. FASEB J. (2005) 19:638–40. doi: 10.1096/fj.04-2513fje 15677696

[16] Sloan G Selvarajah D Tesfaye S . Pathogenesis, diagnosis and clinical management of diabetic sensorimotor peripheral neuropathy. Nat Rev Endocrinol. (2021) 17:400–20. doi: 10.1038/s41574-021-00496-z 34050323

[17] Roy Chowdhury SK Smith DR Saleh A Schapansky J Marquez A Gomes S . Impaired adenosine monophosphate-activated protein kinase signalling in dorsal root ganglia neurons is linked to mitochondrial dysfunction and peripheral neuropathy in diabetes. Brain. (2012) 135:1751–66. doi: 10.1093/brain/aws097 22561641 PMC3359752

[18] Vincent AM Hayes JM McLean LL Vivekanandan-Giri A Pennathur S Feldman EL . Dyslipidemia-induced neuropathy in mice: the role of oxLDL/LOX-1. Diabetes. (2009) 58:2376–85. doi: 10.2337/db09-0047 19592619 PMC2750230

[19] Nishikawa T Edelstein D Du XL Yamagishi S Matsumura T Kaneda Y . Normalizing mitochondrial superoxide production blocks three pathways of hyperglycaemic damage. Nature. (2000) 404:787–90. doi: 10.1038/35008121 10783895

[20] Du XL Edelstein D Rossetti L Fantus IG Goldberg H Ziyadeh F . Hyperglycemia-induced mitochondrial superoxide overproduction activates the hexosamine pathway and induces plasminogen activator inhibitor-1 expression by increasing Sp1 glycosylation. Proc Natl Acad Sci USA. (2000) 97:12222–26. doi: 10.1073/pnas.97.22.12222 11050244 PMC17322

[21] Schmeichel AM Schmelzer JD Low PA . Oxidative injury and apoptosis of dorsal root ganglion neurons in chronic experimental diabetic neuropathy. Diabetes. (2003) 52:165–71. doi: 10.2337/diabetes.52.1.165 12502508

[22] Kennedy JM Zochodne DW . Experimental diabetic neuropathy with spontaneous recovery: is there irreparable damage? Diabetes. (2005) 54:830–37. doi: 10.2337/diabetes.54.3.830 15734862

[23] Coppey LJ Gellett JS Davidson EP Dunlap JA Lund DD Yorek MA . Effect of antioxidant treatment of streptozotocin-induced diabetic rats on endoneurial blood flow, motor nerve conduction velocity, and vascular reactivity of epineurial arterioles of the sciatic nerve. Diabetes. (2001) 50:1927–37. doi: 10.2337/diabetes.50.8.1927 11473057

[24] O'Neill LAJ Kishton RJ Rathmell J . A guide to immunometabolism for immunologists. Nat Rev Immunol. (2016) 16:553–65. doi: 10.1038/nri.2016.70 27396447 PMC5001910

[25] Feldman EL Nave KA Jensen TS Bennett DLH . New horizons in diabetic neuropathy: mechanisms, bioenergetics, and pain. Neuron. (2017) 93:1296–313. doi: 10.1016/j.neuron.2017.02.005 28334605 PMC5400015

[26] Tesfaye S Boulton AJM Dyck PJ Freeman R Horowitz M Kempler P . Diabetic neuropathies: update on definitions, diagnostic criteria, estimation of severity, and treatments. Diabetes Care. (2010) 33:2285–93. doi: 10.2337/dc10-1303 20876709 PMC2945176

[27] Vincent AM Brownlee M Russell JW . Oxidative stress and programmed cell death in diabetic neuropathy. Ann N Y Acad Sci. (2002) 959:368–83. doi: 10.1111/j.1749-6632.2002.tb02108.x 11976211

[28] Brownlee M . The pathobiology of diabetic complications: a unifying mechanism. Diabetes. (2005) 54:1615–25. doi: 10.2337/diabetes.54.6.1615 15919781

[29] Obrosova IG Van Huysen C Fathallah L Cao XC Greene DA Stevens MJ . An aldose reductase inhibitor reverses early diabetes-induced changes in peripheral nerve function, metabolism, and antioxidative defense. FASEB J. (2002) 16:123–25. doi: 10.1096/fj.01-0603fje 11709499

[30] Vincent AM Perrone L Sullivan KA Backus C Sastry AM Lastoskie C . Receptor for advanced glycation end products activation injures primary sensory neurons via oxidative stress. Endocrinology. (2007) 148:548–58. doi: 10.1210/en.2006-0073 17095586

[31] Wautier MP Chappey O Corda S Stern DM Schmidt AM Wautier JL . Activation of NADPH oxidase by AGE links oxidant stress to altered gene expression via RAGE. Am J Physiol Endocrinol Metab. (2001) 280:E685–94. doi: 10.1152/ajpendo.2001.280.5.E685 11287350

[32] Obrosova IG Ilnytska O Lyzogubov VV Pavlov IA Mashtalir N Nadler JL . High-fat diet induced neuropathy of pre-diabetes and obesity: effects of "healthy" diet and aldose reductase inhibition. Diabetes. (2007) 56:2598–608. doi: 10.2337/db06-1176 17626889

[33] Gonçalves NDAP Vægter CB Andersen H Østergaard L Calcutt NA Jensen TS . Schwann cell interactions with axons and microvessels in diabetic neuropathy. Nat Rev Neurol. (2017) 13:135–47. doi: 10.1038/nrneurol.2016.201 28134254 PMC7391875

[34] Chowdhury SKR Dobrowsky RT Fernyhough P . Nutrient excess and altered mitochondrial proteome and function contribute to neurodegeneration in diabetes. Mitochondrion. (2011) 11:845–54. doi: 10.1016/j.mito.2011.06.007 21742060 PMC3375692

[35] Uçeyler N Rogausch JP Toyka KV Sommer C . Differential expression of cytokines in painful and painless neuropathies. Neurology. (2007) 69:42–9. doi: 10.1212/01.wnl.0000265062.92340.a5 17606879

[36] Tuck RR Schmelzer JD Low PA . Endoneurial blood flow and oxygen tension in the sciatic nerves of rats with experimental diabetic neuropathy. Brain. (1984) 107:935–50. doi: 10.1093/brain/107.3.935 6478183

[37] Obrosova IG Mabley JG Zsengellér Z Charniauskaya T Abatan OI Groves JT . Role for nitrosative stress in diabetic neuropathy: evidence from studies with a peroxynitrite decomposition catalyst. FASEB J. (2005) 19:401–03. doi: 10.1096/fj.04-1913fje 15611153

[38] Obrosova IG Li F Abatan OI Forsell MA Komjáti K Pacher P . Role of poly(ADP-ribose) polymerase activation in diabetic neuropathy. Diabetes. (2004) 53:711–20. doi: 10.2337/diabetes.53.3.711 14988256

[39] Lupachyk S Shevalye H Maksimchyk Y Drel VR Obrosova IG . PARP inhibition alleviates diabetes-induced systemic oxidative stress and neural tissue 4-hydroxynonenal adduct accumulation: correlation with peripheral nerve function. Free Radic Biol Med. (2011) 50:1400–09. doi: 10.1016/j.freeradbiomed.2011.01.037 21300148 PMC3081984

[40] Sommer C Schmidt C George A . Hyperalgesia in experimental neuropathy is dependent on the TNF receptor 1. Exp Neurol. (1998) 151:138–42. doi: 10.1006/exnr.1998.6797 9582261

[41] Herder C Lankisch M Ziegler D Rathmann W Koenig W Illig T . Subclinical inflammation and diabetic polyneuropathy: MONICA/KORA Survey F3 (Augsburg, Germany). Diabetes Care. (2009) 32:680–82. doi: 10.2337/dc08-2011 19131463 PMC2660451

[42] Yang J Yang X Wu G Huang F Shi X Wei W . Gut microbiota modulate distal symmetric polyneuropathy in patients with diabetes. Cell Metab. (2023) 35:1548–62. doi: 10.1016/j.cmet.2023.06.010 37451270

[43] Mann ER Lam YK Uhlig HH . Short-chain fatty acids: linking diet, the microbiome and immunity. Nat Rev Immunol. (2024) 24:577–95. doi: 10.1038/s41577-024-01014-8 38565643

[44] Horiachok M Potapova K Ivanykovych T Yerokhovych V Ilkiv Y Sokolova L . Integrating gut microbiota into multidisciplinary perspectives on diabetic neuropathy. Front Endocrinol (Lausanne). (2025) 16:1710868. doi: 10.3389/fendo.2025.1710868 41255529 PMC12620251

[45] Chmielarz M Sobieszczańska B Środa-Pomianek K . Metabolic endotoxemia: From the gut to neurodegeneration. Int J Mol Sci. (2024) 25:7006. doi: 10.3390/ijms25137006 39000116 PMC11241432

[46] Allwright M Karrasch JF O'Brien JA Guennewig B Austin PJ . Machine learning analysis of the UK Biobank reveals prognostic and diagnostic immune biomarkers for polyneuropathy and neuropathic pain in diabetes. Diabetes Res Clin Pract. (2023) 201:110725. doi: 10.1016/j.diabres.2023.110725 37211253

[47] Li W Guo J Chen J Yao H Mao R Li C . Identification of immune infiltration and the potential biomarkers in diabetic peripheral neuropathy through bioinformatics and machine learning methods. Biomolecules. (2022) 13:39. doi: 10.3390/biom13010039 36671424 PMC9855866

[48] Fan B Li C Szalad A Wang L Pan W Zhang R . Mesenchymal stromal cell-derived exosomes ameliorate peripheral neuropathy in a mouse model of diabetes. Diabetologia. (2020) 63:431–43. doi: 10.1007/s00125-019-05043-0 31740984 PMC6949414

[49] Agarwal N Helmstädter J Rojas DR Bali KK Gangadharan V Kuner R . Evoked hypoalgesia is accompanied by tonic pain and immune cell infiltration in the dorsal root ganglia at late stages of diabetic neuropathy in mice. Mol Pain. (2018) 14:1744806918817975. doi: 10.1177/1744806918817975 30453826 PMC6311571

[50] Adki KM Kulkarni YA . Biomarkers in diabetic neuropathy. Arch Physiol Biochem. (2023) 129:460–75. doi: 10.1080/13813455.2020.1837183 33186087

[51] Chen C Zhu L Li W Jiang Y Zhang Z Zhao Y . Hyperglycemia promotes SIRT3-mediated deacetylation of SARM1 to exacerbate diabetic peripheral neuropathy in mice. Proc Natl Acad Sci USA. (2026) 123:e2517110123. doi: 10.1073/pnas.2517110123 41512034 PMC12799148

[52] Wang Q Zhang S Liu T Wang H Liu K Wang Q . Sarm1/Myd88-5 regulates neuronal intrinsic immune response to traumatic axonal injuries. Cell Rep. (2018) 23:716–24. doi: 10.1016/j.celrep.2018.03.071 29669278

[53] Shinouchi R Shibata K Jono S Hasumi K Nobe K . SMTP-44D exerts antioxidant and anti-inflammatory effects through its soluble epoxide hydrolase inhibitory action in immortalized mouse Schwann cells upon high glucose treatment. Int J Mol Sci. (2022) 23:5187. doi: 10.3390/ijms23095187 35563575 PMC9104197

[54] Adki KM Kulkarni YA . Neuroprotective effect of paeonol in streptozotocin-induced diabetes in rats. Life Sci. (2021) 271:119202. doi: 10.1016/j.lfs.2021.119202 33577853

[55] Omi M Hata M Nakamura N Miyabe M Kobayashi Y Kamiya H . Transplantation of dental pulp stem cells suppressed inflammation in sciatic nerves by promoting macrophage polarization towards anti-inflammation phenotypes and ameliorated diabetic polyneuropathy. J Diabetes Investig. (2016) 7:485–96. doi: 10.1111/jdi.12452 27181261 PMC4931198

[56] Sun JJ Tang L Zhao XP Xu JM Xiao Y Li H . Infiltration of blood-derived macrophages contributes to the development of diabetic neuropathy. J Immunol Res. (2019) 2019:7597382. doi: 10.1155/2019/7597382 31534976 PMC6732633

[57] Li H Zou L Long Z Zhan J . Immunometabolic alterations in type 2 diabetes mellitus revealed by single-cell RNA sequencing: insights into subtypes and therapeutic targets. Front Immunol. (2024) 15:1537909. doi: 10.3389/fimmu.2024.1537909 39877357 PMC11772204

[58] Jones N Blagih J Zani F Rees A Hill DG Jenkins BJ . Fructose reprogrammes glutamine-dependent oxidative metabolism to support LPS-induced inflammation. Nat Commun. (2021) 12:1209. doi: 10.1038/s41467-021-21461-4 33619282 PMC7900179

[59] Huang L Feng B Pang J Chen JG Guo YH Du X . Effect of endoplasmic reticulum stress on the activation of high glucose - induced monocytes. Chin. J. Nephrol. (2013) 29(3).

[60] Restaino RM Deo SH Parrish AR Fadel PJ Padilla J . Increased monocyte-derived reactive oxygen species in type 2 diabetes: role of endoplasmic reticulum stress. Exp Physiol. (2017) 102:139–53. doi: 10.1113/EP085794 27859785 PMC5600886

[61] Wu CH Chang YH Hsu CL Chen SY Yen GC . High glucose reduces Nrf2-dependent cRAGE release and enhances inflammasome-dependent IL-1β production in monocytes: the modulatory effects of EGCG. Food Sci Hum Wellness. (2024) 13:1531–42. doi: 10.26599/FSHW.2022.9250129

[62] He F Ru X Wen T . NRF2, a transcription factor for stress response and beyond. Int J Mol Sci. (2020) 21:4777. doi: 10.3390/ijms21134777 32640524 PMC7369905

[63] Zhong Q Mishra M Kowluru RA . Transcription factor Nrf2-mediated antioxidant defense system in the development of diabetic retinopathy. Invest Ophthalmol Vis Sci. (2013) 54:3941–8. doi: 10.1167/iovs.13-11598 23633659 PMC3676188

[64] Xu C Hou B He P Ma P Yang X Yang X . Neuroprotective effect of salvianolic acid A against diabetic peripheral neuropathy through modulation of Nrf2. Oxid Med Cell Longev. (2020) 2020:6431459. doi: 10.1155/2020/6431459 32184918 PMC7063195

[65] Wu KY Deng F Mao XY Zhou D Shen WG . Ferroptosis involves in Schwann cell death in diabetic peripheral neuropathy. Open Med (Wars). (2023) 18:20230809. doi: 10.1515/med-2023-0809 37829841 PMC10566555

[66] Zhu T Meng Q Ji J Zhang L Lou X . TLR4 and Caveolin-1 in monocytes are associated with inflammatory conditions in diabetic neuropathy. Clin Transl Sci. (2017) 10:178–84. doi: 10.1111/cts.12434 27981790 PMC5421735

[67] Wang Y Luo W Han J Khan ZA Fang Q Jin Y . MD2 activation by direct AGE interaction drives inflammatory diabetic cardiomyopathy. Nat Commun. (2020) 11:2148. doi: 10.1038/s41467-020-15978-3 32358497 PMC7195432

[68] Sivanantham A Alktaish W Murugeasan S Gong B Lee H Jin Y . Caveolin-1 regulates OMV-induced macrophage pro-inflammatory activation and multiple Toll-like receptors. Front Immunol. (2023) 14:1044834. doi: 10.3389/fimmu.2023.1044834 36817491 PMC9933776

[69] Hayashi T Juliet PAR Miyazaki A Ignarro LJ Iguchi A . High glucose downregulates the number of caveolae in monocytes through oxidative stress from NADPH oxidase: implications for atherosclerosis. Biochim Biophys Acta. (2007) 1772:364–72. doi: 10.1016/j.bbadis.2006.11.011 17240121

[70] Wu CH Yeh CT Shih PH Yen GC . Dietary phenolic acids attenuate multiple stages of protein glycation and high-glucose-stimulated proinflammatory IL-1beta activation by interfering with chromatin remodeling and transcription in monocytes. Mol Nutr Food Res. (2010) 54:S127–40. doi: 10.1002/mnfr.200900395 20306478

[71] Neeb L Hellen P Boehnke C Hoffmann J Schuh-Hofer S Dirnagl U . IL-1β stimulates COX-2 dependent PGE₂ synthesis and CGRP release in rat trigeminal ganglia cells. PloS One. (2011) 6:e17360. doi: 10.1371/journal.pone.0017360 21394197 PMC3048859

[72] Jiang J Borisenko GG Osipov A Martin I Chen R Shvedova AA . Arachidonic acid-induced carbon-centered radicals and phospholipid peroxidation in cyclo-oxygenase-2-transfected PC12 cells. J Neurochem. (2004) 90:1036–49. doi: 10.1111/j.1471-4159.2004.02577.x 15312159

[73] Kosuge Y Nango H Kasai H Yanagi T Mawatari T Nishiyama K . Generation of cellular reactive oxygen species by activation of the EP2 receptor contributes to prostaglandin E2-induced cytotoxicity in motor neuron-like NSC-34 cells. Oxid Med Cell Longev. (2020) 2020:6101838. doi: 10.1155/2020/6101838 32411331 PMC7201578

[74] Sung CS Wen ZH Chang WK Ho ST Tsai SK Chang YC . Intrathecal interleukin-1beta administration induces thermal hyperalgesia by activating inducible nitric oxide synthase expression in the rat spinal cord. Brain Res. (2004) 1015:145–53. doi: 10.1016/j.brainres.2004.04.068 15223378

[75] Tian J Song T Wang H Wang W Ma X Hu Y . Toll-like receptor 2 antagonist ameliorates type 2 diabetes mellitus associated neuropathic pain by repolarizing pro-inflammatory macrophages. Neurochem Res. (2021) 46:2276–84. doi: 10.1007/s11064-021-03365-3 34081245

[76] Hangping Z Ling H Lijin J Wenting Z Xiaoxia L Qi Z . The preventive effect of IL-1beta antagonist on diabetic peripheral neuropathy. Endocr Metab Immune Disord Drug Targets. (2020) 20:753–9. doi: 10.2174/1871530319666191022114139 31642797

[77] Liu YJ Saini A Cohen DJ Ooi BS . Modulation of macrophage proliferation by hyperglycemia. Mol Cell Endocrinol. (1995) 114:187–92. doi: 10.1016/0303-7207(95)96799-n 8674843

[78] Edgar L Akbar N Braithwaite AT Krausgruber T Gallart-Ayala HC Bailey J . Hyperglycemia induces trained immunity in macrophages and their precursors and promotes atherosclerosis. Circulation. (2021) 144:961–82. doi: 10.1161/CIRCULATIONAHA.120.046464 34255973 PMC8448412

[79] Witcoski Junior L de Lima JD Somensi AG de Souza Santos LB Paschoal GL Uada TS . Metabolic reprogramming of macrophages in the context of type 2 diabetes. Eur J Med Res. (2024) 29:497. doi: 10.1186/s40001-024-02069-y 39407333 PMC11481356

[80] Di BB Li HW Li WP Shen XH Sun ZJ Wu X . Pioglitazone inhibits high glucose-induced expression of receptor for advanced glycation end products in coronary artery smooth muscle cells. Mol Med Rep. (2015) 11:2601–7. doi: 10.3892/mmr.2014.3113 25523934 PMC4337739

[81] Giacco F Brownlee M . Oxidative stress and diabetic complications. Circ Res. (2010) 107:1058–70. doi: 10.1161/CIRCRESAHA.110.223545 21030723 PMC2996922

[82] Tannahill GM Curtis AM Adamik J Palsson-McDermott EM McGettrick AF Goel G . Succinate is an inflammatory signal that induces IL-1β through HIF-1α. Nature. (2013) 496:238–42. doi: 10.1038/nature11986 23535595 PMC4031686

[83] Kan HW Hsieh JH Chien HF Lin YAH Yeh TY Chao CC . CD40-mediated HIF-1α expression underlying microangiopathy in diabetic nerve pathology. Dis Model Mech. (2018) 11(4):dmm033647. doi: 10.1242/dmm.033647 29549140 PMC5963861

[84] Zhao Z Ming Y Li X Tan H He X Yang L . Hyperglycemia aggravates periodontitis via autophagy impairment and ROS-inflammasome-mediated macrophage pyroptosis. Int J Mol Sci. (2023) 24(7):6309. doi: 10.3390/ijms24076309 37047282 PMC10094233

[85] Rendra E Riabov V Mossel DM Sevastyanova T Harmsen MC Kzhyshkowska J . Reactive oxygen species (ROS) in macrophage activation and function in diabetes. Immunobiology. (2019) 224:242–53. doi: 10.1016/j.imbio.2018.11.010 30739804

[86] Msheik Z El Massry M Rovini A Billet F Desmoulière A . The macrophage: a key player in the pathophysiology of peripheral neuropathies. J Neuroinflamm. (2022) 19:97. doi: 10.1186/s12974-022-02454-6 35429971 PMC9013246

[87] Eid SPA El Massry M Hichor M Haddad M Grenier J Dia B . Targeting the NADPH oxidase-4 and liver X receptor pathway preserves Schwann cell integrity in diabetic mice. Diabetes. (2020) 69:448–64. doi: 10.2337/db19-0517 31882567

[88] Hackel D Pflücke D Neumann A Viebahn J Mousa S Wischmeyer E . The connection of monocytes and reactive oxygen species in pain. PloS One. (2013) 8:e63564. doi: 10.1371/journal.pone.0063564 23658840 PMC3642180

[89] Yang H Li S . Transient receptor potential ankyrin 1 (TRPA1) channel and neurogenic inflammation in pathogenesis of asthma. Med Sci Monit. (2016) 22:2917–23. doi: 10.12659/msm.896557 27539812 PMC5003164

[90] Koivisto A Pertovaara A . Transient receptor potential ankyrin 1 (TRPA1) ion channel in the pathophysiology of peripheral diabetic neuropathy. Scand J Pain. (2013) 4:129–36. doi: 10.1016/j.sjpain.2012.11.001 29913916

[91] Hervera A De Virgiliis F Palmisano I Zhou L Tantardini E Kong G . Reactive oxygen species regulate axonal regeneration through the release of exosomal NADPH oxidase 2 complexes into injured axons. Nat Cell Biol. (2018) 20:307–19. doi: 10.1038/s41556-018-0039-x 29434374

[92] Wu H Wang M Li X Shao Y . The metaflammatory and immunometabolic role of macrophages and microglia in diabetic retinopathy. Hum Cell. (2021) 34:1617–28. doi: 10.1007/s13577-021-00580-6 34324139

[93] Cheng CI Chen PH Lin YC Kao YH . High glucose activates Raw264.7 macrophages through RhoA kinase-mediated signaling pathway. Cell Signal. (2015) 27:283–92. doi: 10.1016/j.cellsig.2014.11.012 25446262

[94] Xu X Qi X Shao Y Li Y Fu X Feng S . High glucose induced-macrophage activation through TGF-β-activated kinase 1 signaling pathway. Inflammation Res. (2016) 65:655–64. doi: 10.1007/s00011-016-0948-8 27153994

[95] Al-Rashed F Sindhu S Arefanian H Al Madhoun A Kochumon S Thomas R . Repetitive intermittent hyperglycemia drives the M1 polarization and inflammatory responses in THP-1 macrophages through the mechanism involving the TLR4-IRF5 pathway. Cells. (2020) 9:1892. doi: 10.3390/cells9081892 32806763 PMC7463685

[96] Pavlou S Lindsay J Ingram R Xu H Chen M . Sustained high glucose exposure sensitizes macrophage responses to cytokine stimuli but reduces their phagocytic activity. BMC Immunol. (2018) 19:24. doi: 10.1186/s12865-018-0261-0 29996768 PMC6042333

[97] Vlassara H Brownlee M Cerami A . Accumulation of diabetic rat peripheral nerve myelin by macrophages increases with the presence of advanced glycosylation endproducts. J Exp Med. (1984) 160:197–207. doi: 10.1084/jem.160.1.197 6736870 PMC2187417

[98] Yue S Zhou HM Zhu JJ Rao JH Busuttil RW Kupiec-Weglinski JW . Hyperglycemia and liver ischemia reperfusion injury: a role for the advanced glycation endproduct and its receptor pathway. Am J Transplant. (2015) 15:2877–87. doi: 10.1111/ajt.13360 26112980 PMC9438741

[99] Osonoi S Mizukami H Takeuchi Y Sugawa H Ogasawara S Takaku S . RAGE activation in macrophages and development of experimental diabetic polyneuropathy. JCI Insight. (2022) 7:e160555. doi: 10.1172/jci.insight.160555 36477360 PMC9746912

[100] Dobi A Bravo SB Veeren B Paradela-Dobarro B Álvarez E Meilhac O . Advanced glycation end-products disrupt human endothelial cells redox homeostasis: new insights into reactive oxygen species production. Free Radic Res. (2019) 53:150–69. doi: 10.1080/10715762.2018.1529866 30821539

[101] Tawadros PS Powers KA Ailenberg M Birch SE Marshall JC Szaszi K . Oxidative stress increases surface toll-like receptor 4 expression in murine macrophages via ceramide generation. Shock. (2015) 44:157–65. doi: 10.1097/SHK.0000000000000392 25944793

[102] Stoian A Muntean C Babă DF Manea A Dénes LRN Simon-Szabó ZN . Update on biomarkers of chronic inflammatory processes underlying diabetic neuropathy. Int J Mol Sci. (2024) 25:10395. doi: 10.3390/ijms251910395 39408723 PMC11476795

[103] Alvarado-Vázquez PA Grosick RL Moracho-Vilrriales C Ward E Threatt T Romero-Sandoval EA . Cytokine production capabilities of human primary monocyte-derived macrophages from patients with diabetes mellitus type 2 with and without diabetic peripheral neuropathy. J Pain Res. (2019) 12:69–81. doi: 10.2147/JPR.S186372 30588081 PMC6305162

[104] Malik RA Newrick PG Sharma AK Jennings A Ah-See AK Mayhew TM . Microangiopathy in human diabetic neuropathy: relationship between capillary abnormalities and the severity of neuropathy. Diabetologia. (1989) 32:92–102. doi: 10.1007/BF00505180 2721843

[105] Estrella JS Nelson RN Sturges BK Vernau KM Williams DC LeCouteur RA . Endoneurial microvascular pathology in feline diabetic neuropathy. Microvasc Res. (2008) 75:403–10. doi: 10.1016/j.mvr.2007.12.002 18207200 PMC2413429

[106] Oliveira RB Sampaio EP Aarestrup F Teles RMB Silva TP Oliveira AL . Cytokines and Mycobacterium leprae induce apoptosis in human Schwann cells. J Neuropathol Exp Neurol. (2005) 64:882–90. doi: 10.1097/01.jnen.0000182982.09978.66 16215460

[107] Stoian A Bajko Z Maier S Cioflinc RA Grigorescu BL Moțățăianu A . High-dose intravenous immunoglobulins as a therapeutic option in critical illness polyneuropathy accompanying SARS-CoV-2 infection: a case-based review of the literature (Review). Exp Ther Med. (2021) 22:1182. doi: 10.3892/etm.2021.10616 34475972 PMC8406741

[108] Wagner R Myers RR . Schwann cells produce tumor necrosis factor alpha: expression in injured and non-injured nerves. Neuroscience. (1996) 73:625–9. doi: 10.1016/0306-4522(96)00127-3 8809782

[109] Wei S Qiu CY Jin Y Liu TT Hu WP . TNF-α acutely enhances acid-sensing ion channel currents in rat dorsal root ganglion neurons via a p38 MAPK pathway. J Neuroinflamm. (2021) 18:92. doi: 10.1186/s12974-021-02151-w 33853615 PMC8048296

[110] Leung L Cahill CM . TNF-alpha and neuropathic pain--a review. J Neuroinflamm. (2010) 7:27. doi: 10.1186/1742-2094-7-27 20398373 PMC2861665

[111] Fromont A De Seze J Fleury MC Maillefert JF Moreau T . Inflammatory demyelinating events following treatment with anti-tumor necrosis factor. Cytokine. (2009) 45:55–7. doi: 10.1016/j.cyto.2008.11.002 19109035

[112] Zhu D Fan T Huo X Cui J Cheung CW Xia Z . Progressive increase of inflammatory CXCR4 and TNF-Alpha in the dorsal root ganglia and spinal cord maintains peripheral and central sensitization to diabetic neuropathic pain in rats. Mediators Inflammation. (2019) 2019:4856156. doi: 10.1155/2019/4856156 31001066 PMC6437743

[113] Dai P Wang P Chen X Feng S Wu F Zheng X . Mesencephalic astrocyte-derived neurotrophic factor (MANF) restricts inflammatory progression through limiting macrophage infiltration in DRG and sciatic nerve during diabetic peripheral neuropathy. ACS Chem Neurosci. (2025) 16:945–59. doi: 10.1021/acschemneuro.5c00021 39970444

[114] Saleh A Smith DR Balakrishnan S Dunn L Martens C Tweed CW . Tumor necrosis factor-α elevates neurite outgrowth through an NF-κB-dependent pathway in cultured adult sensory neurons: diminished expression in diabetes may contribute to sensory neuropathy. Brain Res. (2011) 1423:87–95. doi: 10.1016/j.brainres.2011.09.029 21985959

[115] Mussa BM Srivastava A Al-Habshi A Mohammed AK Halwani R Abusnana S . Inflammatory biomarkers levels in T2DM Emirati patients with diabetic neuropathy. Diabetes Metab Syndr Obes. (2021) 14:3389–97. doi: 10.2147/DMSO.S319863 34345175 PMC8323777

[116] Ding Z Jiang M Qian J Gu D Bai H Cai M . Role of transforming growth factor-β in peripheral nerve regeneration. Neural Regener Res. (2024) 19:380–6. doi: 10.4103/1673-5374.377588 37488894 PMC10503632

[117] Bierhaus A Haslbeck KM Humpert PM Liliensiek B Dehmer T Morcos M . Loss of pain perception in diabetes is dependent on a receptor of the immunoglobulin superfamily. J Clin Invest. (2004) 114:1741–51. doi: 10.1172/JCI18058 15599399 PMC535062

[118] Choi BM Lee SH An SM Park DY Lee GW Noh GJ . The time-course and RNA interference of TNF-α, IL-6, and IL-1β expression on neuropathic pain induced by L5 spinal nerve transection in rats. Korean J Anesthesiol. (2015) 68:159–69. doi: 10.4097/kjae.2015.68.2.159 25844135 PMC4384404

[119] Chanda D Ray S Chakraborti D Sen S Mitra A . Interleukin-6 levels in patients with diabetic polyneuropathy. Cureus. (2022) 14:e21952. doi: 10.7759/cureus.21952 35155045 PMC8820488

[120] Cotter MA Gibson TM Nangle MR Cameron NE . Effects of interleukin-6 treatment on neurovascular function, nerve perfusion and vascular endothelium in diabetic rats. Diabetes Obes Metab. (2010) 12:689–99. doi: 10.1111/j.1463-1326.2010.01221.x 20590746

[121] Andriambeloson E Baillet C Vitte PERA Garotta G Dreano M Callizot N . Interleukin-6 attenuates the development of experimental diabetes-related neuropathy. Neuropathology. (2006) 26:32–42. doi: 10.1111/j.1440-1789.2006.00651.x 16521477

[122] Cox AA Sagot Y Hedou G Grek C Wilkes T Vinik AI . Low-dose pulsatile interleukin-6 as a treatment option for diabetic peripheral neuropathy. Front Endocrinol (Lausanne). (2017) 8:89. doi: 10.3389/fendo.2017.00089 28512447 PMC5411416

[123] Magrinelli F Briani C Romano M Ruggero S Toffanin E Triolo G . The association between serum cytokines and damage to large and small nerve fibers in diabetic peripheral neuropathy. J Diabetes Res. (2015) 2015:547834. doi: 10.1155/2015/547834 25961054 PMC4415740

[124] Hinder LM Murdock BJ Park M Bender DE O'Brien PD Rumora AE . Transcriptional networks of progressive diabetic peripheral neuropathy in the db/db mouse model of type 2 diabetes: an inflammatory story. Exp Neurol. (2018) 305:33–43. doi: 10.1016/j.expneurol.2018.03.011 29550371 PMC5955815

[125] McGregor BA Eid S Rumora AE Murdock B Guo K de Anda-Jáuregui G . Conserved transcriptional signatures in human and murine diabetic peripheral neuropathy. Sci Rep. (2018) 8:17678. doi: 10.1038/s41598-018-36098-5 30518872 PMC6281650

[126] Siqueira Mietto B Kroner A Girolami EI Santos-Nogueira E Zhang J David S . Role of IL-10 in resolution of inflammation and functional recovery after peripheral nerve injury. J Neurosci. (2015) 35:16431–42. doi: 10.1523/JNEUROSCI.2119-15.2015 26674868 PMC6605511

[127] Yanik BM Dauch JR Cheng HT . Interleukin-10 reduces neurogenic inflammation and pain behavior in a mouse model of type 2 diabetes. J Pain Res. (2020) 13:3499–512. doi: 10.2147/JPR.S264136 33402846 PMC7778525

[128] de Souza S Rosario Claudio JS Sim J Inyang KE Dagenais A Monahan K . Interleukin-10 signaling in somatosensory neurons controls CCL2 release and inflammatory response. Brain Behav Immun. (2024) 116:193–202. doi: 10.1016/j.bbi.2023.12.013 38081433 PMC10843623

[129] Hakim S Jain A Adamson SS Petrova V Indajang J Kim HW . Macrophages protect against sensory axon loss in peripheral neuropathy. Nature. (2025) 640:212–20. doi: 10.1038/s41586-024-08535-1 39939762 PMC11964918

[130] Pugliese G Iacobini C Pesce CM Menini S . Galectin-3: an emerging all-out player in metabolic disorders and their complications. Glycobiology. (2015) 25:136–50. doi: 10.1093/glycob/cwu111 25303959

[131] Conti G Scarpini E Baron P Livraghi S Tiriticco M Bianchi R . Macrophage infiltration and death in the nerve during the early phases of experimental diabetic neuropathy: a process concomitant with endoneurial induction of IL-1beta and p75NTR. J Neurol Sci. (2002) 195:35–40. doi: 10.1016/s0022-510x(01)00684-0 11867071

[132] Gonçalves NDAP Jager SE Richner M Murray SS Mohseni S Jensen TS . Schwann cell p75 neurotrophin receptor modulates small fiber degeneration in diabetic neuropathy. Glia. (2020) 68:2725–43. doi: 10.1002/glia.23881 32658363

[133] Chen G Luo X Wang W Wang Y Zhu F Wang W . Interleukin-1β promotes Schwann cells de-differentiation in Wallerian degeneration via the c-JUN/AP-1 pathway. Front Cell Neurosci. (2019) 13:304. doi: 10.3389/fncel.2019.00304 31338026 PMC6629865

[134] Zhu X Zhu J . CD4 T helper cell subsets and related human immunological disorders. Int J Mol Sci. (2020) 21:8011. doi: 10.3390/ijms21218011 33126494 PMC7663252

[135] Yang X Tang W Shi YQ . Detection and clinical significance of regulatory T cells in peripheral blood of elderly patients with type 2 diabetic peripheral neuropathy. Academic Journal of Naval Medical University. (2015) 36:1007–1011. doi: 10.3724/SP.J.1008.2015.01007

[136] Bi JH Hao LX Zhou J Huang F Wang CL Liu YM . The level and significance of CD4+CD25+Foxp3+ Treg in peripheral blood of patients with type 2 diabetic peripheral neuropathy. Journal of Chinese Physician. (2021) 23:1523–1527. doi: 10.3760/cma.j.cn431274-20200915-01290 30704229

[137] Hui Y Kuang L Zhong Y Tang Y Xu Z Zheng T . High glucose impairs cognitive function through inducing mitochondrial calcium overload in Treg cells. Iscience. (2024) 27:108689. doi: 10.1016/j.isci.2023.108689 38226157 PMC10788441

[138] Zhu CL Deng Y Zhang YL Chen L . Expression and Correlation Analysis of Peripheral Blood Th17-Related Cytokines and CD4+ Regulatory T Lymphocytes in Patients with Diabetic Peripheral Neuropathy. Prac. Clin. Med. (2025) 26:11–14. doi: 10.13764/j.cnki.lcsy.2025.01.003

[139] Kimura A Kishimoto T . IL-6: regulator of Treg/Th17 balance. Eur J Immunol. (2010) 40:1830–5. doi: 10.1002/eji.201040391 20583029

[140] Zheng YH Ren CY Shen Y Li JB Chen MW . A cross-sectional study on the correlation between inflammatory cytokines, negative emotions, and onset of peripheral neuropathy in type 2 diabetes. Neuropsychiatr Dis Treat. (2020) 16:2881–90. doi: 10.2147/NDT.S278439 33293813 PMC7718991

[141] Qiu AOW Bian Z Mao PA Liu QH . IL-17A exacerbates diabetic retinopathy by impairing Müller cell function via Act1 signaling. Exp Mol Med. (2016) 48:e280. doi: 10.1038/emm.2016.117 27980343 PMC5192073

[142] Zheng MY Luo LZ . The role of IL-17A in mediating inflammatory responses and progression of neurodegenerative diseases. Int J Mol Sci. (2025) 26:2505. doi: 10.3390/ijms26062505 40141149 PMC11941770

[143] Jiang X Zhou R Zhang Y Zhu T Li Q Zhang W . Interleukin-17 as a potential therapeutic target for chronic pain. Front Immunol. (2022) 13:999407. doi: 10.3389/fimmu.2022.999407 36248896 PMC9556763

[144] Habash T Saleh A Roy Chowdhury SK Smith DR Fernyhough P . The proinflammatory cytokine, interleukin-17A, augments mitochondrial function and neurite outgrowth of cultured adult sensory neurons derived from normal and diabetic rats. Exp Neurol. (2015) 273:177–89. doi: 10.1016/j.expneurol.2015.08.016 26321687

[145] El-Samahy MH Tantawy AAG Adly AAM Habeeb NM Ismail EAR Hamed GM . Expression of CD4(+) CD28(null) T lymphocytes in children and adolescents with type 1 diabetes mellitus: relation to microvascular complications, aortic elastic properties, and carotid intima media thickness. Pediatr Diabetes. (2017) 18:785–93. doi: 10.1111/pedi.12484 28102614

[146] Dumitriu IE . The life (and death) of CD4+ CD28(null) T cells in inflammatory diseases. Immunology. (2015) 146:185–93. doi: 10.1111/imm.12506 26190355 PMC4582960

[147] 余筱燕 汤珂珂 吕迪 . 阻滞Kv1.3通道抑制CD4+ CD28null T细胞活性缓解糖尿病微血管损伤的实验研究. 中华全科医学. (2019) 17:1335–9. doi: 10.16766/j.cnki.issn.1674-4152.000937

[148] 金瑶 刘方芳 李明 李昕倡 贾真 余雪松 . 细颗粒物对血管内皮细胞炎症因子表达的影响和研究白藜芦醇的干预作用. 中国临床药理学杂志. (2018) 34:1073–6. doi: 10.13699/j.cnki.1001-6821.2018.09.019

[149] Tang W Lv Q Chen XF Zou JJ Liu ZM Shi YQ . CD8(+) T cell-mediated cytotoxicity toward Schwann cells promotes diabetic peripheral neuropathy. Cell Physiol Biochem. (2013) 32:827–37. doi: 10.1159/000354485 24080983

[150] Ge S Xie J Zheng L Yang L Zhu H Cheng X . Associations of serum anti-ganglioside antibodies and inflammatory markers in diabetic peripheral neuropathy. Diabetes Res Clin Pract. (2016) 115:68–75. doi: 10.1016/j.diabres.2016.02.005 27242125

[151] Janahi NM Santos D Blyth C Bakhiet M Ellis M . Diabetic peripheral neuropathy, is it an autoimmune disease? Immunol Lett. (2015) 168:73–9. doi: 10.1016/j.imlet.2015.09.009 26386377

[152] Willison HJ . Anti-ganglioside antibodies in peripheral nerve pathology. Methods Mol Biol. (2018) 1804:173–88. doi: 10.1007/978-1-4939-8552-4_7 29926408

[153] Kleffel S Vergani A Tezza S Ben Nasr M Niewczas MA Wong S . Interleukin-10+ regulatory B cells arise within antigen-experienced CD40+ B cells to maintain tolerance to islet autoantigens. Diabetes. (2015) 64:158–71. doi: 10.2337/db13-1639 25187361 PMC4274804

[154] Ellis JS Braley-Mullen H . Mechanisms by which B cells and regulatory T cells influence development of murine organ-specific autoimmune diseases. J Clin Med. (2017) 6:13. doi: 10.3390/jcm6020013 28134752 PMC5332917

[155] Serreze DV Fleming SA Chapman HD Richard SD Leiter EH Tisch RM . B lymphocytes are critical antigen-presenting cells for the initiation of T cell-mediated autoimmune diabetes in nonobese diabetic mice. J Immunol. (1998) 161:3912–8. doi: 10.4049/jimmunol.161.8.3912 9780157

[156] Kalampokis I Yoshizaki A Tedder TF . IL-10-producing regulatory B cells (B10 cells) in autoimmune disease. Arthritis Res Ther. (2013) 15 Suppl 1:S1. doi: 10.1186/ar3907 23566714 PMC3624502

[157] Mielle J Audo R Hahne M Macia L Combe B Morel J . IL-10 producing B cells ability to induce regulatory T cells is maintained in rheumatoid arthritis. Front Immunol. (2018) 9:961. doi: 10.3389/fimmu.2018.00961 29774031 PMC5943500

[158] Nashtahosseini Z Eslami M Paraandavaji E Haraj A Dowlat BF Hosseinzadeh E . Cytokine signaling in diabetic neuropathy: a key player in peripheral nerve damage. Biomedicines. (2025) 13:589. doi: 10.3390/biomedicines13030589 40149566 PMC11940495

[159] Prager I Watzl C . Mechanisms of natural killer cell-mediated cellular cytotoxicity. J Leukoc Biol. (2019) 105:1319–29. doi: 10.1002/JLB.MR0718-269R 31107565

[160] Lassen J Stürner KH Gierthmühlen J Dargvainiene J Kixmüller D Leypoldt F . Protective role of natural killer cells in neuropathic pain conditions. Pain. (2021) 162:2366–75. doi: 10.1097/j.pain.0000000000002274 33769361

[161] Yao X Wang X Zhang R Kong L Fan C Qian Y . Dysregulated mast cell activation induced by diabetic milieu exacerbates the progression of diabetic peripheral neuropathy in mice. Nat Commun. (2025) 16:4170. doi: 10.1038/s41467-025-59562-z 40325050 PMC12052842

[162] Lo J Clare-Salzler MJ . Dendritic cell subsets and type I diabetes: focus upon DC-based therapy. Autoimmun Rev. (2006) 5:419–23. doi: 10.1016/j.autrev.2005.12.001 16890897

[163] Chiu IM Heesters BA Ghasemlou N Von Hehn CA Zhao F Tran J . Bacteria activate sensory neurons that modulate pain and inflammation. Nature. (2013) 501:52–7. doi: 10.1038/nature12479 23965627 PMC3773968

[164] Njeim R Azar WS Fares AH Azar ST Kfoury Kassouf H Eid AA . NETosis contributes to the pathogenesis of diabetes and its complications. J Mol Endocrinol. (2020) 65:R65–76. doi: 10.1530/JME-20-0128 33048064

[165] Rezaei Shahrabi A Arsenault G Nabipoorashrafi SA Lucke-Wold B Yaghoobpoor S Meidani FZ . Relationship between neutrophil to lymphocyte ratio and diabetic peripheral neuropathy: a systematic review and meta-analysis. Eur J Med Res. (2023) 28:523. doi: 10.1186/s40001-023-01479-8 37974254 PMC10652461

[166] Iype J Fux M . Basophils orchestrating eosinophils' chemotaxis and function in allergic inflammation. Cells. (2021) 10:895. doi: 10.3390/cells10040895 33919759 PMC8070740

[167] Lee KH Kim JE Park CJ . Peripheral neuropathy associated with hypereosinophilic syndrome. Ann Dermatol. (2008) 20:149–52. doi: 10.5021/ad.2008.20.3.149 27303181 PMC4903968

[168] Nakamura Y Fukutomi Y Sekiya K Kajiwara K Kawasaki Y Fujita N . Low-dose mepolizumab is effective as an add-on therapy for treating long-lasting peripheral neuropathy in patients with eosinophilic granulomatosis with polyangiitis. Mod Rheumatol. (2022) 32:387–95. doi: 10.1093/mr/roab005 34910206

[169] Ransohoff RM Cardona AE . The myeloid cells of the central nervous system parenchyma. Nature. (2010) 468:253–62. doi: 10.1038/nature09615 21068834

[170] Lanlua P Prommahom A Sricharoenvej S . Increased number of activated microglia in rat spinal cord during early stage of diabetic induction. Folia Morphol (Warsz). (2020) 79:662–71. doi: 10.5603/FM.a2019.0136 31886881

[171] Koepsell H . Glucose transporters in brain in health and disease. Pflugers Arch. (2020) 472:1299–343. doi: 10.1007/s00424-020-02441-x 32789766 PMC7462931

[172] Wang Q Xie Y Ma S Luo H Qiu Y . Role of microglia in diabetic neuropathic pain. Front Cell Dev Biol. (2024) 12:1421191. doi: 10.3389/fcell.2024.1421191 39135776 PMC11317412

[173] Miyoshi K Obata K Kondo T Okamura H Noguchi K . Interleukin-18-mediated microglia/astrocyte interaction in the spinal cord enhances neuropathic pain processing after nerve injury. J Neurosci. (2008) 28:12775–87. doi: 10.1523/JNEUROSCI.3512-08.2008 19036970 PMC6671812

[174] Abhilasha A Mitra P Suri S Saxena I Shukla R Shukla KK . Increased expression of serum IL-18 and IL-18R in newly diagnosed type 2 diabetes mellitus. Minerva Endocrinol (Torino). (2023) 48:35–41. doi: 10.23736/S2724-6507.20.03271-X 33103874

[175] Tsuda M Shigemoto-Mogami Y Koizumi S Mizokoshi A Kohsaka S Salter MW . P2X4 receptors induced in spinal microglia gate tactile allodynia after nerve injury. Nature. (2003) 424:778–83. doi: 10.1038/nature01786 12917686

[176] Milne R Brownstein S . Advanced glycation end products and diabetic retinopathy. Amino Acids. (2013) 44:1397–407. doi: 10.1007/s00726-011-1071-3 21909978

[177] Zhou R Yazdi AS Menu P Tschopp JR . A role for mitochondria in NLRP3 inflammasome activation. Nature. (2011) 469:221–5. doi: 10.1038/nature09663 21124315

[178] Li Y Kong E Ding R Chu R Lu J Deng M . Hyperglycemia-induced Sirt3 downregulation increases microglial aerobic glycolysis and inflammation in diabetic neuropathic pain pathogenesis. CNS Neurosci Ther. (2024) 30:e14913. doi: 10.1111/cns.14913 39123294 PMC11315676

[179] Yang J Yu Z Jiang Y Zhang Z Tian Y Cai J . SIRT3 alleviates painful diabetic neuropathy by mediating the FoxO3a-PINK1-Parkin signaling pathway to activate mitophagy. CNS Neurosci Ther. (2024) 30:e14703. doi: 10.1111/cns.14703 38572816 PMC10993345

[180] Kawai T Akira S . The role of pattern-recognition receptors in innate immunity: update on Toll-like receptors. Nat Immunol. (2010) 11:373–84. doi: 10.1038/ni.1863 20404851

[181] Sun SY Yan QQ Qiao LN Shi YN Tan LH Yang YS . Electroacupuncture alleviates pain responses and inflammation in collagen-induced arthritis rats via suppressing the TLR2/4-MyD88-NF-κB signaling pathway. Evid Based Complement Alternat Med. (2023) 2023:9050763. doi: 10.1155/2023/9050763 36785752 PMC9922193

[182] Hung HC Tsai SF Sie SR Kuo YM . High glucose enhances lipopolysaccharide-induced inflammation in cultured BV2 microglial cell line. Immun Inflammation Dis. (2022) 10:e610. doi: 10.1002/iid3.610 35478445 PMC9017628

[183] Chou WC Rampanelli E Li X Ting JPY . Impact of intracellular innate immune receptors on immunometabolism. Cell Mol Immunol. (2022) 19:337–51. doi: 10.1038/s41423-021-00780-y 34697412 PMC8891342

[184] Palsson-McDermott EM Curtis AM Goel G Lauterbach MAR Sheedy FJ Gleeson LE . Pyruvate kinase M2 regulates Hif-1α activity and IL-1β induction and is a critical determinant of the warburg effect in LPS-activated macrophages. Cell Metab. (2015) 21:65–80. doi: 10.1016/j.cmet.2014.12.005 25565206 PMC5198835

[185] Michalek RD Gerriets VA Jacobs SR Macintyre AN MacIver NJ Mason EF . Cutting edge: distinct glycolytic and lipid oxidative metabolic programs are essential for effector and regulatory CD4+ T cell subsets. J Immunol. (2011) 186:3299–303. doi: 10.4049/jimmunol.1003613 21317389 PMC3198034

[186] Shi LZ Wang R Huang G Vogel P Neale G Green DR . HIF1alpha-dependent glycolytic pathway orchestrates a metabolic checkpoint for the differentiation of TH17 and Treg cells. J Exp Med. (2011) 208:1367–76. doi: 10.1084/jem.20110278 21708926 PMC3135370

[187] Gubser PM Bantug GR Razik L Fischer M Dimeloe S Hoenger G . Rapid effector function of memory CD8+ T cells requires an immediate-early glycolytic switch. Nat Immunol. (2013) 14:1064–72. doi: 10.1038/ni.2687 23955661

[188] Wang Z Guan D Wang S Chai LYA Xu S Lam KP . Glycolysis and oxidative phosphorylation play critical roles in natural killer cell receptor-mediated natural killer cell functions. Front Immunol. (2020) 11:202. doi: 10.3389/fimmu.2020.00202 32153568 PMC7045049

[189] Doughty CA Bleiman BF Wagner DJ Dufort FJ Mataraza JM Roberts MF . Antigen receptor-mediated changes in glucose metabolism in B lymphocytes: role of phosphatidylinositol 3-kinase signaling in the glycolytic control of growth. Blood. (2006) 107:4458–65. doi: 10.1182/blood-2005-12-4788 16449529 PMC1895797

[190] Malandrino MI Fucho R Weber MI Calderon-Dominguez MA Mir JF Valcarcel L . Enhanced fatty acid oxidation in adipocytes and macrophages reduces lipid-induced triglyceride accumulation and inflammation. Am J Physiol Endocrinol Metab. (2015) 308:E756–69. doi: 10.1152/ajpendo.00362.2014 25714670

[191] Patsoukis N Bardhan K Chatterjee P Sari D Liu B Bell LN . PD-1 alters T-cell metabolic reprogramming by inhibiting glycolysis and promoting lipolysis and fatty acid oxidation. Nat Commun. (2015) 6:6692. doi: 10.1038/ncomms7692 25809635 PMC4389235

[192] van der Windt GJW O'Sullivan D Everts B Huang SCC Buck MD Curtis JD . CD8 memory T cells have a bioenergetic advantage that underlies their rapid recall ability. Proc Natl Acad Sci USA. (2013) 110:14336–41. doi: 10.1073/pnas.1221740110 23940348 PMC3761631

[193] Ecker J Liebisch G Englmaier M Grandl M Robenek H Schmitz G . Induction of fatty acid synthesis is a key requirement for phagocytic differentiation of human monocytes. Proc Natl Acad Sci USA. (2010) 107:7817–22. doi: 10.1073/pnas.0912059107 20385828 PMC2867858

[194] Krawczyk CM Holowka T Sun J Blagih J Amiel E DeBerardinis RJ . Toll-like receptor-induced changes in glycolytic metabolism regulate dendritic cell activation. Blood. (2010) 115:4742–9. doi: 10.1182/blood-2009-10-249540 20351312 PMC2890190

[195] Everts B Amiel E Huang SCC Smith AM Chang CH Lam WY . TLR-driven early glycolytic reprogramming via the kinases TBK1-IKKε supports the anabolic demands of dendritic cell activation. Nat Immunol. (2014) 15:323–32. doi: 10.1038/ni.2833 24562310 PMC4358322

[196] Cluxton D Petrasca A Moran B Fletcher JM . Differential regulation of human Treg and Th17 cells by fatty acid synthesis and glycolysis. Front Immunol. (2019) 10:115. doi: 10.3389/fimmu.2019.00115 30778354 PMC6369198

[197] Wang C Yosef N Gaublomme J Wu C Lee Y Clish CB . CD5L/AIM regulates lipid biosynthesis and restrains Th17 cell pathogenicity. Cell. (2015) 163:1413–27. doi: 10.1016/j.cell.2015.10.068 26607793 PMC4671820

[198] Lee J Walsh MC Hoehn KL James DE Wherry EJ Choi Y . Regulator of fatty acid metabolism, acetyl coenzyme a carboxylase 1, controls T cell immunity. J Immunol. (2014) 192:3190–9. doi: 10.4049/jimmunol.1302985 24567531 PMC3965631

[199] Dufort FJ Gumina MR Ta NL Tao Y Heyse SA Scott DA . Glucose-dependent de novo lipogenesis in B lymphocytes: a requirement for atp-citrate lyase in lipopolysaccharide-induced differentiation. J Biol Chem. (2014) 289:7011–24. doi: 10.1074/jbc.M114.551051 24469453 PMC3945362

[200] Costigan M Moss A Latremoliere A Johnston C Verma-Gandhu M Herbert TA . T-cell infiltration and signaling in the adult dorsal spinal cord is a major contributor to neuropathic pain-like hypersensitivity. J Neurosci. (2009) 29:14415–22. doi: 10.1523/JNEUROSCI.4569-09.2009 19923276 PMC2813708

[201] Strand N Anderson MA Attanti S Gill B Wie C Dawodu A . Diabetic neuropathy: pathophysiology review. Curr Pain Headache Rep. (2024) 28:481–7. doi: 10.1007/s11916-024-01243-5 38558164

[202] Visperas A Do JS Bulek K Li X Min B . IL-27, targeting antigen-presenting cells, promotes Th17 differentiation and colitis in mice. Mucosal Immunol. (2014) 7:625–33. doi: 10.1038/mi.2013.82 24129161 PMC3989480

[203] Iwamoto S Iwai SI Tsujiyama K Kurahashi C Takeshita K Naoe M . TNF-alpha drives human CD14+ monocytes to differentiate into CD70+ dendritic cells evoking Th1 and Th17 responses. J Immunol. (2007) 179:1449–57. doi: 10.4049/jimmunol.179.3.1449 17641010

[204] Mills KHG . Induction, function and regulation of IL-17-producing T cells. Eur J Immunol. (2008) 38:2636–49. doi: 10.1002/eji.200838535 18958872

[205] Davies AJ Rinaldi S Costigan M Oh SB . Cytotoxic immunity in peripheral nerve injury and pain. Front Neurosci. (2020) 14:142. doi: 10.3389/fnins.2020.00142 32153361 PMC7047751

[206] Davies AJ Kim HW Gonzalez-Cano R Choi J Back SK Roh SE . Natural killer cells degenerate intact sensory afferents following nerve injury. Cell. (2019) 176:716–28. doi: 10.1016/j.cell.2018.12.022 30712871 PMC6418410

[207] Davoli-Ferreira M de Lima KA Fonseca MM Guimarães RM Gomes FI Cavallini MC . Regulatory T cells counteract neuropathic pain through inhibition of the Th1 response at the site of peripheral nerve injury. Pain. (2020) 161:1730–43. doi: 10.1097/j.pain.0000000000001879 32701834

[208] Austin PJ Kim CF Perera CJ Moalem-Taylor G . Regulatory T cells attenuate neuropathic pain following peripheral nerve injury and experimental autoimmune neuritis. Pain. (2012) 153:1916–31. doi: 10.1016/j.pain.2012.06.005 22789131

[209] Carr MW Roth SJ Luther E Rose SS Springer TA . Monocyte chemoattractant protein 1 acts as a T-lymphocyte chemoattractant. Proc Natl Acad Sci USA. (1994) 91:3652–6. doi: 10.1073/pnas.91.9.3652 8170963 PMC43639

[210] Du Q Fu YX Shu AM Lv X Chen YP Gao YY . Loganin alleviates macrophage infiltration and activation by inhibiting the MCP-1/CCR2 axis in diabetic nephropathy. Life Sci. (2021) 272:118808. doi: 10.1016/j.lfs.2020.118808 33245967

[211] Gao YJ Zhang L Samad OA Suter MR Yasuhiko K Xu ZZ . JNK-induced MCP-1 production in spinal cord astrocytes contributes to central sensitization and neuropathic pain. J Neurosci. (2009) 29:4096–108. doi: 10.1523/JNEUROSCI.3623-08.2009 19339605 PMC2682921

[212] Bogacka J Ciapała K Pawlik K Dobrogowski J Przeklasa-Muszynska A Mika J . Blockade of CCR4 diminishes hypersensitivity and enhances opioid analgesia - evidence from a mouse model of diabetic neuropathy. Neuroscience. (2020) 441:77–92. doi: 10.1016/j.neuroscience.2020.06.025 32592824

[213] Chatterjee P Srivastava AK Kumar DA Chakrawarty A Khan MA Ambashtha AK . Effect of deep tissue laser therapy treatment on peripheral neuropathic pain in older adults with type 2 diabetes: a pilot randomized clinical trial. BMC Geriatr. (2019) 19:218. doi: 10.1186/s12877-019-1237-5 31405365 PMC6689877

[214] Ilnytska O Lyzogubov VV Stevens MJ Drel VR Mashtalir N Pacher P . Poly(ADP-ribose) polymerase inhibition alleviates experimental diabetic sensory neuropathy. Diabetes. (2006) 55:1686–94. doi: 10.2337/db06-0067 16731831 PMC2228258

[215] Lupachyk S Watcho P Stavniichuk R Shevalye H Obrosova IG . Endoplasmic reticulum stress plays a key role in the pathogenesis of diabetic peripheral neuropathy. Diabetes. (2013) 62:944–52. doi: 10.2337/db12-0716 23364451 PMC3581201

[216] Vinik AI Nevoret MREL Casellini C Parson H . Diabetic neuropathy. Endocrinol Metab Clin North Am. (2013) 42:747–87. doi: 10.1016/j.ecl.2013.06.001 24286949

[217] Arthur-Farraj PJ Latouche M Wilton DK Quintes S Chabrol E Banerjee A . c-Jun reprograms Schwann cells of injured nerves to generate a repair cell essential for regeneration. Neuron. (2012) 75:633–47. doi: 10.1016/j.neuron.2012.06.021 22920255 PMC3657176

[218] Martini R Fischer S López-Vales RN David S . Interactions between Schwann cells and macrophages in injury and inherited demyelinating disease. Glia. (2008) 56:1566–77. doi: 10.1002/glia.20766 18803324

[219] Cattin AL Burden JJ Van Emmenis L Mackenzie FE Hoving JJA Garcia Calavia N . Macrophage-induced blood vessels guide Schwann cell-mediated regeneration of peripheral nerves. Cell. (2015) 162:1127–39. doi: 10.1016/j.cell.2015.07.021 26279190 PMC4553238

[220] Abd Razak NH Idris J Hassan NH Zaini F Muhamad N Daud MF . Unveiling the role of Schwann cell plasticity in the pathogenesis of diabetic peripheral neuropathy. Int J Mol Sci. (2024) 25:10785. doi: 10.3390/ijms251910785 39409114 PMC11476695

[221] Hartlehnert M Derksen A Hagenacker T Kindermann D Schäfers M Pawlak M . Schwann cells promote post-traumatic nerve inflammation and neuropathic pain through MHC class II. Sci Rep. (2017) 7:12518. doi: 10.1038/s41598-017-12744-2 28970572 PMC5624882

[222] Wu L Wang XJ Luo X Zhang J Zhao X Chen Q . Diabetic peripheral neuropathy based on Schwann cell injury: mechanisms of cell death regulation and therapeutic perspectives. Front Endocrinol (Lausanne). (2024) 15:1427679. doi: 10.3389/fendo.2024.1427679 39193373 PMC11348392

[223] Wu J Hu H Li X . Spinal neuron-glial crosstalk and ion channel dysregulation in diabetic neuropathic pain. Front Immunol. (2025) 16:1480534. doi: 10.3389/fimmu.2025.1480534 40264787 PMC12011621

[224] Liddelow SA Guttenplan KA Clarke LE Bennett FC Bohlen CJ Schirmer L . Neurotoxic reactive astrocytes are induced by activated microglia. Nature. (2017) 541:481–7. doi: 10.1038/nature21029 28099414 PMC5404890

[225] Bahniwal M Little JP Klegeris A . High glucose enhances neurotoxicity and inflammatory cytokine secretion by stimulated human astrocytes. Curr Alzheimer Res. (2017) 14:731–41. doi: 10.2174/1567205014666170117104053 28124586

[226] Grace PM Rolan PE Hutchinson MR . Peripheral immune contributions to the maintenance of central glial activation underlying neuropathic pain. Brain Behav Immun. (2011) 25:1322–32. doi: 10.1016/j.bbi.2011.04.003 21496480

[227] Tsuda M Ueno H Kataoka A Tozaki-Saitoh H Inoue K . Activation of dorsal horn microglia contributes to diabetes-induced tactile allodynia via extracellular signal-regulated protein kinase signaling. Glia. (2008) 56:378–86. doi: 10.1002/glia.20623 18186080

[228] Wang D Couture RJ Hong Y . Activated microglia in the spinal cord underlies diabetic neuropathic pain. Eur J Pharmacol. (2014) 728:59–66. doi: 10.1016/j.ejphar.2014.01.057 24508519

[229] Richner M Ferreira N Dudele A Jensen TS Vaegter CB Gonçalves NDAP . Functional and structural changes of the blood-nerve-barrier in diabetic neuropathy. Front Neurosci. (2018) 12:1038. doi: 10.3389/fnins.2018.01038 30692907 PMC6339909

[230] Giannini C Dyck PJ . Basement membrane reduplication and pericyte degeneration precede development of diabetic polyneuropathy and are associated with its severity. Ann Neurol. (1995) 37:498–504. doi: 10.1002/ana.410370412 7717686

[231] Chapouly C Yao Q Vandierdonck S Larrieu-Lahargue F Mariani JN Gadeau APER . Impaired Hedgehog signalling-induced endothelial dysfunction is sufficient to induce neuropathy: implication in diabetes. Cardiovasc Res. (2016) 109:217–27. doi: 10.1093/cvr/cvv263 26645982

[232] Wada R Yagihashi S . Role of advanced glycation end products and their receptors in development of diabetic neuropathy. Ann N Y Acad Sci. (2005) 1043:598–604. doi: 10.1196/annals.1338.067 16037282

[233] Dewanjee S Das S Das AK Bhattacharjee N Dihingia A Dua TK . Molecular mechanism of diabetic neuropathy and its pharmacotherapeutic targets. Eur J Pharmacol. (2018) 833:472–523. doi: 10.1016/j.ejphar.2018.06.034 29966615

[234] Zhang J Shi XQ Echeverry S Mogil JS De Koninck Y Rivest S . Expression of CCR2 in both resident and bone marrow-derived microglia plays a critical role in neuropathic pain. J Neurosci. (2007) 27:12396–406. doi: 10.1523/JNEUROSCI.3016-07.2007 17989304 PMC6673247

[235] Cernea S Raz I . Management of diabetic neuropathy. Metabolism. (2021) 123:154867. doi: 10.1016/j.metabol.2021.154867 34411554

[236] Elafros MA Andersen H Bennett DL Savelieff MG Viswanathan V Callaghan BC . Towards prevention of diabetic peripheral neuropathy: clinical presentation, pathogenesis, and new treatments. Lancet Neurol. (2022) 21:922–36. doi: 10.1016/S1474-4422(22)00188-0 36115364 PMC10112836

[237] Jiang S Zhang L Zhao L Lv Y Cheng S Liu C . Immunological mechanisms and therapeutic advances in diabetic neuropathy. J Neurol. (2026) 273:105. doi: 10.1007/s00415-025-13594-z 41609860

[238] Griebeler ML Morey-Vargas OL Brito JP Tsapas A Wang Z Carranza Leon BG . Pharmacologic interventions for painful diabetic neuropathy: an umbrella systematic review and comparative effectiveness network meta-analysis. Ann Intern Med. (2014) 161:639–49. doi: 10.7326/M14-0511 25364885

[239] Bialas P Maier C Klose P Häuser W . Efficacy and harms of long-term opioid therapy in chronic non-cancer pain: systematic review and meta-analysis of open-label extension trials with a study duration ≥26 weeks. Eur J Pain. (2020) 24:265–78. doi: 10.1002/ejp.1496 31661587

[240] Pålsson-McDermott EM O'Neill LAJ . Targeting immunometabolism as an anti-inflammatory strategy. Cell Res. (2020) 30:300–14. doi: 10.1038/s41422-020-0291-z 32132672 PMC7118080

[241] Diaz A Romero M Vazquez T Lechner S Blomberg BB Frasca D . Metformin improves *in vivo* and *in vitro* B cell function in individuals with obesity and Type-2 Diabetes. Vaccine. (2017) 35:2694–700. doi: 10.1016/j.vaccine.2017.03.078 28392139 PMC5560851

[242] Xiong W Sun KY Zhu Y Zhang X Zhou YH Zou X . Metformin alleviates inflammation through suppressing FASN-dependent palmitoylation of Akt. Cell Death Dis. (2021) 12:934. doi: 10.1038/s41419-021-04235-0 34642298 PMC8511025

[243] Kelly B Tannahill GM Murphy MP O'Neill LAJ . Metformin inhibits the production of reactive oxygen species from NADH:Ubiquinone oxidoreductase to limit induction of interleukin-1β (IL-1β) and boosts interleukin-10 (IL-10) in lipopolysaccharide (LPS)-activated macrophages. J Biol Chem. (2015) 290:20348–59. doi: 10.1074/jbc.M115.662114 26152715 PMC4536441

[244] Naznin F Sakoda H Okada T Tsubouchi H Waise TMZ Arakawa K . Canagliflozin, a sodium glucose cotransporter 2 inhibitor, attenuates obesity-induced inflammation in the nodose ganglion, hypothalamus, and skeletal muscle of mice. Eur J Pharmacol. (2017) 794:37–44. doi: 10.1016/j.ejphar.2016.11.028 27876617

[245] Chang SOY Kim DB Ryu GR Ko SH Jeong IK Ahn YBE . Exendin-4 inhibits iNOS expression at the protein level in LPS-stimulated Raw264.7 macrophage by the activation of cAMP/PKA pathway. J Cell Biochem. (2013) 114:844–53. doi: 10.1002/jcb.24425 23097217

[246] Himeno T Kamiya H Naruse K Harada N Ozaki N Seino Y . Beneficial effects of exendin-4 on experimental polyneuropathy in diabetic mice. Diabetes. (2011) 60:2397–406. doi: 10.2337/db10-1462 21810596 PMC3161330

[247] Brock C Hansen CS Karmisholt J Møller HJ Juhl A Farmer AD . Liraglutide treatment reduced interleukin-6 in adults with type 1 diabetes but did not improve established autonomic or polyneuropathy. Br J Clin Pharmacol. (2019) 85:2512–23. doi: 10.1111/bcp.14063 31338868 PMC6848951

[248] Hammes HP Du X Edelstein D Taguchi T Matsumura T Ju Q . Benfotiamine blocks three major pathways of hyperglycemic damage and prevents experimental diabetic retinopathy. Nat Med. (2003) 9:294–9. doi: 10.1038/nm834 12592403

[249] Berrone E Beltramo E Solimine C Ape AU Porta M . Regulation of intracellular glucose and polyol pathway by thiamine and benfotiamine in vascular cells cultured in high glucose. J Biol Chem. (2006) 281:9307–13. doi: 10.1074/jbc.M600418200 16452468

[250] Bozic I Savic D Stevanovic I Pekovic S Nedeljkovic N Lavrnja I . Benfotiamine upregulates antioxidative system in activated BV-2 microglia cells. Front Cell Neurosci. (2015) 9:351. doi: 10.3389/fncel.2015.00351 26388737 PMC4559599

[251] Stracke H Gaus W Achenbach U Federlin K Bretzel RG . Benfotiamine in diabetic polyneuropathy (BENDIP): results of a randomised, double blind, placebo-controlled clinical study. Exp Clin Endocrinol Diabetes. (2008) 116:600–5. doi: 10.1055/s-2008-1065351 18473286

[252] Rochette L Ghibu SLA Richard C Zeller M Cottin Y Vergely C . Direct and indirect antioxidant properties of α-lipoic acid and therapeutic potential. Mol Nutr Food Res. (2013) 57:114–25. doi: 10.1002/mnfr.201200608 23293044

[253] Rochette L Ghibu S Muresan A Vergely C . Alpha-lipoic acid: molecular mechanisms and therapeutic potential in diabetes. Can J Physiol Pharmacol. (2015) 93:1021–7. doi: 10.1139/cjpp-2014-0353 26406389

[254] Hsieh RY Huang IC Chen C Sung JY . Effects of oral alpha-lipoic acid treatment on diabetic polyneuropathy: a meta-analysis and systematic review. Nutrients. (2023) 15:3634. doi: 10.3390/nu15163634 37630823 PMC10458197

[255] Amato Nesbit S Sharma R Waldfogel JM Zhang A Bennett WL Yeh HC . Non-pharmacologic treatments for symptoms of diabetic peripheral neuropathy: a systematic review. Curr Med Res Opin. (2019) 35:15–25. doi: 10.1080/03007995.2018.1497958 30114983

[256] Li QR Wang Z Zhou W Fan SR Ma R Xue L . Epalrestat protects against diabetic peripheral neuropathy by alleviating oxidative stress and inhibiting polyol pathway. Neural Regener Res. (2016) 11:345–51. doi: 10.4103/1673-5374.177745 27073391 PMC4811002

[257] Giannoukakis N . Ranirestat as a therapeutic aldose reductase inhibitor for diabetic complications. Expert Opin Investig Drugs. (2008) 17:575–81. doi: 10.1517/13543784.17.4.575 18363521

[258] Hotta N Akanuma Y Kawamori R Matsuoka K Oka Y Shichiri M . Long-term clinical effects of epalrestat, an aldose reductase inhibitor, on diabetic peripheral neuropathy: the 3-year, multicenter, comparative Aldose Reductase Inhibitor-Diabetes Complications Trial. Diabetes Care. (2006) 29:1538–44. doi: 10.2337/dc05-2370 16801576

[259] Bril V Hirose T Tomioka S Buchanan R . Ranirestat for the management of diabetic sensorimotor polyneuropathy. Diabetes Care. (2009) 32:1256–60. doi: 10.2337/dc08-2110 19366965 PMC2699746

[260] Kim H Sasaki T Maeda K Koya D Kashiwagi A Yasuda H . Protein kinase Cbeta selective inhibitor LY333531 attenuates diabetic hyperalgesia through ameliorating cGMP level of dorsal root ganglion neurons. Diabetes. (2003) 52:2102–9. doi: 10.2337/diabetes.52.8.2102 12882929

[261] Bansal D Badhan Y Gudala K Schifano F . Ruboxistaurin for the treatment of diabetic peripheral neuropathy: a systematic review of randomized clinical trials. Diabetes Metab J. (2013) 37:375–84. doi: 10.4093/dmj.2013.37.5.375 24199167 PMC3816139

[262] Sima AAF . Acetyl-L-carnitine in diabetic polyneuropathy: experimental and clinical data. CNS Drugs. (2007) 21 Suppl 1:13–23, 45-46. doi: 10.2165/00023210-200721001-00003 17696589

[263] Li S Chen X Li Q Du J Liu Z Peng Y . Effects of acetyl-L-carnitine and methylcobalamin for diabetic peripheral neuropathy: a multicenter, randomized, double-blind, controlled trial. J Diabetes Investig. (2016) 7:777–85. doi: 10.1111/jdi.12493 27180954 PMC5009142

[264] Rolim LC Da Silva EM Flumignan RL Abreu MM Dib SRA . Acetyl-L-carnitine for the treatment of diabetic peripheral neuropathy. Cochrane Database Syst Rev. (2019) 6:CD011265. doi: 10.1002/14651858.CD011265.pub2 31201734 PMC6953387

[265] Li R Wu Y Zou S Wang X Li Y Xu K . NGF attenuates high glucose-induced ER stress, preventing Schwann cell apoptosis by activating the PI3K/Akt/GSK3β and ERK1/2 pathways. Neurochem Res. (2017) 42:3005–18. doi: 10.1007/s11064-017-2333-6 28762104

[266] Li L Yu T Yu L Li H Liu Y Wang D . Exogenous brain-derived neurotrophic factor relieves pain symptoms of diabetic rats by reducing excitability of dorsal root ganglion neurons. Int J Neurosci. (2016) 126:749–58. doi: 10.3109/00207454.2015.1057725 26441011

[267] Thakur V Gonzalez M Pennington K Chattopadhyay M . Viral vector mediated continuous expression of interleukin-10 in DRG alleviates pain in type 1 diabetic animals. Mol Cell Neurosci. (2016) 72:46–53. doi: 10.1016/j.mcn.2016.01.006 26802537

[268] Miyano K Ikehata M Ohshima K Yoshida Y Nose Y Yoshihara SI . Intravenous administration of human mesenchymal stem cells derived from adipose tissue and umbilical cord improves neuropathic pain via suppression of neuronal damage and anti-inflammatory actions in rats. PloS One. (2022) 17:e0262892. doi: 10.1371/journal.pone.0262892 35157707 PMC8843230

[269] Yang H Wu L Deng H Chen Y Zhou H Liu M . Anti-inflammatory protein TSG-6 secreted by bone marrow mesenchymal stem cells attenuates neuropathic pain by inhibiting the TLR2/MyD88/NF-κB signaling pathway in spinal microglia. J Neuroinflamm. (2020) 17:154. doi: 10.1186/s12974-020-1731-x 32393298 PMC7216552

[270] Brini AT Amodeo G Ferreira LM Milani A Niada S Moschetti G . Therapeutic effect of human adipose-derived stem cells and their secretome in experimental diabetic pain. Sci Rep. (2017) 7:9904. doi: 10.1038/s41598-017-09487-5 28851944 PMC5575274

[271] Omi M Hata M Nakamura N Miyabe M Ozawa S Nukada H . Transplantation of dental pulp stem cells improves long-term diabetic polyneuropathy together with improvement of nerve morphometrical evaluation. Stem Cell Res Ther. (2017) 8:279. doi: 10.1186/s13287-017-0729-5 29237486 PMC5729514

[272] Crigler L Robey RC Asawachaicharn A Gaupp D Phinney DG . Human mesenchymal stem cell subpopulations express a variety of neuro-regulatory molecules and promote neuronal cell survival and neuritogenesis. Exp Neurol. (2006) 198:54–64. doi: 10.1016/j.expneurol.2005.10.029 16336965

[273] Ko KR Lee J Lee D Nho B Kim S . Hepatocyte growth factor (HGF) promotes peripheral nerve regeneration by activating repair Schwann cells. Sci Rep. (2018) 8:8316. doi: 10.1038/s41598-018-26704-x 29844434 PMC5973939

[274] Kessler JA Smith AG Cha BS Choi SH Wymer J Shaibani A . Double-blind, placebo-controlled study of HGF gene therapy in diabetic neuropathy. Ann Clin Transl Neurol. (2015) 2:465–78. doi: 10.1002/acn3.186 26000320 PMC4435702

[275] Kessler JA Shaibani A Sang CN Christiansen M Kudrow D Vinik A . Gene therapy for diabetic peripheral neuropathy: a randomized, placebo-controlled phase III study of VM202, a plasmid DNA encoding human hepatocyte growth factor. Clin Transl Sci. (2021) 14:1176–84. doi: 10.1111/cts.12977 33465273 PMC8212761

[276] Ropper AH Gorson KC Gooch CL Weinberg DH Pieczek A Ware JH . Vascular endothelial growth factor gene transfer for diabetic polyneuropathy: a randomized, double-blinded trial. Ann Neurol. (2009) 65:386–93. doi: 10.1002/ana.21675 19399887 PMC4709012

[277] Drel VR Lupachyk S Shevalye H Vareniuk I Xu W Zhang J . New therapeutic and biomarker discovery for peripheral diabetic neuropathy: PARP inhibitor, nitrotyrosine, and tumor necrosis factor-α. Endocrinology. (2010) 151:2547–55. doi: 10.1210/en.2009-1342 20357221 PMC2875829

[278] Hashim M Badruddeen Akhtar J Khan MI Ahmad M Islam A . Diabetic neuropathy: an overview of molecular pathways and protective mechanisms of phytobioactives. Endocr Metab Immune Disord Drug Targets. (2024) 24:758–76. doi: 10.2174/0118715303266444231008143430 37867264

[279] Sood A Kumar B Singh SK Prashar P Gautam A Gulati M . Flavonoids as potential therapeutic agents for the management of diabetic neuropathy. Curr Pharm Des. (2020) 26:5468–87. doi: 10.2174/1381612826666200826164322 32851955

[280] Saikia L Barbhuiya SAA Saikia K Kalita P Dutta PP . Therapeutic potential of quercetin in diabetic neuropathy and retinopathy: exploring molecular mechanisms. Curr Top Med Chem. (2024) 24:2351–61. doi: 10.2174/0115680266330678240821060623 39253913

[281] Song W Li Y Jia Y Xu L Kang L Yang Y . Quercetin alleviates diabetic peripheral neuropathy by regulating axon guidance factors and inhibiting the Rho/ROCK pathway *in vivo* and *in vitro*. Diabetes Metab Syndr Obes. (2024) 17:4339–54. doi: 10.2147/DMSO.S491175 39582785 PMC11585991

[282] Zhang Q Song W Zhao B Xie J Sun Q Shi X . Quercetin attenuates diabetic peripheral neuropathy by correcting mitochondrial abnormality via activation of AMPK/PGC-1α pathway *in vivo* and *in vitro*. Front Neurosci. (2021) 15:636172. doi: 10.3389/fnins.2021.636172 33746703 PMC7966726

[283] Zhao B Zhang Q Liang X Xie J Sun Q . Quercetin reduces inflammation in a rat model of diabetic peripheral neuropathy by regulating the TLR4/MyD88/NF-κB signalling pathway. Eur J Pharmacol. (2021) 912:174607. doi: 10.1016/j.ejphar.2021.174607 34743981

[284] Merghany RM Saleh RA Hamed AA El-Dessouki AM El-Shiekh RA Zaki ES . Natural therapy proposed for the management of diabetic peripheral neuropathy (DPN). Inflammopharmacology. (2025). doi: 10.1007/s10787-025-01790-2 40447983

[285] Park H Lee JH Sim JH Park J Choi SS Leem JG . Effects of curcumin treatment in a diabetic neuropathic pain model of rats: involvement of c-Jun N-terminal kinase located in the astrocytes and neurons of the dorsal root ganglion. Pain Res Manag. (2021) 2021:8787231. doi: 10.1155/2021/8787231 33532012 PMC7837777

[286] Mudduluru G George-William JN Muppala S Asangani IA Kumarswamy R Nelson LD . Curcumin regulates miR-21 expression and inhibits invasion and metastasis in colorectal cancer. Biosci Rep. (2011) 31:185–97. doi: 10.1042/BSR20100065 20815812

[287] Wu H Liu Q Cai T Chen YD Wang ZF . Induction of microRNA-146a is involved in curcumin-mediated enhancement of temozolomide cytotoxicity against human glioblastoma. Mol Med Rep. (2015) 12:5461–6. doi: 10.3892/mmr.2015.4087 26239619

[288] Asadi S Gholami MS Siassi F Qorbani M Khamoshian K Sotoudeh G . Nano curcumin supplementation reduced the severity of diabetic sensorimotor polyneuropathy in patients with type 2 diabetes mellitus: a randomized double-blind placebo-controlled clinical trial. Complement Ther Med. (2019) 43:253–60. doi: 10.1016/j.ctim.2019.02.014 30935539

[289] Tian B Liu J . Resveratrol: a review of plant sources, synthesis, stability, modification and food application. J Sci Food Agric. (2020) 100:1392–404. doi: 10.1002/jsfa.10152 31756276

[290] Zhang W Yu H Lin Q Liu X Cheng Y Deng B . Anti-inflammatory effect of resveratrol attenuates the severity of diabetic neuropathy by activating the Nrf2 pathway. Aging (Albany NY). (2021) 13:10659–71. doi: 10.18632/aging.202830 33770763 PMC8064179

[291] Liu X Tang M He TY Zhao S Li HZ Li Z . Resveratrol improves paclitaxel-induced cognitive impairment in mice by activating SIRT1/PGC-1α pathway to regulate neuronal state and microglia cell polarization. Drug Des Devel Ther. (2023) 17:1125–38. doi: 10.2147/DDDT.S400936 37077409 PMC10106825

[292] Hashemi M Zandieh MA Ziaolhagh S Mojtabavi S Sadi FH Koohpar ZK . Nrf2 signaling in diabetic nephropathy, cardiomyopathy and neuropathy: therapeutic targeting, challenges and future prospective. Biochim Biophys Acta Mol Basis Dis. (2023) 1869:166714. doi: 10.1016/j.bbadis.2023.166714 37028606

[293] Osmanlıoğlu HÖ Nazıroğlu M . Resveratrol modulates diabetes-induced neuropathic pain, apoptosis, and oxidative neurotoxicity in mice through TRPV4 channel inhibition. Mol Neurobiol. (2024) 61:7269–86. doi: 10.1007/s12035-024-04311-4 38976129 PMC11339089

[294] Cui Y Li Y Ning J Mi Y Wang X Qiu Z . Resveratrol alleviates diabetic mechanical allodynia in rats by downregulating P2X3R. Mol Med Rep. (2020) 22:957–63. doi: 10.3892/mmr.2020.11157 32468070 PMC7339507

[295] Ajebli M Khan H Eddouks M . Natural alkaloids and diabetes mellitus: a review. Endocr Metab Immune Disord Drug Targets. (2021) 21:111–30. doi: 10.2174/1871530320666200821124817 32955004

[296] Dong J Zuo Z Yan W Liu W Zheng Q Liu X . Berberine ameliorates diabetic neuropathic pain in a rat model: involvement of oxidative stress, inflammation, and μ-opioid receptors. Naunyn Schmiedebergs Arch Pharmacol. (2019) 392:1141–9. doi: 10.1007/s00210-019-01659-6 31079200

[297] Yerra VG Kalvala AK Sherkhane B Areti A Kumar A . Adenosine monophosphate-activated protein kinase modulation by berberine attenuates mitochondrial deficits and redox imbalance in experimental diabetic neuropathy. Neuropharmacology. (2018) 131:256–70. doi: 10.1016/j.neuropharm.2017.12.029 29273519

[298] Gong X Gui Z Ye X Li X . Jatrorrhizine ameliorates Schwann cell myelination via inhibiting HDAC3 ability to recruit Atxn2l for regulating the NRG1-ErbB2-PI3K-AKT pathway in diabetic peripheral neuropathy mice. Phytother Res. (2023) 37:645–57. doi: 10.1002/ptr.7641 36218239

[299] Dludla PV Nkambule BB Cirilli I Marcheggiani F Mabhida SE Ziqubu K . Capsaicin, its clinical significance in patients with painful diabetic neuropathy. BioMed Pharmacother. (2022) 153:113439. doi: 10.1016/j.biopha.2022.113439 36076554

[300] Armstrong DG Bley K Simpson DM Staats P Allen S Carnevale A . Diabetic peripheral neuropathy: Pathophysiology and new insights into the mechanism of action of high-concentration topical capsaicin. J Exp Pharmacol. (2025) 17:651–65. doi: 10.2147/JEP.S526968 40948991 PMC12433221

[301] Simpson DM Robinson-Papp J Van J Stoker M Jacobs HLN Snijder RJ . Capsaicin 8% patch in painful diabetic peripheral neuropathy: A randomized, double-blind, placebo-controlled study. J Pain. (2017) 18:42–53. doi: 10.1016/j.jpain.2016.09.008 27746370

[302] Solini A Novak I . Role of the P2X7 receptor in the pathogenesis of type 2 diabetes and its microvascular complications. Curr Opin Pharmacol. (2019) 47:75–81. doi: 10.1016/j.coph.2019.02.009 30954933

[303] Ni GL Cui R Shao AM Wu ZM . Salidroside ameliorates diabetic neuropathic pain in rats by inhibiting neuroinflammation. J Mol Neurosci. (2017) 63:9–16. doi: 10.1007/s12031-017-0951-8 28741143

[304] Ben Y Hao J Zhang Z Xiong Y Zhang C Chang Y . Astragaloside IV inhibits mitochondrial-dependent apoptosis of the dorsal root ganglion in diabetic peripheral neuropathy rats through modulation of the SIRT1/p53 signaling pathway. Diabetes Metab Syndr Obes. (2021) 14:1647–61. doi: 10.2147/DMSO.S301068 33883914 PMC8055373

[305] Zhang X Liang Z Zhou Y Wang F Wei S Tan B . Artesunate inhibits apoptosis and promotes survival in Schwann cells via the PI3K/AKT/mTOR axis in diabetic peripheral neuropathy. Biol Pharm Bull. (2023) 46:764–72. doi: 10.1248/bpb.b22-00619 37258141

[306] Yang J Wei Y Zhao T Li X Zhao X Ouyang X . Magnolol effectively ameliorates diabetic peripheral neuropathy in mice. Phytomedicine. (2022) 107:154434. doi: 10.1016/j.phymed.2022.154434 36122436

[307] Gupta M Knezevic NN Abd-Elsayed A Ray M Patel K Chowdhury B . Treatment of painful diabetic neuropathy-a narrative review of pharmacological and interventional approaches. Biomedicines. (2021) 9:573. doi: 10.3390/biomedicines9050573 34069494 PMC8161066

[308] ElMeligie MM Gomaa YS Taha E Kentiba E Abdeen HAA Al-Hamaky DMA . Neuropathic pain relief through transcutaneous electrical neuromuscular stimulation: Insights from a systematic review and meta-analysis of clinical evidence. BioMed Res Int. (2025) 2025:5328365. doi: 10.1155/bmri/5328365 41132422 PMC12543013

[309] Song Z Ultenius C Meyerson BRA Linderoth B . Pain relief by spinal cord stimulation involves serotonergic mechanisms: an experimental study in a rat model of mononeuropathy. Pain. (2009) 147:241–8. doi: 10.1016/j.pain.2009.09.020 19836134

[310] Janssen SP Gerard S Raijmakers ME Truin M Van Kleef M Joosten EA . Decreased intracellular GABA levels contribute to spinal cord stimulation-induced analgesia in rats suffering from painful peripheral neuropathy: the role of KCC2 and GABA(A) receptor-mediated inhibition. Neurochem Int. (2012) 60:21–30. doi: 10.1016/j.neuint.2011.11.006 22107704

[311] van Beek M Geurts JW Slangen R Schaper NC Faber CG Joosten EA . Severity of neuropathy is associated with long-term spinal cord stimulation outcome in painful diabetic peripheral neuropathy: Five-year follow-up of a prospective two-center clinical trial. Diabetes Care. (2018) 41:32–8. doi: 10.2337/dc17-0983 29109298

[312] Liao WT Tseng CAC Chia WT Lin CR . High-frequency spinal cord stimulation treatment attenuates the increase in spinal glutamate release and spinal miniature excitatory postsynaptic currents in rats with spared nerve injury-induced neuropathic pain. Brain Res Bull. (2020) 164:307–13. doi: 10.1016/j.brainresbull.2020.09.005 32937185

[313] Petersen EA Stauss TG Scowcroft JA Brooks ES White JL Sills SM . Effect of high-frequency (10-kHz) spinal cord stimulation in patients with painful diabetic neuropathy: A randomized clinical trial. JAMA Neurol. (2021) 78:687–98. doi: 10.1001/jamaneurol.2021.0538 33818600 PMC8022268

[314] Cho E Kim W . Effect of acupuncture on diabetic neuropathy: A narrative review. Int J Mol Sci. (2021) 22:8575. doi: 10.3390/ijms22168575 34445280 PMC8395323

[315] Tang HY Wang FJ Ma JL Wang H Shen GM Jiang AJ . Acupuncture attenuates the development of diabetic peripheral neuralgia by regulating P2X4 expression and inflammation in rat spinal microglia. J Physiol Sci. (2020) 70:45. doi: 10.1186/s12576-020-00769-8 32967614 PMC10717860

[316] Shi L Zhang HH Xiao Y Hu J Xu GY . Electroacupuncture suppresses mechanical allodynia and nuclear factor κ B signaling in streptozotocin-induced diabetic rats. CNS Neurosci Ther. (2013) 19:83–90. doi: 10.1111/cns.12035 23230847 PMC6493446

[317] Yuan CX Wang X Liu Y Xu TC Yu Z Xu B . Electroacupuncture alleviates diabetic peripheral neuropathy through modulating mitochondrial biogenesis and suppressing oxidative stress. World J Diabetes. (2025) 16:93130. doi: 10.4239/wjd.v16.i2.93130 39959279 PMC11718478

[318] Li J Hu X Liang F Liu J Zhou H Liu J . Therapeutic effects of moxibustion simultaneously targeting Nrf2 and NF-κB in diabetic peripheral neuropathy. Appl Biochem Biotechnol. (2019) 189:1167–82. doi: 10.1007/s12010-019-03052-8 31209719 PMC6882806

[319] Salehi B Stojanović-Radić Z Matejić J Sharifi-Rad M Anil Kumar NV Martins NLA . The therapeutic potential of curcumin: A review of clinical trials. Eur J Med Chem. (2019) 163:527–45. doi: 10.1016/j.ejmech.2018.12.016 30553144

[320] Nathan DM Genuth S Lachin J Cleary P Crofford O Davis M . The effect of intensive treatment of diabetes on the development and progression of long-term complications in insulin-dependent diabetes mellitus. N Engl J Med. (1993) 329:977–86. doi: 10.1056/NEJM199309303291401 8366922

[321] Martin CL Albers JW Pop-Busui R . Neuropathy and related findings in the diabetes control and complications trial/epidemiology of diabetes interventions and complications study. Diabetes Care. (2014) 37:31–8. doi: 10.2337/dc13-2114 24356595 PMC3868000

[322] Ang L Jaiswal M Martin C Pop-Busui R . Glucose control and diabetic neuropathy: lessons from recent large clinical trials. Curr Diabetes Rep. (2014) 14:528. doi: 10.1007/s11892-014-0528-7 25139473 PMC5084623

[323] Charles M Ejskjaer N Witte DR Borch-Johnsen K Lauritzen T Sandbaek A . Prevalence of neuropathy and peripheral arterial disease and the impact of treatment in people with screen-detected type 2 diabetes: the ADDITION-Denmark study. Diabetes Care. (2011) 34:2244–9. doi: 10.2337/dc11-0903 21816977 PMC3177734

[324] Ismail-Beigi F Craven T Banerji MA Basile J Calles J Cohen RM . Effect of intensive treatment of hyperglycaemia on microvascular outcomes in type 2 diabetes: an analysis of the ACCORD randomised trial. Lancet. (2010) 376:419–30. doi: 10.1016/S0140-6736(10)60576-4 20594588 PMC4123233

[325] Feldman EL Callaghan BC Pop-Busui R Zochodne DW Wright DE Bennett DL . Diabetic neuropathy. Nat Rev Dis Primers. (2019) 5:41. doi: 10.1038/s41572-019-0092-1 31197153

[326] D'Souza RS Barman R Joseph A Abd-Elsayed A . Evidence-based treatment of painful diabetic neuropathy: a systematic review. Curr Pain Headache Rep. (2022) 26:583–94. doi: 10.1007/s11916-022-01061-7 35716275

[327] Backonja M Beydoun A Edwards KR Schwartz SL Fonseca V Hes M . Gabapentin for the symptomatic treatment of painful neuropathy in patients with diabetes mellitus: a randomized controlled trial. Jama. (1998) 280:1831–6. doi: 10.1001/jama.280.21.1831 9846777

[328] Freynhagen R Strojek K Griesing T Whalen E Balkenohl M . Efficacy of pregabalin in neuropathic pain evaluated in a 12-week, randomised, double-blind, multicentre, placebo-controlled trial of flexible- and fixed-dose regimens. Pain. (2005) 115:254–63. doi: 10.1016/j.pain.2005.02.032 15911152

[329] Baba M Matsui N Kuroha M Wasaki Y Ohwada S . Long-term safety and efficacy of mirogabalin in Asian patients with diabetic peripheral neuropathic pain. J Diabetes Investig. (2020) 11:693–8. doi: 10.1111/jdi.13178 31722446 PMC7232295

[330] Guo X Zhang T Yuan G Zeng W Hu Q Ma J . GABA analogue HSK16149 in Chinese patients with diabetic peripheral neuropathic pain: A phase 3 randomized clinical trial. JAMA Netw Open. (2024) 7:e2425614. doi: 10.1001/jamanetworkopen.2024.25614 39158916 PMC11333976

[331] Valenzuela-Fuenzalida JJ López-Chaparro M Barahona-Vásquez M Campos-Valdes J Cordero Gonzalez J Nova-Baeza P . Effectiveness of duloxetine versus other therapeutic modalities in patients with diabetic neuropathic pain: A systematic review and meta-analysis. Pharm (Basel). (2024) 17:856. doi: 10.3390/ph17070856 39065707 PMC11280092

[332] Gao Y Guo X Han P Li Q Yang G Qu S . Treatment of patients with diabetic peripheral neuropathic pain in China: a double-blind randomised trial of duloxetine vs. placebo. Int J Clin Pract. (2015) 69:957–66. doi: 10.1111/ijcp.12641 25939897 PMC4682474

[333] Armstrong DG Grunberger G . Stimulating results signal a new treatment option for people living with painful diabetic neuropathy. J Diabetes Sci Technol. (2023) 17:1387–91. doi: 10.1177/19322968221099542 35770993 PMC10563543

[334] Rendell MS . The time to develop treatments for diabetic neuropathy. Expert Opin Investig Drugs. (2021) 30:119–30. doi: 10.1080/13543784.2021.1868433 33423557

